# Homology of the Lateral Eyes of Scorpiones: A Six-Ocellus Model

**DOI:** 10.1371/journal.pone.0112913

**Published:** 2014-12-03

**Authors:** Stephanie F. Loria, Lorenzo Prendini

**Affiliations:** 1 Richard Gilder Graduate School, American Museum of Natural History, New York, New York, United States of America; 2 Scorpion Systematics Research Group, Division of Invertebrate Zoology, American Museum of Natural History, New York, New York, United States of America; CNRS, France

## Abstract

Scorpions possess two types of visual organs, the median and lateral eyes. Both eyes consist of simple ocelli with biconvex lenses that differ in structure and function. There is little variation in the number of median ocelli across the order. Except for a few troglomorphic species in which the median ocelli are absent, all scorpions possess a single pair. In contrast, the number of pairs of lateral ocelli varies from zero to five across Scorpiones and may vary within species. No attempt has been made to homologize lateral ocelli across the order, and their utility in scorpion systematics has been questioned, due to the variation in number. A recent study examined the number of lateral ocelli among various Asian Buthidae C.L. Koch, 1837 and proposed a “five-eye model” for the family. This model has not been examined more broadly within Buthidae, however, nor compared with the patterns of variation observed among other scorpion families. An eyespot, referred to as an accessory lateral eye, situated ventral or posteroventral to the lateral ocelli, has also been reported in some scorpions. Analysis of its structure suggests it serves a nonvisual function. We present the first comparative study of variation in the lateral ocelli across the order Scorpiones, based on examination of a broad range of exemplar species, representing all families, 160 genera (78%), 196 species (9%), and up to 12 individuals per species. We propose a six-ocellus model for Recent scorpions with four accessory ocelli observed in various taxa, homologize the individual ocelli, and correct erroneous counts in the recent literature. We also investigate the presence of the eyespot across scorpions and discover that it is more widespread than previously recognized. Future work should investigate the genetic and developmental mechanisms underlying the formation of the lateral ocelli to test the hypotheses proposed here.

## Introduction

Scorpions possess two types of visual organs, the median and lateral eyes. The median eyes comprise a single pair of simple ocelli with biconvex lenses, situated dorsomedially on the carapace. All scorpions possess median ocelli, except for 26 troglomorphic species [1]–[5] (Table 1). The lateral eyes, also known as aggregate or schizochroal eyes, comprise multiple pairs of ocelli with biconvex lenses, located anterolaterally on either side of the carapace [6]. The number of pairs of lateral ocelli ranges from zero to five across the order [1]–[4], [7], [8]. Seventeen troglomorphic species lack lateral ocelli altogether [4], [5], [9], [10] (Table 1) whereas many Buthidae C.L. Koch, 1837 possess up to five pairs [11]. The number of pairs may vary within some species [12], .

**Table 1:** Recent scorpion families, genera and species without median and/or lateral ocelli.

Family	Species	Median	Lateral
Akravidae Levy, 2007	*Akrav israchanani* Levy, 2007	abs.	abs.
Chaerilidae Pocock, 1893	*Chaerilus sabinae* Lourenço, 1995	abs.	abs.
	*Chaerilus telnovi* Lourenço, 2009	abs.	pres.
Diplocentridae Karsch, 1880	*Diplocentrus actan* Armas & Palacios-Vargas, 2002	abs.	pres.
	*Diplocentrus anophthalmus* Francke, 1977	abs.	pres.
Hormuridae Laurie, 1896	*Hormurus polisorum* (Volschenk et al., 2001)	abs./pres.	pres.
Pseudochactidae Gromov, 1998	*Vietbocap canhi* Lourenço, 2010	abs.	abs.
	*Vietbocap lao* Lourenço, 2012	abs.	abs.
	*Vietbocap thienduongensis* Lourenço & Pham, 2012	abs.	abs.
Euscorpiidae Laurie, 1896	*Troglocormus ciego* Francke, 1981	abs.	pres.
	*Troglocormus willis* Francke, 1981	abs.	pres.
Troglotayosicidae Lourenço, 2008	*Belisarius xambeui* Simon, 1879	abs.	abs.
	*Troglotayosicus hirsutus* Botero-Trujillo et al., 2012	abs.	pres.
	*Troglotayosicus humiculum* Botero-Trujillo & Francke, 2009	abs.	pres.
	*Troglotayosicus vachoni* Lourenço, 1981	abs.	pres.
Typhlochactidae Mitchell, 1971	*Alacran chamuco* Francke 2009	abs.	abs.
	*Alacran tartarus* Francke, 1982	abs.	abs.
	*Sotanochactas elliotti* (Mitchell, 1971)	abs.	abs.
	*Stygochactas granulosus* (Sissom & Cokendolpher, 1998)	abs.	abs.
	*Typhlochactas cavicola* Francke, 1986	abs.	abs.
	*Typhlochactas mitchelli* Sissom, 1988	abs.	abs.
	*Typhlochactas reddelli* Mitchell, 1968	abs.	abs.
	*Typhlochactas rhodesi* Mitchell, 1968	abs.	abs.
	*Typhlochactas sissomi* Francke et al., 2009	abs.	abs.
	*Typhlochactas sylvestris* Francke, 1986	abs.	abs.
Urodacidae Pocock, 1893	*Aops oncodactylus* Volschenk & Prendini, 2008	abs.	abs.

The median and lateral eyes of scorpions differ structurally. The dioptric apparatus of the median eyes comprises a cuticular lens, preretinal membrane, hypodermis, vitreous body, retina, postretinal membrane, and a layer of pigment cells surrounding the retina, whereas the lateral eyes possess a cuticular lens, preretinal membrane, hypodermis, retina, layer of pigment cells and postretinal membrane, without a vitreous body and focusing lens [1], . Other differences exist at the cellular level. The retinula cells of the lateral eyes do not form discrete units as in the median eyes, and their rhabdomeres form a contiguous rhabdom suggesting continuous signaling among cells [1], [7], [15]. Assembly of the optic nerve occurs inside the retina of the median eye, whereas assembly occurs outside the retina of the lateral eye. The median eyes also possess numerous (three to six) neurosecretory fibers per retinula cell whereas the lateral eyes possess approximately one per retinula cell [15].

The structural differences between the median and lateral eyes imply different functionality. Whereas the median eyes are used for acuity and spatial discrimination, the lateral eyes have poor visual acuity [1], [7], [15], [16]. Experimental studies on *Androctonus australis* (Linnaeus, 1758) suggest the lateral eyes function primarily as light detectors, perceiving differences in brightness at very low intensities, ten log units more than the median eyes [15], [16]. Therefore, lateral eyes probably serve to detect *Zeitgeber* stimuli for regulation of the circadian rhythm [16]–[18].

The number of pairs of ocelli in the lateral eyes of scorpions has decreased since Paleozoic times [12]. Early fossil scorpions possessed compound or holochroal lateral eyes comprising many ommatidia sharing a common cornea [6]. Up to 1000 ommatidia were present in *Proscorpius osborni* (Whitfield, 1885) from the Silurian [19]. The compound lateral eyes of scorpions from later in the fossil record are intermediate between truly compound and aggregate forms. The Carboniferous *Eoscorpius distinctus* Petrunkevitch, 1949 possessed roughly 100 ommatidia, each separated from the others by sclerotization, which is characteristic of an aggregate eye [6]. More recent fossils, such as *Compsoscorpius* Petrunkevitch, 1999 and *Palaeopisthacanthus* Petrunkevitch, 1913 from the Carboniferous [12], possessed only three pairs of lateral ocelli [20]–[22] like living scorpions. Such variation among extinct scorpions suggests that compound lateral eyes evolved into the aggregate eyes observed in modern scorpions by progressive loss of ocelli [6], [12], [23]–[25].

Embryological data suggest that the lateral eyes develop as the hypodermis thickens, from depressions which give rise to the ocelli. In one of few studies on the development of lateral eyes in scorpions, five depressions were described in the buthid, *Centruroides* Marx, 1890, four in a ventral plane and one in a dorsal plane [26]. The two medial depressions were the largest in the ventral plane, whereas the posterior-most depression was the smallest. The single depression in the dorsal plane was situated between the two large depressions of the ventral plane. All depressions except the posterior-most depression in the ventral plane, developed into ocelli.

An eyespot, situated posteroventral or ventral to the lateral ocelli, was reported in the buthid, *Parabuthus transvaalicus* Purcell, 1899 [27], and seven species of chaerilid, *Chaerilus* Simon, 1877 [28]–[30]. A transmission electron micrograph analysis of the eyespot of juvenile *P. transvaalicus* indicated that it is structurally similar to the lateral ocelli, possessing photoreceptor cells, arhabdomeric cells and efferent neurosecretory fibers, but lacking a dioptric apparatus and pigment granules. Therefore, although the eyespot is sensitive to light, it appears to function primarily for nonvisual information processing [27]. Based on similar structure and position, the eyespot of scorpions appears to be homologous to the rudimentary ‘accessory eye’ of the horseshoe crab, *Limulus polyphemus* Linnaeus, 1758 [7], and, presumably for this reason, was also referred to as an accessory lateral eye [27], [31]. We prefer the term ‘eyespot’ to ‘accessory lateral eye’, however, due to the differences in structure and function between this visual organ and the ocelli of scorpions. Below, we use ‘accessory’ strictly to refer to eyes in addition to the normal complement, that are rarely observed, following a tradition in use of this term in scorpion systematics, but see Melzer [31] for another opinion.

The number of pairs of lateral ocelli has long been used in scorpion systematics, to diagnose families and genera (Table 2), and more recently as characters in phylogenetic analyses [32], [33], although the utility of this character has been questioned. For example, two pairs of lateral ocelli was long considered diagnostic for Chactidae Pocock, 1893, whereas three pairs was diagnostic for Vaejovidae Thorell, 1876, despite knowledge of chactids without two pairs of lateral ocelli and vaejovids without three pairs [12], [13], [34]–[37]. The taxonomic distribution of the apomorphic states of this character prompted Stockwell [22] to dismiss it as “nearly useless at the family level” and a similar argument was advanced by Lourenço [38]. However, Prendini [39] demonstrated synapomorphies in the number of lateral ocelli at several levels within superfamily Scorpionoidea Latreille, 1802. Characters based on homology of the individual ocelli would be more informative than counts, however, which may conceal different combinations of the same number of units.

**Table 2:** Lateral ocellus counts in Recent scorpion families, according to the literature.

Family	Count: Genera
Akravidae Levy, 2007	0
Bothriuridae Simon, 1880	2: *Vachonia* Abalos, 1954
	3
Buthidae C.L. Koch, 1837	0: *Birulatus* Vachon, 1974
	2: e.g., *Karasbergia* Hewitt, 1913
	3
	5
Chactidae Pocock, 1893	2
	4
Chaerilidae Pocock, 1893	0: *Chaerilus sabinae* Lourenço, 1995; *Chaerilus telnovi* Lourenço, 2009
	2
Diplocentridae Karsch, 1880	2: *Bioculus* Stahnke, 1968; *Oiclus* Simon, 1880
	3
Euscorpiidae Laurie, 1896	2: *Troglocormus* Francke, 1981
Hemiscorpiidae Pocock, 1893	3
Heteroscorpionidae Kraepelin, 1905	2
Hormuridae Laurie, 1896	0: *Hormurus polisorum* (Volschenk et al., 2001)
	2: *Hormiops* Fage, 1933
	3
Iuridae Thorell, 1876	2 or 3: *Calchas* Birula, 1899; *Neocalchas* Yağmur, Soleglad & Kovařík, 2013
	3: *Hadruroides* Pocock, 1893; *Hadrurus* Thorell, 1876; *Hoffmannihadrurus* Fet & Soleglad, 2004; *Iurus* Thorell, 1876; *Protoiurus* Soleglad et al., 2012
	4: *Anuroctonus* Pocock, 1893; *Caraboctonus* Pocock, 1893
Pseudochactidae Gromov, 1998	0: *Vietbocap* Lourenço & Pham, 2010
	1: *Pseudochactas* Gromov, 1998; *Troglokhammouanus* Lourenço, 2007
Scorpionidae Latreille, 1802	3
Scorpiopidae Kraepelin, 1905	2: *Parascorpiops* Banks, 1928
	3
Superstitioniidae Stahnke, 1940	2: *Superstitionia* Stahnke, 1940
Troglotayosicidae Lourenço, 1998	0: *Belisarius* Simon, 1879
	2: *Troglotayosicus* Lourenço, 1981
Typhlochactidae Mitchell, 1971	0
Urodacidae Pocock, 1893	0: *Aops* Volschenk & Prendini, 2008
	2: *Urodacus* Peters, 1861
Vaejovidae Thorell, 1876	3
	3 or 4: *Uroctonus* Thorell, 1876

There have been few attempts to examine inter- and intraspecific variation in the number of lateral ocelli across the order Scorpiones and none to homologize the individual ocelli. A recent study examined the number of lateral ocelli among various Asian Buthidae and proposed a “five-eye model” for the family [11]. According to this model, the first three lateral ocelli are similar in size, and considerably larger than the fourth and fifth. The position of the fourth lateral ocellus is unstable, with three alternative positions: posterolateral, situated in the same plane as first three lateral ocelli (P1); posterior, situated on a ridge of the carapace (P2); posterodorsal, situated above a ridge of the carapace (P3). This model has not been examined more broadly within Buthidae, however, nor compared with the patterns of variation observed among other scorpion families.

We present the first comparative study of variation in lateral ocelli across the order, based on examination of a broad range of exemplar species, representing all families, 78% of the genera, 9% of the species, and up to 12 individuals per species. We propose a six-ocellus model for Recent scorpions with four accessory ocelli observed in various taxa, homologize the individual ocelli, correct erroneous counts in the literature, and investigate the presence of the eyespot across scorpions.

## Materials and Methods

Material was examined from the following collections (Appendix S1): American Museum of Natural History, New York, U.S.A. (AMNH); California Academy of Sciences, San Francisco, U.S.A. (CAS); Field Museum of Natural History, Chicago, U.S.A. (FMNH); Hebrew University of Jerusalem, Israel (HUJ); Instituto de Biología, Universidad Nacional Autónoma de México, México City (IBUNAM); Museum of Comparative of Zoology, Harvard University, Cambridge, MA, U.S.A. (MCZ); Muséum National d'Histoire Naturelle, Paris, France (MNHN); Natural History Museum, London, U.K. (BMNH); Western Australian Museum, Perth, Australia (WAM); and W. David Sissom Private Collection, Canyon, TX, U.S.A. (WDS).

In total, 519 individuals, representing all families, 160 genera (78%) and 196 exemplar species (9%) were examined and scored for the presence and relative size of the lateral ocelli (Table 3; Appendix S2). Scorpion suprageneric classification follows [33] with emendations [39]–[43]. Singletons were examined for 71 species, whereas up to 12 individuals were examined for 125 species. Ocelli were examined on both sides of the carapace to assess bilateral symmetry. Specimens were submerged in ethanol and/or examined under ultraviolet light to observe minor ocelli, which may be difficult to distinguish from granulation or infuscation when the surface of the carapace is dry. A selection of taxa, representing the patterns observed across scorpions, was imaged under visible or ultraviolet light using a MXL Microptics ML-1000 digital imaging system.

**Table 3:** Number of genera, species and individuals examined for study of lateral ocelli in Recent scorpion families.

Family	Genera	Species	Individuals
Akravidae Levy, 2007	1	1	7
Bothriuridae Simon, 1880	14	16	44
Buthidae C.L. Koch, 1837	56	60	138
Chactidae Pocock, 1893	12	16	49
Chaerilidae Pocock, 1893	1	4	18
Diplocentridae Karsch, 1880	9	12	24
Euscorpiidae Laurie, 1896	4	7	10
Hemiscorpiidae Pocock, 1893	1	1	2
Heteroscorpionidae Kraepelin, 1905	1	3	8
Hormuridae Laurie, 1896	10	14	38
Iuridae Thorell, 1876	9	11	28
Pseudochactidae Gromov, 1998	3	3	22
Scorpionidae Latreille, 1802	4	5	37
Scorpiopidae Kraepelin, 1905	4	5	11
Superstitioniidae Stahnke, 1940	1	1	4
Troglotayosicidae Lourenço, 1998	2	2	5
Typhlochactidae Mitchell, 1971	4	6	7
Urodacidae Pocock, 1893	2	3	5
Vaejovidae Thorell, 1876	22	26	62
Total	160	196	519

**Figure 1:**
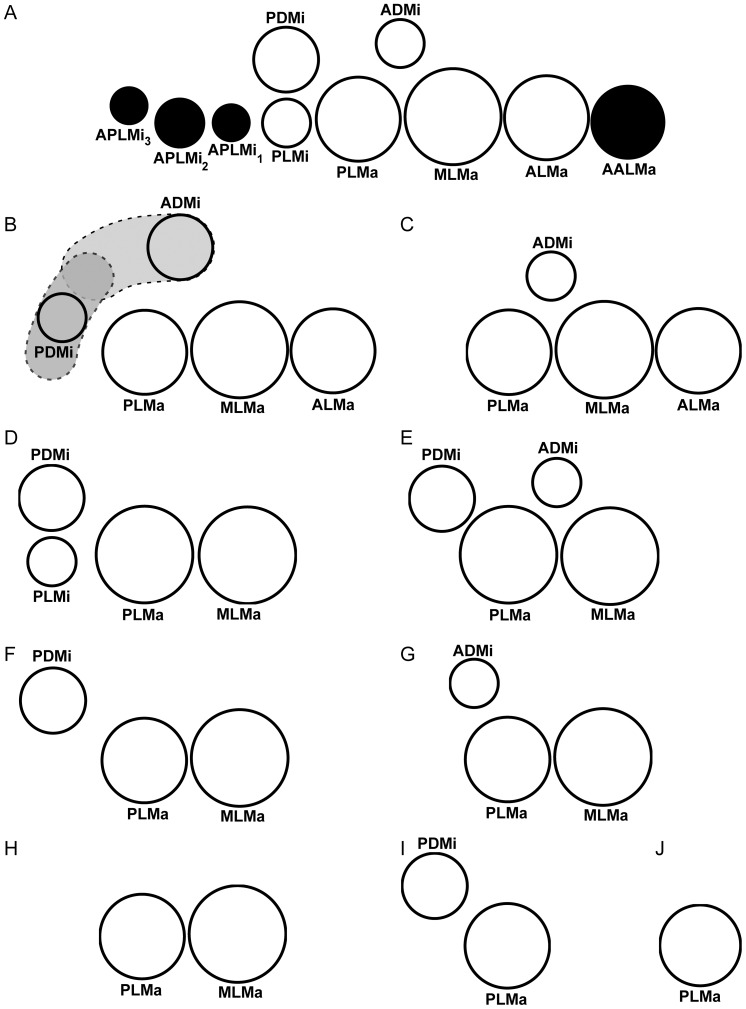
Six-ocellus model with four accessory ocelli, indicated in black (A), and nine general patterns with fewer than six ocelli (B–J), indicating most common positions and relative sizes (AALMa <ALMa <MLMa> PLMa> PDMi>ADMi; PDMi> PLMi; PLMi> APLMi_1_; PLMi  =  APLMi_2_; PLMi> APLMi_3_) of lateral ocelli in Recent scorpions. **B.** Type 5, shaded areas represent positional variation in ADMi and PDMi. **C.** Type 4A. **D.** Type 4B. **E.** Type 4C. **F.** Type 3A. **G.** Type 3B. **H.** Type 2A. **I.** Type 2B. **J.** Type 1. Abbreviations: accessory anterolateral major ocellus (AALMa); anterolateral major ocellus (ALMa); mediolateral major ocellus (MLMa); posterolateral major ocellus (PLMa); posterolateral minor ocellus (PLMi); posterodorsal minor ocellus (PDMi); anterodorsal minor ocellus (ADMi); three accessory posterolateral minor ocelli (APLMi_1_, APLMi_2_, APLMi_3_).

The terminology developed here applies to the six-ocellus model (white ocelli in Figure 1A), described below. Based on relative size and position, the six ocelli were categorized into three major and three minor ocelli: anterolateral major ocellus (ALMa); mediolateral major ocellus (MLMa); posterolateral major ocellus (PLMa); anterodorsal minor ocellus (ADMi); posterodorsal minor ocellus (PDMi); posterolateral minor ocellus (PLMi). In addition, four accessory ocelli (one major and three minor) were rarely observed, and therefore not considered part of the six-ocellus model (black ocelli in Figure 1A): accessory anterolateral major ocellus (AALMa); three accessory posterolateral minor ocelli (APLMi_1_, APLMi_2_, APLMi_3_).

The following general assumptions were applied in assigning homology to the individual ocelli. It was assumed that the ocelli are bilaterally symmetric hence the same complement of ocelli is present on the sinistral and dextral sides of the carapace of an individual, regardless of minor differences in position. When an individual was observed to possess different numbers of ocelli on the sinistral and dextral sides of the carapace, the side with fewer ocelli was considered a subset of the side with more. In individuals with the same number of ocelli on the sinistral and dextral sides of the carapace, but with the minor ocelli on one side differing in position from the other, the ocelli on both sides were regarded as homologous, regardless of positional differences. The observation of positional variation in homologous ocelli on a single individual, in turn, permitted positional variation in homologous ocelli among conspecific and heterospecific individuals, and required homology assignment to be based on relative position and size, rather than absolute position on the carapace.

## Results and Discussion

### Six-ocellus Model

**Figure 2:**
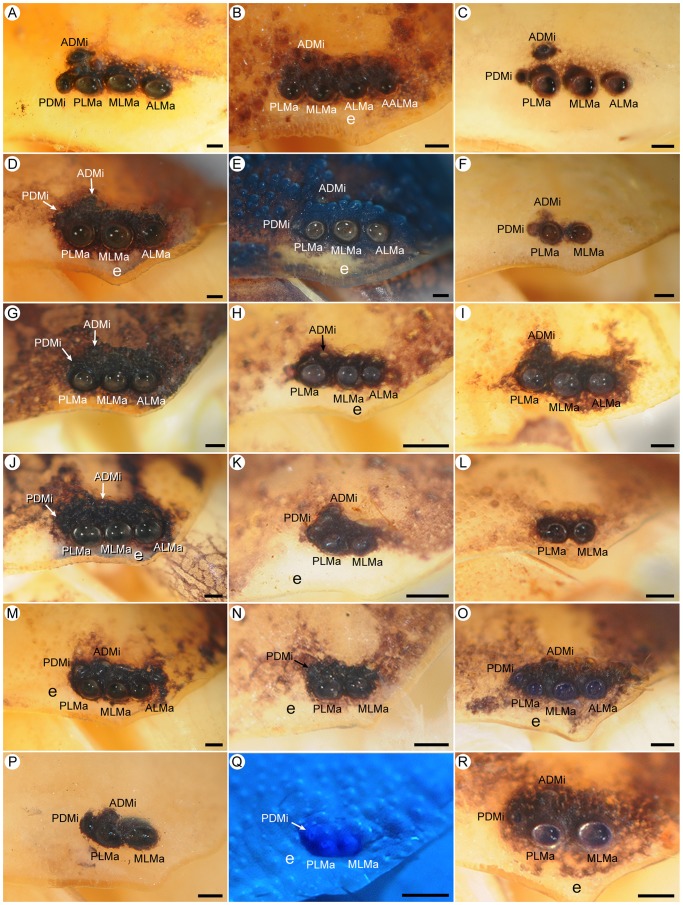
Lateral ocelli of Recent scorpions (family Buthidae C.L. Koch, 1837). **A.** *Androctonus finitimus* (Pocock, 1897), subad. ♂ (AMNH), Type 5. **B.** *Tityopsis inexpectata* (Moreno, 1940), ♀ (AMNH), abnormal six-ocellus condition with AALMa on sinistral side. **C.** *Buthacus macrocentrus* (Ehrenberg, 1828), subad. ♀ (AMNH), Type 5. **D.** *Centruroides vittatus* (Say, 1821), ♀ (AMNH), Type 5. **E.** *Orthochirus scrobiculosus* (Grube, 1873), ♀ (AMNH), Type 5. **F.** *Razianus zarudnyi* (Birula, 1903), ♀ (AMNH), Type 4C. **G.** *Tityus bahiensis eickstedtae* Lourenço, 1982, juv. (AMNH [LP 1596]), Type 5. **H.** *Alayotityus nanus* Armas, 1973, ♂ (AMNH [LP 1768]), Type 4A. **I.** *Microtityus consuelo* Armas & Marcano Fondeur, 1987, ♀ (AMNH [LP 3281]), Type 4A. **J.** *Isometrus maculatus* (DeGeer, 1778), ♂ (AMNH [LP 1798]), Type 5. **K.** *Zabius fuscus* Thorell, 1876, subad. ♀ (AMNH), Type 4C. **L.** *Akentrobuthus atakora* Vignoli & Prendini, 2008, holotype ♀ (AMNH [LP 8334]), Type 2A. **M.** *Uroplectes carinatus* (Pocock, 1890), ♂ (AMNH), Type 5. **N.** *Afroisometrus minshullae* (FitzPatrick, 1994), ♀ (AMNH [LP 7875]), Type 3A. **O.** *Pseudolychas ochraceus* (Hirst, 1911), ♀ (AMNH), Type 5. **P.** *Lissothus bernardi* Vachon, 1948, ♀ (MNHN RS 3420), Type 4C. **Q.** *Karasbergia methueni* Hewitt, 1913, ♂ (AMNH [LP 8225]), Type 3A. **R.** *Pseudolychas ochraceus* (Hirst, 1911), ♀ (AMNH), Type 4C. Abbreviations: AALMa (accessory anterolateral major ocellus); ADMi (anterodorsal minor ocellus); ALMa (anterolateral major ocellus); e (eyespot); MLMa (mediolateral major ocellus); PDMi (posterodorsal minor ocellus); PLMa (posterolateral major ocellus). Scale bars  = 0.1 mm.

**Figure 3:**
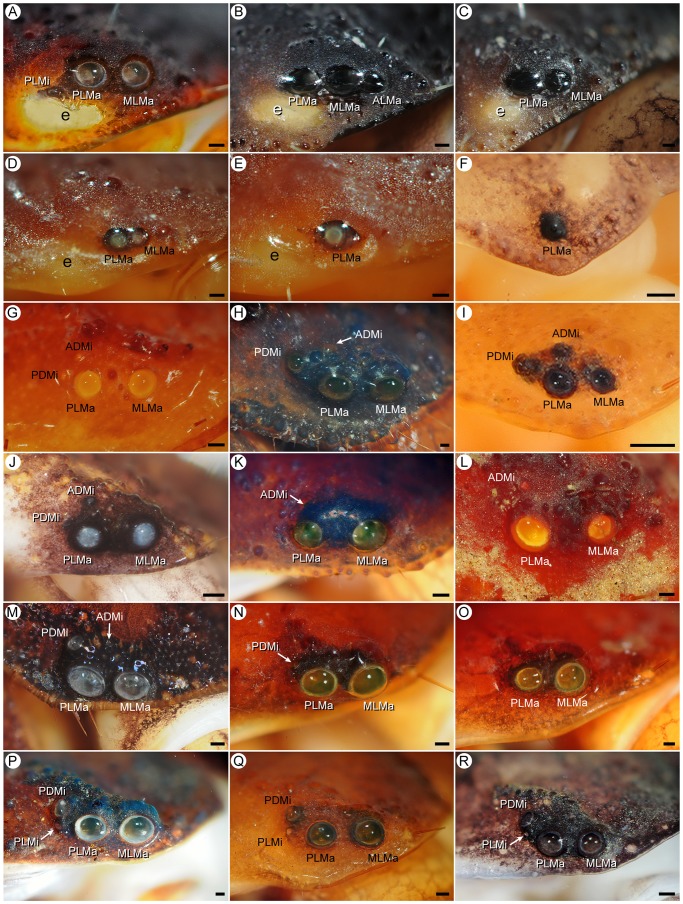
Lateral ocelli of Recent scorpions (families Chactidae Pocock, 1893; Chaerilidae, Pocock, 1893; Euscorpiidae, Laurie, 1896; Iuridae Thorell, 1876; Pseudochactidae Gromov, 1998; Scorpiopidae Kraepelin, 1905). **A.** *Chaerilus variegatus* Simon, 1877, ♀ (AMNH), abnormal three-ocellus condition with PLMi. **B.** *C. variegatus*, ♂ (AMNH [LP 6390]), abnormal three-ocellus condition with ALMa. **C.** *C. variegatus*, ♂ (AMNH [LP 6389]), Type 2A. **D, E.** *Chaerilus chapmani* Vachon & Lourenço, 1985, ♀ (AMNH), Type 2A on sinistral side (D), abnormal one-ocellus condition without MLMa (Type 1) on dextral side (E). **F.** *Pseudochactas ovchinnikovi* Gromov, 1998, subad. ♂ (AMNH), Type 1. **G.** *Troglocormus ciego* Francke, 1981, holotype ♂ (AMNH), Type 4C. **H.** *Protoiurus kraepelini* (von Ubisch, 1922), ♀ (AMNH), Type 4C. **I.** *Calchas birulai* Fet et al., 2009, ♀ (AMNH), Type 4C. **J.** *Megacormus gertschi* Díaz Najera, 1966, juv. ♂ (AMNH [LP 6474]), Type 4C. **K.** *Euscorpius italicus* (Herbst, 1800), ♂ (AMNH [LP 10297]), Type 3B. **L.** *Troglocormus willis* Francke, 1981, ♀ (AMNH), Type 3B. **M.** *Chactopsis insignis* Kraepelin, 1912, ♀ (AMNH [LP 8420]), Type 4C. **N.** *Parascorpiops montana* Banks, 1928, lectotype ♂ (MCZ), Type 3A. **O.** *P. montana*, paralectotype ♀ (MCZ), Type 2A. **P.** *Alloscorpiops* sp., ♀ (AMNH [LP 11279]), Type 4B. **Q.** *Scorpiops feti* Kovařík, 2000, ♀ (MCZ), Type 4B. **R.** *Euscorpiops kaftani* (Kovařík, 1993), juv. ♀ (AMNH [LP 11371]), Type 4B. Abbreviations: ADMi (anterodorsal minor ocellus); ALMa (anterolateral major ocellus); e (eyespot); MLMa (mediolateral major ocellus); PDMi (posterodorsal minor ocellus); PLMa (posterolateral major ocellus); PLMi (posterolateral minor ocellus). Scale bars  = 0.1 mm.

**Figure 4:**
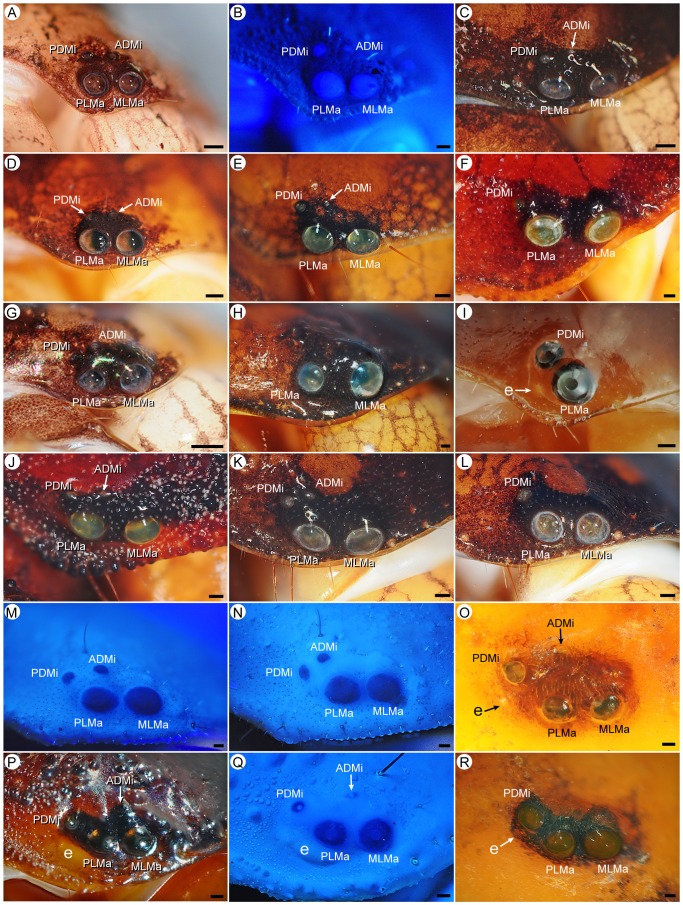
Lateral ocelli of Recent scorpions (families Chactidae Pocock, 1893; Iuridae Thorell, 1876; Troglotayosicidae Lourenço, 1998). **A.** *Megachactops kuemoi* Ochoa et al., 2013, juv. paratype (AMNH [LP 9244]), Type 4C. **B.** *Chactopsoides anduzei* (González-Sponga, 1982), ♀ (AMNH), Type 4C. **C.** *Taurepania porosus* (Pocock, 1900), ♀ (AMNH [LP 5513]), Type 4C. **D.** *Hadrurochactas machadoi* González-Sponga, 1993, subad. ♀ (AMNH), Type 4C. **E.** *Broteochactas nitidus* Pocock, 1893, ♀ (AMNH [LP 1511]), Type 4C. **F.** *Teuthraustes glaber* Kraepelin, 1912, ♀ (AMNH), Type 3A. **G.** *Vachoniochactas lasallei* (González-Sponga, 1978), subad. ♂ (AMNH [LP 10000]), Type 4C. **H.** *Chactas raymondhansi* Francke & Boos, 1986, subad. ♀ (AMNH [LP 1586]), Type 2A. **I.** *Troglotayosicus humiculum* Botero-Trujillo & Francke, 2009, ♀ (AMNH), Type 2B. **J.** *Brotheas granulatus* Simon, 1877, ♂ (AMNH [LP 3656]), Type 4C. **K, L.** *Neochactas delicatus* (Karsch, 1879), ♀ (AMNH [LP 3441]), Type 4C (**K**), Type 3A (**L**). **M, N.** *Anuroctonus phaiodactylus* (Wood, 1863), ♂ (AMNH), Type 4C, sinistral side (**M**), dextral side (**N**). **O.** *Hoffmannihadrurus gertschi* (Soleglad, 1976), paratype ♀ (AMNH), Type 4C. **P.** *Caraboctonus keyserlingii* Pocock, 1893, ♂ (AMNH), Type 4C. **Q.** *Hadruroides charcasus* (Karsch, 1879), ♂ (AMNH), Type 4C. **R.** *Hadrurus arizonensis* Ewing, 1928, ♂ (IBUNAM), Type 3A. Abbreviations: ADMi (anterodorsal minor ocellus); e (eyespot); MLMa (mediolateral major ocellus); PDMi (posterodorsal minor ocellus); PLMa (posterolateral major ocellus). Scale bars  = 0.1 mm.

**Figure 5:**
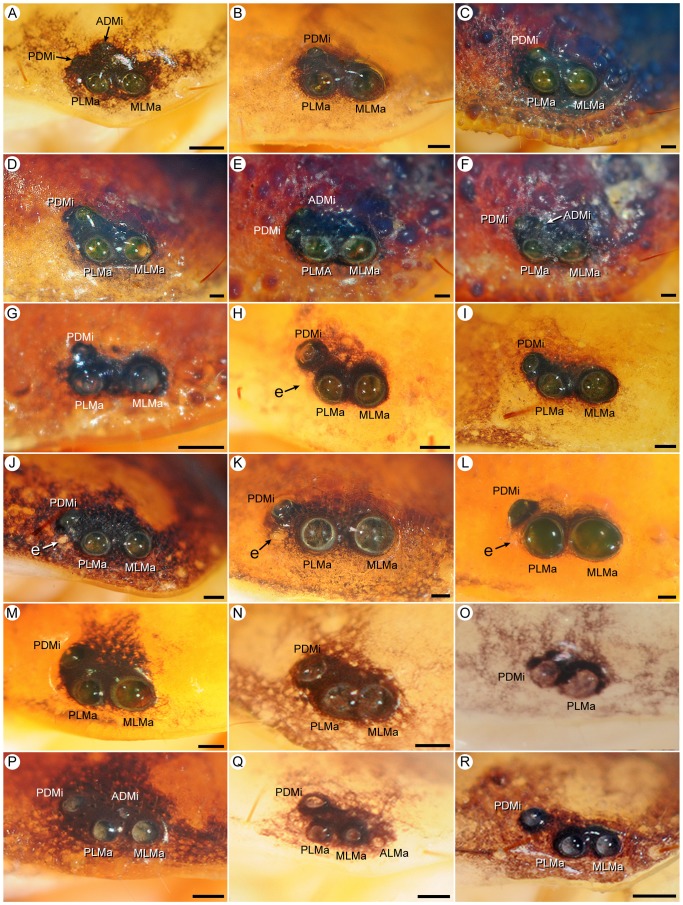
Lateral ocelli of Recent scorpions (families Bothriuridae Simon, 1880; Chactidae Pocock, 1893; Superstitioniidae Stahnke, 1940; Vaejovidae Thorell, 1876). **A.** *Superstitionia donensis* Stahnke, 1940, ♀ (AMNH), Type 4C. **B.** *Nullibrotheas allenii* (Wood, 1863), ♀ (AMNH) Type 3A. **C.** *Uroctonus mordax* Thorell, 1876, ♂ (AMNH), Type 3A. **D.** *Uroctonus mordax pluridens* Hjelle, 1972, ♀ (AMNH [LP 2686]), Type 3A. **E, F.** *U. m. pluridens*, ♂ (AMNH [LP 2686]), Type 4C, dextral side (**E**), sinistral side (**F**). **G.** *Uroctonites giulianii* Williams & Savary, 1991, ♂ (AMNH), Type 3A. **H.** *Paruroctonus surensis* Williams & Haradon, 1980, ♂ (AMNH [LP 3139]), Type 3A. **I.** *Smeringurus grandis* (Williams, 1970), juv. ♀ (AMNH [LP 4462]), Type 3A. **J.** *Vaejovis carolinianus* (Beauvois, 1805), ♀ (AMNH [LP 8500]), Type 3A. **K.** *Thorellius intrepidus* (Thorell, 1876), ♀ (AMNH [LP 2022]), Type 3A. **L.** *Stahnkeus subtilimanus* (Soleglad, 1972), ♀ (AMNH), Type 3A. **M.** *Bothriurus bonariensis* (C.L. Koch, 1842), ♀ (AMNH), Type 3A. **N.** *Timogenes mapuche* Maury, 1975, ♂ (AMNH [LP 4312]), Type 3A. **O.** *Vachonia martinezi* Abalos, 1954, juv. ♀ (AMNH [LP 2441]), Type 2B. **P.** *Centromachetes* sp., ♂ (AMNH), abnormal four-ocellus condition with ADMi (Type 4C) on dextral side. **Q.** *Lisposoma elegans* Lawrence, 1928, ♂ (AMNH), abnormal four-ocellus condition with ALMa on dextral side. **R.** *Lisposoma josehermana* Lamoral, 1979, ♂ (AMNH), Type 3A. Abbreviations: ADMi (anterodorsal minor ocellus); ALMa (anterolateral major ocellus); e (eyespot); MLMa (mediolateral major ocellus); PDMi (posterodorsal minor ocellus); PLMa (posterolateral major ocellus). Scale bars  = 0.1 mm.

**Figure 6:**
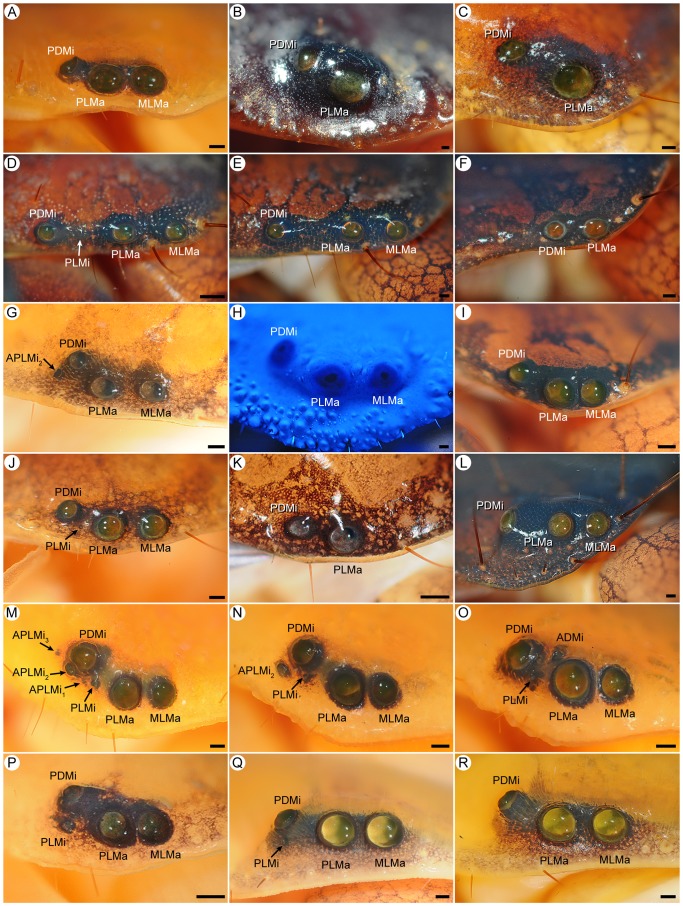
Lateral ocelli of Recent scorpions (families Diplocentridae Karsch, 1880; Hemiscorpiidae Pocock, 1893; Heteroscorpionidae Kraepelin, 1905; Hormuridae Laurie, 1896; Scorpionidae Latreille, 1802; Urodacidae Pocock, 1893). *Hemiscorpius lepturus* Peters, 1861, ♂ (AMNH [LP 11080]), Type 3A. **B.** *Heteroscorpion magnus* Lourenço & Goodman, 2002, paratype ♂ (FMNH), Type 2B. **C.** *Urodacus manicatus* (Thorell, 1876), ♀ (AMNH), Type 2B. **D.** *Cheloctonus jonesii* Pocock, 1892, ♀ (AMNH), abnormal four-ocellus condition with PLMi (Type 4B) on dextral side. **E.** *Cheloctonus jonesii* Pocock, 1892, ♀ (AMNH), Type 3A. **F.** *Hormiops davidovi* Fage, 1933, ♀ (AMNH), Type 2B. **G.** *Diplocentrus rectimanus* Karsch, 1880, ♀ (AMNH [LP 2032]), abnormal four-ocellus condition with APLMi_2_ on dextral side. **H.** *Nebo hierichonticus* (Simon, 1872), ♂ (AMNH), Type 3A. **I.** *Liocheles australasiae* (Fabricius, 1775), ♀ (AMNH), Type 3A. **J.** *Tarsoporosus macuira* Teruel & Roncallo, 2010, ♂ (AMNH), Type 4B. **K.** *Oiclus purvesii* (Becker, 1880), ♀ (AMNH [LP 9037]), Type 2B. **L.** *Pandinus gregoryi* (Pocock, 1896), juv. ♂ (AMNH), Type 3A. **M.** *Opistophthalmus jenseni* (Lamoral, 1972), ♂ (AMNH), abnormal seven-ocellus condition with MLMa, PLMa, PLMi, PDMi, APLMi_1_, APLMi_2_ and APLMi_3_. **N, O.** *O. jenseni*, ♂ (AMNH [AH 4039]), abnormal five-ocellus condition with MLMa, PLMa, PDMi, PLMi and APLMi_2_ on sinistral side (**N**) and MLMa, PLMa, PDMi, PLMi and ADMi on dextral side (**O**). **P.** *Opistophthalmus* sp., ♂ (AMNH), Type 4B. **Q.** *Scorpio maurus palmatus* (Ehrenberg, 1828), ♂ (AMNH), Type 4B. **R.** *Scorpio maurus palmatus* (Ehrenberg, 1828), ♂ (AMNH), Type 3A. Abbreviations: anterodorsal minor ocellus (ADMi); APLMi_1_, APLMi_2_, APLMi_3_ (accessory posterolateral minor ocelli); MLMa (mediolateral major ocellus); PDMi (posterodorsal minor ocellus); PLMa (posterolateral major ocellus); PLMi (posterolateral minor ocellus). Scale bars  = 0.1 mm.

Based on the survey conducted, we propose a six-ocellus model for Scorpiones (white ocelli in Figure 1A) from which nine general patterns with fewer than six ocelli can be derived (Figures 1B–J). The various ocelli were identified based on relative position and size (Figures 2–6), as follows. Major ocelli ALMa, MLMa and PLMa, arranged in a row, were similar in size and, with few exceptions, larger than minor ocelli ADMi, PDMi, and PLMi. ADMi was smaller than and dorsal, anterodorsal or posterodorsal to PLMa, and usually smaller than and anterodorsal to PDMi. PDMi was smaller than and posterior, posterodorsal or dorsal to PLMa. PLMi was smaller than and posterior to PLMa, smaller than and ventral to PDMi, and posteroventral to ADMi. Four accessory ocelli, observed in a few taxa, were not considered part of the six-ocellus model (black ocelli in Figure 1A): AALMa was smaller than and anterior to ALMa whereas APLMi_1–3_ were smaller than and posteroventral to PDMi.

Although no individuals were observed with all ten of the abovementioned ocelli, the various ocelli were observed in different combinations across the order. The maximum number of ocelli observed in a single individual was seven in the scorpionid, *Opistophthalmus jenseni* (Lamoral, 1972): MLMa, PLMa, PDMi, PLMi, APLMi_1–3_ (Figure 6M). Other individuals of *O. jenseni* possessed ADMi (Figure 6O), giving this species the maximum number of ocelli (eight) observed in the survey (Appendix S2).

### Bilateral Symmetry and Intraspecific Variation

Most of the individuals examined were bilaterally symmetric with respect to the number of lateral ocelli. Only 58 individuals (11% of the total examined) were observed with different numbers on the sinistral and dextral sides of the carapace and, in most cases, the side with fewer ocelli was a subset of the side with more (Appendix S2). Examples include a bothriurid, *Lisposoma elegans* Lawrence, 1928, with ALMa on the dextral side of the carapace only (Figure 5Q) and a chaerilid, *Chaerilus chapmani* Vachon & Lourenço, 1995, with MLMa on the sinistral side only (Figures 3D, 3E).

Some individuals with equal numbers of ocelli on the sinistral and dextral sides of the carapace exhibited positional variation in three minor ocelli (ADMi, PDMi, PLMi) on the sinistral and dextral sides. For example, in an iurid, *Anuroctonus phaiodactylus* (Wood, 1863), ADMi and PDMi were more anteriorly situated on the sinistral side of the carapace (Figure 4M), resembling another iurid, *Protoiurus kraepelini* (Von Ubisch, 1922) (Figure 3H), than on the dextral side (Figure 4N), which resembled a vaejovid, *Uroctonus mordax pluridens* Hjelle, 1972 (Figure 5E). Another example was an *U. m. pluridens* with four ocelli on both sides of the carapace, where the positions of ADMi and PDMi differed from sinistral to dextral (Figures 5E, 5F). These observations confirm that the same complement of ocelli is usually present on the sinistral and dextral sides of the carapace of an individual, regardless of differences, and that positional variation in homologous ocelli may occur on a single individual, as well as among conspecific and heterospecific individuals. A notable exception was the scorpionid, *O. jenseni*, in which different combinations of minor ocelli were observed on the sinistral and dextral sides of the carapace (Figures 6N, 6O).

Most of the species for which multiple individuals were examined, possessed constant numbers of ocelli. However, 42 species (21% of the total examined) in 38 genera were observed with polymorphic counts (Appendix S2). In most cases of intraspecific polymorphism observed, the pattern with fewer ocelli was a subset of the pattern with more. Examples include a chaerilid, *Chaerilus variegatus* Simon, 1877, in which the presence of ALMa and PLMi were polymorphic, leading to counts of two or three ocelli on different individuals (Figures 3A–3C), and a vaejovid, *U. m. pluridens*, in which the presence of ADMi was polymorphic, leading to counts of three or four ocelli on different individuals (Figures 5D–5F).

### Nine General Patterns

Nine general patterns, representing mutually exclusive combinations of lateral ocelli, were consistently observed across multiple individuals in more than one genus. More than one combination of ocelli was rarely observed among conspecifics and was not considered a general pattern.

The nine patterns observed can be grouped by the number of ocelli, as follows (Figures 1B–J): five ocelli with one minor ocellus (PLMi) absent; four ocelli with either one major ocellus (ALMa) and one minor ocellus (ADMi or PLMi) absent or two minor ocelli (PDMi or PLMi) absent; three ocelli with one major ocellus (ALMa) and two minor ocelli (ADMi or PDMi, and PLMi) absent; two ocelli with one major ocellus (ALMa) and three minor ocelli (ADMi, PDMi and PLMi) absent or two major ocelli (ALMa and MLMa) and two minor ocelli (ADMi and PLMi) absent; one ocellus (all ocelli absent except PLMa).

Based on the survey presented here, major ocelli MLMa and PLMa are the most prevalent in scorpions and always large. ALMa is usually slightly smaller than MLMa. Among the major ocelli, ALMa is most commonly absent and PLMa most commonly present. ALMa is always coincident with MLMa and PLMa, and MLMa with PLMa.

Among the minor ocelli, PDMi is most commonly present whereas PLMi is uncommon. PLMi was observed only in Chaerilidae Pocock, 1893, Scorpiopidae Kraepelin, 1905 and the scorpionoid families Diplocentridae Karsch, 1880, Heteroscorpionidae Kraepelin, 1905, Hormuridae Laurie, 1896, and Scorpionidae Latreille, 1802. APLMi_1–3_ were observed only in Diplocentridae, Heteroscorpionidae, and Scorpionidae. The taxonomic distributions of the patterns are discussed further below.

#### Type 5: Five ocelli (ALMa, MLMa, PLMa, ADMi, PDMi), PLMi absent

This pattern, derived from the six-ocellus model by the absence of PLMi, is characterized by a row of three large, similar major ocelli (ALMa, MLMa, PLMa), with two smaller minor ocelli (ADMi, PDMi), the positions of which may vary (Figure 1B). ADMi may be situated anterodorsal, dorsal or posterodorsal to PLMa (Figures 2A, C–E, G, J, M, O) and PDMi may be situated dorsal, posterodorsal or posterior to PLMa (Figures 2A, C–E, G, J, M, O). Type 5 is restricted to Buthidae, and is the most common pattern in the family (Table 4), observed in 42 genera and 77% of the exemplar species. Two of the genera presented the pattern on only one side of the carapace of a single individual. The relative size of the two minor ocelli was also observed to vary: ADMi is smaller than PDMi in many Buthinae C.L. Koch, 1837, whereas it is larger in many Isometrinae Kraepelin, 1891, Rhopalurusinae Bücherl, 1971 and Uroplectinae Pavlovsky, 1924 (Appendix S2).

**Table 4:** General patterns (Types 5, 4A, 4B, 4C, 3A, 3B, 2A, 2B, 1) of lateral ocelli in Recent scorpion families and genera.

Akravidae Levy, 2007	
absent	*Akrav* Levy, 2007

Bothriuridae Simon, 1880	
3A	13 genera
2B	*Vachonia* Abalos, 1954
4C	single individual: *Centromachetes* Lønnberg, 1897; *Phoniocercus* Pocock, 1893

Buthidae C.L. Koch, 1837	
5	42 genera
4A	13 genera: *Ananteris* Thorell, 1891; *Charmus* Karsch, 1879; *Lychas* C.L. Koch, 1845; *Mesotityus* González-Sponga, 1981; *Microananteris* Lourenço, 2003; *Microcharmus* Lourenço, 1995; *Microtityus* Kjellesvig-Waering, 1966; rarely: *Alayotityus* Armas, 1973; *Androctonus* Ehrenberg, 1828; *Babycurus* Karsch, 1886; *Birulatus* Vachon, 1974; *Physoctonus* Mello-Leitão, 1934; *Tityus* C.L. Koch, 1836
4C	5 genera: *Lissothus* Vachon, 1948; *Microbuthus* Kraepelin, 1898; *Pseudolychas* Kraepelin, 1911; *Razianus* Farzanpay, 1987; *Zabius* Thorell, 1893
3A	2 genera: *Afroisometrus* Kovařík, 1997; *Karasbergia* Hewitt, 1913
3B	2 genera: *Alayotityus*; *Akentrobuthus* Lamoral, 1976
2A	2 genera: *Afroisometrus*; *Akentrobuthus*

Chactidae Pocock, 1893	
4C	9 genera
3A	5 genera: *Nullibrotheas* Williams, 1974; *Teuthraustes* Simon, 1878; rarely: *Broteochactas* Pocock, 1893; *Brotheas* C.L. Koch, 1837; *Neochactas* Soleglad & Fet, 2003
2A	*Chactas* Gervais, 1844

Chaerilidae Pocock 1893	
2A	*Chaerilus* Simon, 1877
1	single individual: *Chaerilus chapmani* Vachon & Lourenço, 1985

Diplocentridae Karsch, 1880	
3A	8 genera, including one species of *Bioculus*
2B	2 genera: *Bioculus* Stahnke, 1968; *Oiclus* Simon, 1880
4B	single individual: *Tarsoporosus* Francke, 1978
4C	single individual: *Nebo* Simon, 1878

Euscorpiidae Laurie, 1896	
4C	2 genera: *Megacormus* Karsch, 1881; *Troglocormus ciego* Francke, 1981
3B	2 genera: *Euscorpius* Thorell, 1876; *Troglocormus willis* Francke, 1981
2A	2 genera: *Plesiochactas* Pocock, 1900; *T. willis*

Hemiscorpiidae Pocock, 1893	
3A	*Hemiscorpius* Peters, 1861

Heteroscorpionidae Kraepelin, 1905	
2B	*Heteroscorpion* Birula, 1903

Hormuridae Laurie, 1896	
3A	10 genera
2B	*Hormiops* Fage, 1933
4B	two individuals: *Cheloctonus* Pocock, 1892; single individual: *Iomachus* Pocock, 1893

Iuridae Thorell, 1876	
4C	8 genera
3A	3 genera: *Hadrurus* Thorell, 1876; *Hoffmanihadrurus aztecus* (Pocock, 1902); rarely: *Neocalchas* Yağmur et al., 2013
3B	single individual: *Calchas* Birula, 1899

Pseudochactidae Gromov, 1998	
1	2 genera: *Pseudochactas* Gromov, 1998; *Troglokhammouanus* Lourenço, 2007
absent	*Vietbocap* Lourenço & Pham, 2010

Scorpionidae Latreille, 1802	
4B	all genera
3A	3 genera: *Opistophthalmus* C.L. Koch, 1837; *Pandinus* Thorell, 1876; *Scorpio* Linnaeus, 1758
4C	single individual: *Opistophthalmus*

Scorpiopidae Kraepelin, 1905	
4B	*Alloscorpiops* Vachon, 1980; *Euscorpiops* Vachon, 1980; *Scorpiops* Peters, 1861
3A or 2A	*Parascorpiops* Banks, 1928

Superstitioniidae Stahnke, 1940	
4C	*Superstitionia* Stahnke, 1940

Troglotayosicidae Lourenço, 1998	
2B	*Troglotayosicus* Lourenço, 1981
absent	*Belisarius* Simon, 1879

Typhlochactidae Mitchell, 1971	
absent	all genera

Urodacidae Pocock, 1893	
2B	*Urodacus* Peters, 1861
absent	*Aops* Volschenk & Prendini, 2008

Vaejovidae Thorell, 1876	
3A	all genera
4C	single individual: *Uroctonus* Thorell, 1876; *Pseudouroctonus* Stahnke, 1974
2A	single individual: *Wernerius* Soleglad & Fet, 2008

#### Type 4A: Four ocelli (ALMa, MLMa, PLMa, ADMi), PDMi, PLMi absent

This pattern, derived from Type 5 by the absence of PDMi, is characterized by a row of three large, similar major ocelli (ALMa, MLMa, PLMa) with one smaller minor ocellus (ADMi), situated anterodorsal or dorsal to PLMa (Figures 1C, 2H, I). Type 4A is also restricted to Buthidae and was observed in 13 genera and 22% of the exemplar species (Table 4). Six of the genera presented the pattern on only one side of the carapace of a single individual.

#### Type 4B: Four ocelli (MLMa, PLMa, PDMi, PLMi), ALMa, ADMi absent

This pattern, derived from the six-ocellus model by the absence of ALMa and ADMi, is characterized by two large, similar major ocelli (MLMa, PLMa) and two smaller minor ocelli (PDMi, PLMi). PDMi is smaller and posterodorsal to PLMa, but larger and dorsal or rarely posterodorsal to PLMi, and PLMi is much smaller and posterior to PLMa (Figures 1D, 3P–R, 6D, J, P, Q). Type 4B was observed in all genera and exemplar species of Scorpionidae, and three genera and 80% of the exemplar species of Scorpiopidae (Table 4). It was also observed on one side of the carapace in three individuals in two genera of Hormuridae and a single individual of Diplocentridae.

#### Type 4C: Four ocelli (MLMa, PLMa, ADMi, PDMi), ALMa, PLMi absent

This pattern, derived from Type 5 by the absence of ALMa, is characterized by two large, similar major ocelli (MLMa, PLMa) and two smaller minor ocelli (ADMi, PDMi), respectively dorsal or anterodorsal and posterodorsal or dorsal to PLMa (Figures 1E, 2F, K, P, R, 3G–J, M, 4A–E, G, J, K, M–Q, 5A, E, F, P). ADMi is usually similar or smaller, rarely larger, and anterodorsal or anterior to PDMi. Type 4C is the most common pattern among the chactoid families and was observed in Superstitioniidae Stahnke, 1940 (monotypic), 9 genera and 81% of the exemplar species of Chactidae, two genera and 29% of the exemplar species of Euscorpiidae Laurie, 1896, and 8 genera and 82% of the exemplar species of Iuridae Thorell, 1876 (Table 4). Type 4C was observed in only 5 genera and 8% of the exemplar species of Buthidae, and two genera and 8% of the exemplar species (in one genus on one side of the carapace of a single individual) of Vaejovidae. It was also observed on one side of the carapace of a single individual in two genera of Bothriuridae Simon, 1880 and one genus each of Diplocentridae and Scorpionidae.

#### Type 3A: Three ocelli (MLMa, PLMa, PDMi), ALMa, ADMi, PLMi absent

This pattern, derived from Type 4C by the absence of ADMi, is characterized by two large, similar major ocelli (MLMa, PLMa) and one smaller minor ocellus (PDMi), usually posterodorsal or dorsal, but rarely posterior, to PLMa (Figures 1F, 2N, Q, 3N, 4F, L, R, 5B–D, G–N, R, 6A, E, H, I, L, R). Type 3A is the most common pattern in Bothriuridae, observed in 13 genera and 94% of the species, and among the scorpionoid families, observed in Hemiscorpiidae Pocock, 1893, 8 genera and 83% of the exemplar species of Diplocentridae, 10 genera and 93% of the exemplar species of Hormuridae, and three genera and 80% of the exemplar species of Scorpionidae (Table 4). Among the other scorpion families, Type 3A was observed in all genera and exemplar species of Vaejovidae, five genera and 31% of the exemplar species of Chactidae, three genera and 27% of the exemplar species of Iuridae, two genera and 3% of the exemplar species of Buthidae and one genus and 20% of the exemplar species of Scorpiopidae. Two individuals in two of the genera of Chactidae and single individuals of Buthidae and Iuridae presented the Type 3A pattern on one side of the carapace.

#### Type 3B: Three ocelli (MLMa, PLMa, ADMi), ALMa, PDMi, PLMi absent

This pattern, derived from Type 4C by the absence of PDMi, is characterized by two large, similar major ocelli (MLMa, PLMa) and one smaller minor ocellus (ADMi), dorsal to PLMa (Figures 1G, 3K, L). It was observed in two genera and 43% of the exemplar species of Euscorpiidae (Table 4) and on one side of the carapace of a single individual in two genera of Buthidae and a single genus of Iuridae.

#### Type 2A: Two ocelli (MLMa, PLMa), ALMa, ADMi, PDMi, PLMi absent

This pattern, derived from Type 3A or 3B by the absence of PDMi or ADMi, respectively, is characterized by two large major ocelli (MLMa, PLMa) and no minor ocelli (Figures 1H, 2L, 3C, D, O, 4H). Type 2A is the typical pattern of Chaerilidae, observed in all exemplar species of *Chaerilus*, one genus and 6% of the exemplar species of Chactidae, two genera and 43% of the exemplar species of Euscorpiidae, one genus and 20% of the exemplar species of Scorpiopidae (Table 4). The Type 2A pattern was also observed on one side of the carapace in two individuals in two genera of Buthidae and single individual of Vaejovidae.

#### Type 2B: Two ocelli (PLMa, PDMi), ALMa, MLMa, ADMi, PLMi absent

This pattern, derived from Type 3A by the absence of MLMa, is characterized by a smaller minor ocellus (PDMi), usually situated posterodorsal to a larger major ocellus (PLMa; Figures 1I, 4I, 5O, 6B, C, K) but rarely situated posterior, in which case PDMi and PLMa are well separated (Figure 6F). This uncommon pattern was observed in Heteroscorpionidae, two genera (17% of the exemplar species) of Diplocentridae, and one genus of Bothriuridae (6% of the species), Hormuridae (7%), Troglotayosicidae Lourenço, 1998 (50%) and Urodacidae Pocock, 1893 (66%; Table 4).

#### Type 1: One ocellus (PLMa), ALMa, MLMa, ADMi, PDMi, PLMi absent

This pattern, derived from Type 2A by the absence of MLMa or from Type 2B by the absence of PDMi, is characterized by a single ocellus (PLMa; Figures 1J, 3E, F), and was restricted to two genera (66% of the exemplar species) of Pseudochactidae Gromov, 1998 (Table 4). It was also observed on one side of the carapace in a single individual of Chaerilidae (Figure 3E).

### Lateral Ocelli Absent

The absence of ocelli is derived from the Type 1 pattern by the absence of PLMa. Lateral ocelli are absent in 9 genera and 17 species of the following troglomorphic taxa (Table 1): the subfossil *Akrav israchanani* Levy, 2007 (Akravidae Levy, 2007); three species of *Vietbocap* Lourenço & Pham, 2010 (Pseudochactidae); one species of *Chaerilus* (Chaerilidae); *Belisarius xambeui* Simon, 1879 (Troglotayosicidae); ten described species in the genera *Alacran* Francke, 1982, *Sotanochactas* Francke, 1986, *Stygochactas* Vignoli & Prendini, 2009 and *Typhlochactas* Mitchell, 1971 (Typhlochactidae Mitchell, 1971); and *Aops oncodactylus* Volschenk & Prendini, 2008 (Urodacidae).

### Taxonomic Distribution of Lateral Ocelli

Among the major ocelli, PLMa is present in all scorpions with lateral ocelli whereas ALMa is absent in all families except Buthidae and, rarely, Bothriuridae (Figures 3B, 5Q), Chactidae and Chaerilidae. Among the minor ocelli, PDMi is present in most scorpion families, whereas PLMi is absent in all except Scorpiopidae, Scorpionidae and some Chaerilidae, Diplocentridae, Heteroscorpionidae and Hormuridae. Below, we summarize the patterns observed in the major scorpion clades (Table 4).

#### Buthidae

The number of pairs of lateral ocelli in Buthidae varies from two to five, with the broadest range of patterns among Recent scorpions, including Type 2A, 3A, 3B, 4A, 4C and 5. All buthids possess MLMa and PLMa, with MLMa usually larger than or similar to PLMa. The position and size of PDMi varies among buthids with five pairs of lateral ocelli (Type 5). In most Buthinae, PDMi is usually posterior to MLMa and larger than ADMi. In other buthid genera with five pairs of ocelli, PDMi is posterodorsal or rarely dorsal to MLMa and smaller than ADMi. Among buthids with fewer than five pairs of lateral ocelli, ALMa, ADMi and PDMi are commonly absent (Figure 2). Many buthids with fewer than five ocelli are small (total adult length less than 30 mm) suggesting the loss of ocelli is associated with reduction in body size. The Type 4A pattern is observed in all buthid subfamilies (Table 4) whereas the Type 4C pattern is restricted to Buthinae, Rhopalurusinae, and Uroplectinae (Figure 2). The Type 3A pattern occurs in Isometrinae and Uroplectinae (Figures 2N, 2Q). The Type 2A and 3B patterns are uncommon and observed on only one side of the carapace in three genera. The presence of ALMa, ADMi and PDMi is polymorphic in various buthids (Appendix S2), e.g., ALMa in *Pseudolychas ochraceus* (Hirst, 1911), ADMi in *Butheoloides milloti* Vachon, 1948, and PDMi in *Lychas burdoi* (Simon, 1882).

#### Basal Non-buthid Families

Except for the eyeless genus *Vietbocap*, all species of Pseudochactidae (i.e., two species of *Pseudochactas* Gromov, 1998 and the monotypic *Troglokhammouanus* Lourenço, 2007) are unique among scorpions in exhibiting the Type 1 pattern, a single pair of ocelli (Figure 3F). All species of Chaerilidae except the eyeless *Chaerilus sabinae* Lourenço, 1995 (Table 1) exhibit the Type 2A pattern, with two pairs of similar major ocelli (MLMa, PLMa; Figures 3C, D). However, one or three pairs are rarely observed due to the absence of MLMa in some troglomorphic species, e.g., *C. chapmani* (Figure 3E), or the presence of ALMa or PLMi, e.g., in *C. variegatus* (Figures 3A, B).

#### Chactoid Families

Except for twelve eyeless species of Akravidae, Typhlochactidae and *Belisarius* Simon, 1879 (Table 1), chactoid taxa exhibit two, three or four pairs of lateral ocelli, with a broad range of patterns including Type 2A, 2B, 3A, 3B, 4B and 4C. Only PLMa is consistently present and, among the chactoids which also possess MLMa, usually smaller (22 vaejovid genera, nine iurid genera, four euscorpiid genera, three chactid genera, three scorpiopid genera, one superstioniid genus), but may be similar (ten chactid genera, six vaejovid genera, two euscorpiid genera, two scorpiopid genera, one iurid genus) or slightly larger (three chactid genera, two iurid genera) than the latter. Contrary to the literature, most chactoids possess four pairs of lateral ocelli. For example, among Iuridae, only three pairs were reported for *Iurus* Thorell, 1876 and *Protoiurus* Soleglad et al., 2012, and two or three pairs for *Calchas* Birula, 1899 and *Neocalchas* Yağmur et al., 2013 [44]–[46]. However, in the survey presented here, only a single individual of *Calchas anlasi* Yağmur et al., 2013 and a single individual of *Neocalchas gruberi* (Fet et al., 2009) were observed with three lateral ocelli (PDMi and ADMi absent, respectively) on the dextral side of the carapace. Reports of five ocelli, with three minor ocelli dorsal to major ocelli MLMa and PLMa, in *Vachoniochactas* González-Sponga, 1978 [47] were not confirmed. The Type 4C pattern, with PDMi and ADMi respectively situated posterodorsal and dorsal to PLMa, and ADMi similar to or smaller than, but rarely larger than PDMi (Figures 3–5) is exhibited by all except two iurid genera, all except three chactid genera, two euscorpiid genera, *Superstitionia* Stahnke, 1940 (Superstitioniidae) and, rarely, *U. m. pluridens* (Vaejovidae). Most genera of Scorpiopidae, except *Parascorpiops* Banks, 1928, are unique among Chactoidea in exhibiting the Type 4B pattern, with ADMi absent and PLMi smaller than and ventral to PDMi (Figures 3P–3R). The Type 3A pattern, with PDMi smaller than and posterodorsal to PLMa, occurs in all vaejovid genera, five chactid genera, three iurid genera, and some individuals of *Parascorpiops* (Scorpiopidae). The Type 3B, 2A and 2B patterns are evident among relatively few chactoid taxa. Type 3B is observed in two euscorpiid genera and on one side of the carapace in an iurid genus. Type 2A is observed in one chactid genus, one euscorpiid genus, some individuals of *Parascorpiops*, and on one side of the carapace in a vaejovid genus (Figures 3O and 4H). *Troglotayosicus* Lourenço, 1981 (Troglotayosicidae) is unique among chactoids in presenting the Type 2B pattern (Figure 4I).

#### Bothriuridae

Most Bothriuridae exhibit the Type 3A pattern, with MLMa similar to or larger than PLMa, and PDMi similar to or slightly smaller, but rarely larger than PLMa (Figures 5M, N, R). The monotypic *Vachonia* Abalos, 1954 exhibits the Type 2B pattern, with MLMa absent (Figure 5O). Three individuals were observed with four pairs of ocelli on one side of the carapace: ALMa in one *L. elegans* (Figure 5Q) and ADMi in one *Centromachetes* Lønnberg, 1897 (Figure 5P) and one *Phoniocercus* Pocock, 1893.

#### Scorpionoidea

Scorpionoid taxa exhibit the Type 2B, 3A, 4B and rarely 4C patterns. Except for the eyeless *A. oncodactylus* (Table 1), all scorpionoid taxa possess PLMa and PDMi, with PDMi smaller than, or rarely similar to PLMa. Three pairs of lateral ocelli are consistently reported for Scorpionidae [32], [33], [38]. However, most scorpionids actually possess at least four pairs (MLMa, PLMa, PDMi, PLMi), exhibiting the Type 4B pattern with PLMi smaller and ventral to PDMi (Appendix S2). The Type 3A pattern, with three ocelli (PLMi absent) and PDMi posterodorsal to PLMa (Figures 6L, 6R), is dominant in Diplocentridae (Figure 6H). Only *Oiclus* Simon, 1880 and one species of *Bioculus* Stahnke, 1968 exhibit the Type 2B pattern (Figure 6K). Three individuals in the diplocentrid genera *Nebo* Simon, 1878, *Tarsoporosus* Francke, 1978, and *Diplocentrus* Peters, 1861 possess ADMi, PLMi and APLMi_2_, respectively, on one side of the carapace. Hemiscorpiidae and all except one genus of Hormuridae also exhibit the Type 3A pattern (Figure 6A). PDMi is situated posterior, rather than posterodorsal, to PLMa in several scorpionoid taxa with the Type 3A pattern (Figures 6A, 6E), presumably the result of dorsoventral compression of the carapace. The posterior position of PDMi is also observed in *Hormiops* Fage, 133, the only hormurid that exhibits the Type 2B pattern, with MLMa absent (Figure 6F). Urodacidae also exhibits the Type 2B pattern but, unlike *Hormiops*, PDMi is posterodorsal to PLMa (Figure 6C). Type 2B is also the dominant pattern in Heteroscorpionidae (Figure 6B), however PLMi, ADMi and several accessory ocelli occur in some individuals. Different combinations of minor ocelli PLMi and APLMi_1–3_ are expressed among *Opistophthalmus* C.L. Koch, 1837 individuals.

### Reexamination of the “Five-eye Model” for Buthidae

According to the “five-eye model” [11], the position of the fourth lateral ocellus (PDMi) of Buthidae is unstable, with three alternative positions: posterolateral, situated in the same plane as the first three lateral ocelli (P1); posterior, situated on a ridge of the carapace (P2); posterodorsal, situated above a ridge of the carapace (P3). The data presented here confirm this observation and further demonstrate that PDMi is usually situated in the posterolateral position (P1) in Buthinae whereas PDMi is situated in the posterior (P2) or posterodorsal (P3) positions in the other subfamilies.

### Eyespots

An eyespot was present in at least 54 (28%) species in 46 (29%) genera examined during the study and absent in 89 genera and 115 species. Including literature records [27]–[30], an eyespot has been observed in 60 scorpion species (Table 5). The presence or absence of the eyespot was usually unambiguous in taxa with dark integument and/or infuscation, but its presence or absence was impossible to verify in some taxa with pale and immaculate integument, in which it was therefore recorded as unknown (Appendix S2). The number of taxa in which the eyespot is present is thus probably an underestimate.

**Table 5:** Recent scorpion families and genera in which an eyespot is present, including observations from the literature [27]–[30].

Family	Genus	Exemplar Species
Buthidae C.L. Koch, 1837	*Afroisometrus* Kovařík, 1997	1
	*Alayotityus* Armas, 1973	1
	*Babycurus* Karsch, 1886	1
	*Butheolus* Simon, 1882	1
	*Buthoscorpio* Werner, 1936	2
	*Buthus* Leach, 1815	1
	*Centruroides* Marx, 1890	1
	*Cicileus* Vachon, 1948	1
	*Grosphus* Simon, 1880	1
	*Isometroides* Keyserling, 1885	1
	*Isometrus* Ehrenberg, 1828	2
	*Karasbergia* Hewitt, 1913	1
	*Lychas* C.L. Koch, 1845	2
	*Microbuthus* Kraepelin, 1898	1
	*Neobuthus* Hirst, 1911	1
	*Neogrosphus* Lourenço, 1995	1
	*Odonturus* Karsch, 1879	1
	*Orthochirus* Karsch, 1891	1
	*Parabuthus* Pocock, 1890	1
	*Physoctonus* Mello-Leitão, 1934	1
	*Pseudolychas* Kraepelin, 1911	1
	*Rhopalurus* Thorell, 1876	1
	*Sassanidotus* Farzanpay, 1987	1
	*Thaicharmus* Kovařík, 1995	1
	*Tityopsis* Armas, 1974	1
	*Uroplectes* Peters, 1861	1
	*Zabius* Thorell, 1893	1
Chactidae Pocock, 1893	*Nullibrotheas* Williams, 1974	1
Chaerilidae Pocock, 1893	*Chaerilus* Simon, 1877	10
Iuridae Thorell, 1876	*Caraboctonus* Pocock, 1893	1
	*Hadruroides* Pocock, 1893	1
	*Hadrurus* Thorell, 1876	1
	*Hoffmannihadrurus* Fet & Soleglad, 2004	2
Troglotayosicidae Lourenço, 1998	*Troglotayosicus* Lourenço, 1981	1
Vaejovidae Thorell, 1876	*Franckeus* Soleglad & Fet, 2005	1
	*Gertschius* Graham & Soleglad, 2007	1
	*Kochius* Soleglad & Fet, 2008	1
	*Maaykuyak* González-Santillán & Prendini, 2013	1
	*Mesomexovis* González-Santillán & Prendini, 2013	1
	*Paruroctonus* Werner, 1934	1
	*Pseudouroctonus* Stahnke, 1974	1
	*Serradigitus* Stahnke, 1974	1
	*Stahnkeus* Soleglad & Fet, 2006	1
	*Syntropis* Kraepelin, 1900	1
	*Thorellius* Soleglad & Fet, 2008	1
	*Vaejovis* C.L. Koch, 1836	2
	Total	60

A white to yellow, glabrous eyespot, situated ventral to the lateral ocelli, was identified in 27 genera and 30 species of Buthidae (Figure 2). The eyespot of Buthidae is uniquely different from those of other families, being rather elongated and situated posteroventral and ventral to the lateral ocelli. All species of *Chaerilus* examined also possessed an eyespot (Figure 3), situated posteroventral to PLMa, usually yellow in color and with a distinct, rounded shape differing from the eyespot of Buthidae. The eyespot of Chaerilidae was previously illustrated [28]–[30]. A yellow, glabrous eyespot was also identified in the iurid genera *Caraboctonus* Pocock, 1893, *Hadruroides* Pocock, 1893, *Hadrurus* Thorell, 1876, and *Hoffmannihadrurus* Fet & Soleglad, 2004 (Figure 4). A small, pale rounded eyespot, situated in approximately the same position as PLMi, ventral to PDMi, was observed in *Troglotayosicus* (Troglotayosicidae), *Nullibrotheas* Williams, 1974 (Chactidae) and twelve genera of Vaejovidae (Figure 5). The eyespot of *Troglotayosicus* was previously described as a reduced ocellus [48]–[50] but closer examination revealed that it does not possess a lens (Figure 4I).

An eyespot was absent in Pseudochactidae; the chactoid families Akravidae, Euscorpiidae, Scorpiopidae, Superstitioniidae, Typhlochactidae, all except one chactid genus, all except four iurid genera, and at least six vaejovid genera; Bothriuridae; and the scorpionoid families Diplocentridae, Hemiscorpiidae, Heteroscorpionidae, Hormuridae, Scorpionidae and Urodacidae. It also did not appear to be present in nine buthid genera (Table 5). The presence or absence of an eyespot was ambiguous in *Belisarius* (Troglotayosicidae) and the remaining genera of Buthidae and Vaejovidae surveyed.

The eyespot is apparently more common in scorpions than previously recognized, with considerable variation in shape and size. Little research has been conducted on this visual organ to date, and future research should investigate its structure and function across the order.

### Development of Lateral Ocelli

The limited embryological data available suggest that the lateral eyes develop as the hypodermis thickens, from depressions which give rise to the ocelli. Five depressions form in *Centruroides*, four in a ventral plane and one in a dorsal plane, all of which develop into ocelli except for the posterior-most depression in the ventral plane, which disappears [26]. *Centruroides*, like most buthids, exhibits the Type 5 pattern, with a row of three major ocelli (ALMa, MLMa, PLMa) and two minor ocelli (ADMi, PDMi), respectively situated dorsal and posterodorsal to PLMa. The dorsal depression observed by Parker [26] appears to correspond to ADMi whereas the anterior three ocelli in the ventral plane correspond to ALMa, MLMa, and PLMa. The small posterior-most depression in the ventral plane appears to correspond to PDMi, which is typically reduced in many New World buthid genera. It is likely that Parker [26] did not observe PDMi in the adult specimens as this ocellus may be difficult to see in many New World buthid genera because of its small size. More research is needed to determine the developmental pathways underlying the formation of the lateral ocelli.

### Systematic Utility of Lateral Ocelli

Based on the survey presented here (Appendix S2), the literature is replete with incorrect counts of lateral ocelli in scorpions (Table 2). We attribute this error to the greatly reduced size of the minor ocelli in many taxa, which are often almost indistinguishable from granulation and/or infuscation when the surface of the carapace is dry. The minor ocelli can often be identified only under a high-powered microscope when the specimen is submerged under ethanol, and it remains to be seen whether the minor ocelli of some small buthid taxa, measuring less than 20 mm in total adult length, are absent or reduced to the point of being invisible without scanning electron microscopy. Other researchers have recommended the use of ultraviolet light for detecting minor ocelli [11].

Despite controversy concerning the systematic utility of lateral ocelli [12], [13], [22], [33]–[39], we recommend their continued use in scorpion systematics. Although limited intraspecific variation is observed in the number of lateral ocelli in some taxa, it is clear that many of the patterns presented here are both phylogenetically informative and diagnostic for genera, families and even major clades of scorpions.

## Supporting Information

Appendix S1 **Lateral ocelli of Recent scorpion families: Material examined.** Abbreviations for collections as follows: American Museum of Natural History, New York, U.S.A. (AMNH); Natural History Museum, London, U.K. (BMNH); California Academy of Sciences, San Francisco, U.S.A. (CAS); Field Museum of Natural History, Chicago, IL, U.S.A. (FMNH); Hebrew University of Jerusalem, Israel (HUJ); Instituto de Biología, Universidad Nacional Autónoma de México, México City (IBUNAM); Museum of Comparative of Zoology, Harvard University, Cambridge, MA, U.S.A. (MCZ); Muséum National d'Histoire Naturelle, Paris, France (MNHN); Western Australian Museum, Perth, Australia (WAM); W. David Sissom Private Collection, Canyon, TX, U.S.A. (WDS).
(DOC)

Appendix S2 **Lateral ocelli of Recent scorpion families: Counts, presence or absence, relative size (larger than, equal to or smaller than), and presence or absence of eyespot in material examined.** Sample size (*n*) represents number of individuals per species. Counts provide the range observed in the sample for sinistral and dextral sides of carapace. Presence/absence data provide the count of individuals observed with particular ocelli or eyespot on sinistral and dextral sides of carapace. Abbreviations: AALMa (accessory anterolateral major ocellus); ADMi (anterodorsal minor ocellus); ALMa (anterolateral major ocellus); APLMi_1_, APLMi_2_, APLMi_3_ (accessory posterolateral minor ocelli); e (eyespot); MLMa (mediolateral major ocellus); PDMi (posterodorsal minor ocellus); PLMa (posterolateral major ocellus); PLMi (posterolateral minor ocellus).
(DOC)

## References

[1] Hjelle JT (1990) Anatomy and morphology. In Biology of Scorpions (GA Polised.) Stanford, CA: Stanford University Press, 9–63.

[2] Polis GA (1990) Ecology. In Biology of Scorpions (GA Polised.) Stanford, CA: Stanford University Press, 247–293.

[3] Sissom WD, Polis GA, Watt DD (1990) Field and laboratory methods. In Biology of Scorpions (GA Polised.) Stanford, CA: Stanford University Press, 445–461.

[4] Volschenk ES, Prendini L (2008) *Aops oncodactylus*, gen. et sp. nov., the first troglobitic urodacid (Urodacidae: Scorpiones), with a re-assessment of cavernicolous, troglobitic and troglomorphic scorpions. Invertebrate Systematics 22:235–257.

[5] Lourenço WR (2012) The genus *Vietbocap* Lourenço & Pham, 2010 (Scorpiones: Pseudochactidae); a proposition of a new subfamily and description of a new species from Laos. Comptes Rendus Biologies 335:232–237. 22464432 10.1016/j.crvi.2012.02.001

[6] Kjellesvig-Waering EN (1986) A restudy of the fossil Scorpionida of the world. Palaeontographica Americana 55:: 1–287. [Organized for publication by AS Caster and KE Caster, Ithaca, NY].

[7] Lehmann T, Melzer RR (2013) Looking like *Limulus*?–Retinula axons and visual neuropils of the median and lateral eyes of scorpions. Frontiers in Zoology 40:1–14. 10.1186/1742-9994-10-40 PMC3717128 23842208

[8] Prendini L, Volschenk ES, Maaliki S, Gromov AV (2006) A ‘living fossil’ from Central Asia: The morphology of *Pseudochactas ovchinnikovi* Gromov, 1998 (Scorpiones, Pseudochactidae) with comments on its phylogenetic position. Zoologischer Anzeiger 245:211–248.

[9] Mitchell RW (1968) *Typhlochactas*, a new genus of eyeless cave scorpion from Mexico (Scorpionida, Chactidae). Annales de Spéléologie 23:753–777.

[10] Sissom WD (1988) *Typhlochactas mitchelli*, a new species of eyeless montane forest litter scorpion from northeastern Oaxaca, Mexico (Chactidae, Superstitioninae, Typhlochactini). Journal of Arachnology 16:365–371.

[11] Yang XF, Norma-Rashid Y, Lourenço WR, Zhu MS (2013) True lateral eye numbers for extant buthids: A new discovery on an old character. PLoS One 8:1–10. 10.1371/journal.pone.0055125 PMC3559446 23383077

[12] Sissom WD (1990) Systematics, biogeography and paleontology. In Biology of Scorpions (GA Polis) Stanford, CA: Stanford University Press, 64–160.

[13] González-Sponga MA (1977) Rectification del caracter “ojos laterales” en varios generos de la familia Chactidae (Scorpionida) en Venezuela. Acta Biologica Venezuelica 9:303–315.

[14] Root TM (1990) Neurobiology. In Biology of Scorpions (GA Polis) Stanford, CA: Stanford University Press 341–413.

[15] Schliwa M, Fleissner G (1980) The lateral eyes of the scorpion, *Androctonus australis* . Cell and Tissue Research 206:95–114. 7357598 10.1007/BF00233611

[16] Warburg MR, Polis GA (1990) Behavioral responses, rhythms, and activity patterns. In Biology of Scorpions (GA Polis) Stanford, CA: Stanford University Press, 224–246.

[17] Fleissner G (1977) Scorpion lateral eyes: Extremely sensitive receptors of *Zeitgeber* stimuli. Journal of Comparative Morphology 118:101–108.

[18] Warburg MR (2013) The circadian rhythm and visual elements in scorpions: A review. Arthropods 4:150–158.

[19] Dunlop JA, Tetlie E, Prendini L (2008) Reinterpretation of the Silurian scorpion *Proscorpius osborni* (Whitfield): Integrating data from Palaeozoic and recent scorpions. Paleontology 51:303–320.

[20] Jeram AJ (1994) Carboniferous Orthosterni and their relationship to living scorpions. Palaeontology 37:513–550.

[21] Jeram AJ (1998) Phylogeny, classification and evolution of Silurian and Devonian scorpions. In Proceedings of the 17^th^ European Colloquium of Arachnology 1997 (PA Seldened.) Edinburgh: British Arachnological Society, 17–31.

[22] Stockwell SA (1989) Revision of the phylogeny and higher classification of scorpions (Chelicerata). PhD Dissertation, University of California, Berkeley.

[23] Bitsch C, Bitsch J (2005) Evolution of eye structure and arthropod phylogeny. Crustacean Issues 16:185–214.

[24] Paulus HF (1979) Eye structure and the monophyly of Arthropoda. In Arthropod Phylogeny (AP Gupta, ed.) New York: Van Nostrand Reinhold Co., 299–393.

[25] Paulus HF (2000) Phylogeny of the Myriapoda-Crustacea-Insecta: A new attempt using photoreceptor structure. Journal of Zoological Systematics and Evolutionary Research 38:189–208.

[26] Parker GH (1887) The eyes in scorpions. Bulletin of the Museum of Comparative Zoology 13:173–288.

[27] Spreitzer A, Melzer RR (2003) The nyphmal eyes of *Parabuthus transvaalicus* Purcell, 1899 (Buthidae): An accessory lateral eye in a scorpion. Zoologischer Anzeiger 242:137–143.

[28] Vachon M, Lourenço WR (1985) Scorpions cavernicoles du Sarawak (Bornéo). *Chaerilus chapmani* n. sp. (Chaerilidae) et *Lychas hosei* (Pocock, 1890) (Buthidae). Mémoires Biospéologiques 12:9–18.

[29] Bastawade DB (2006) Arachnida: Scorpionida, Uropygi, Schizomida, and oncopodid Opiliones (Chelicerata). Zoological Survey of India: Fauna of Arunachal Pradesh, State Fauna Series 13:449–465.

[30] Tikader BK, Bastawade DB (1983) Fauna of India. Vol 3 . Scorpions (Scorpionida: Arachnida). Calcutta: Zoological Survey of India. 671 pp.

[31] Melzer RR (2009) Persisting stemma neuropils in *Chaoborus crystallinus* (Diptera: Chaoboridae): Development and evolution of a bipartite visual system. Journal of Morphology 270:1524–1530. 19658108 10.1002/jmor.10779

[32] Soleglad ME, Fet V (2003) High-level systematics and phylogeny of the extant scorpions (Scorpiones: Orthosterni). Euscorpius 11:ii+1–175.

[33] Prendini L, Wheeler WC (2005) Scorpion higher phylogeny and classification, taxonomic anarchy and standards for peer review in online publishing. Cladistics 21:445–494. 10.1111/j.1096-0031.2005.00073.x 34892945

[34] Francke OF, Soleglad ME (1981) The family Iuridae Thorell (Arachnida, Scorpiones). Journal of Arachnology 9:233–258.

[35] Gertsch WJ, Soleglad ME (1972) Studies of North American scorpions of the genera *Uroctonus* and *Vejovis* (Scorpionida, Vejovidae). Bulletin of the American Museum of Natural History 148:551–608.

[36] Soleglad ME (1976) A revision of the scorpion subfamiliy Megacorminae (Scorpionida: Chactidae). Wasmann Journal of Biology 34:251–303.

[37] Stockwell SA (1992) Systematic observations on North American Scorpionida with a key and checklist of the families and genera. Journal of Medical Entomology 29:407–422.

[38] Lourenço WR (1989) Rétablissement de la famille des Ischnuridae, distincte des Scorpionidae Pocock, 1893, á partir de la sous-famille des Ischnurinae, Pocock, 1893. Revue Arachnologique 8:159–177.

[39] Prendini L (2000) Phylogeny and classification of the superfamily Scorpionoidea Latreille 1802 (Chelicerata, Scorpiones): An exemplar approach. Cladistics 16:1–78. 10.1111/j.1096-0031.2000.tb00348.x 34902920

[40] Vignoli V, Prendini L (2009) Systematic revision of the troglomorphic North American scorpion family Typhlochactidae (Scorpiones: Chactoidea). Bulletin of the American Museum of Natural History: 1–94.

[41] Volschenk ES, Mattoni CI, Prendini L (2008) Comparative anatomy of the mesosomal organs of scorpions (Chelicerata, Scorpiones), with implications for the phylogeny of the order. Zoological Journal of the Linnean Society 154:651–675.

[42] González-Santillan E, Prendini L (2013) Redefinition and generic revision of the North American vaejvoid scorpion subfamily Syntropinae Kraepelin, 1905, with descriptions of six new genera. Bulletin of the American Museum of Natural History 382:1–71.

[43] Monod L, Prendini L (2014). Evidence for Eurogondwana: The roles of dispersal, extinction and vicariance in the evolution and biogeography of Indo-Pacific Hormuridae (Scorpiones: Scorpionoidea). Cladistics doi:10.1111/cia.12067. 10.1111/cla.12067 34758586

[44] Fet V, Soleglad ME, Kovařík F (2009) Etudes on iurids, II. Revision of genus *Calchas* Birula, 1899, with the description of two new species (Scorpiones: Iuridae). Euscorpius 82:1–72.

[45] Kovařík F, Fet V, Soleglad ME, Yağmur EA (2010) Etudes on iurids, III. Revision of the genus *Iurus* Thorell, 1876 (Scorpiones, Iuridae), with a description of two new species from Turkey. Euscorpius 95:1–214.

[46] Soleglad ME, Fet V, Kovařík F, Yağmur EA (2012) Etudes on iurids, V. Further revision of *Iurus* Thorell, 1876 (Scorpiones: Iuridae), with a description of a new genus and two new species. Euscorpius 143:1–72.

[47] González-Sponga MA (1996) Guía para identificar escorpiones de Venezuela. Caracas: Cuadernos Lagoven, 204 pp.

[48] Botero-Trujillo R, Francke OF (2009) A new species of troglomorphic leaf litter scorpion from Colombia belonging to the genus *Troglotayosicus* (Scorpiones: Troglotayosicidae). Texas Memorial Museum Speleological Monographs 7:1–10.

[49] Ochoa JA, Botero-Trujillo R, Prendini L (2010) On the troglomorphic scorpion *Troglotayosicus humiculum* (Scorpiones, Troglotayosicidae), with first description of the adults. American Museum Novitates: 1–19.

[50] Botero-Trujillo R, Ochoa JA, Tovar OA, Souza JE (2012) A new species in the scorpion genus *Troglotayosicus* from forest leaf litter in southwestern Colombia (Scorpiones, Troglotayosicidae). Zootaxa 3506:63–76.

