# Supplementary material for: Homology of the Lateral Eyes of Scorpiones: A Six-Ocellus Model
Source: PLoS One. 2014 Dec 3;9(12):e112913. doi: 10.1371/journal.pone.0112913 (PMC4254604; doi:10.1371/journal.pone.0112913)
Supplement: Appendix S1 — Lateral ocelli of Recent scorpion families: Material examined. Abbreviations for collections as follows: American Museum of Natural History, New York, U.S.A. (AMNH); Natural History Museum, London, U.K. (BMNH); California Academy of Sciences, San Francisco, U.S.A. (CAS); Field Museum of Natural History, Chicago, IL, U.S.A. (FMNH); Hebrew University of Jerusalem, Israel (HUJ); Instituto de Biología, Universidad Nacional Autónoma de México, México City (IBUNAM); Museum of Comparative of Zoology, Harvard University, Cambridge, MA, U.S.A. (MCZ); Muséum National d'Histoire Naturelle, Paris, France (MNHN); Western Australian Museum, Perth, Australia (WAM); W. David Sissom Private Collection, Canyon, TX, U.S.A. (WDS). (DOC) [file pone.0112913.s001.doc]

**Appendix S1. Lateral ocelli of Recent scorpion families: Material examined.** Abbreviations for collections as follows: American Museum of Natural History, New York, U.S.A. (AMNH); Natural History Museum, London, U.K. (BMNH); California Academy of Sciences, San Francisco, U.S.A. (CAS); Field Museum of Natural History, Chicago, IL, U.S.A. (FMNH); Hebrew University of Jerusalem, Israel (HUJ); Instituto de Biología, Universidad Nacional Autónoma de México, México City (IBUNAM); Museum of Comparative of Zoology, Harvard University, Cambridge, MA, U.S.A. (MCZ); Muséum National d’Histoire Naturelle, Paris, France (MNHN); Western Australian Museum, Perth, Australia (WAM); W. David Sissom Private Collection, Canyon, TX, U.S.A. (WDS).

**Family Akravidae Levy, 2007**

*Akrav israchanani* Levy, 2007: **ISRAEL:** Ayyalon Cave, southern coastal plain, iv.2006, I. Na’aman, holotype (HUJ 2673), 6 paratypes (HUJ 2674–2679).

**Family Bothriuridae Simon, 1880**

*Bothriurus bonariensis* (C.L. Koch, 1842): **ARGENTINA:** Provincia de Entre Río: El Palmer National Park, road to Arroyo el Palmer, 31°53'54.636"S 58°14'38.328"W, 32 m, 14.xii.2005, C.I. Mattoni, A.A. Ojanguren & F. Labarque, 3 ♂, 2 ♀ (AMNH).

*Bothriurus vittatus* (Guérin Méneville, 1838): **CHILE:** Región IX (Araucanía): Las Quilmas campsite and surrounds, El Manzano (between Vegas Blancas and Angol), 37°48'16.560"S 72°52'17.940"W, 599 m, 16.i.2006, C.I. Mattoni, M. & F. Vivanco, 1 ♀, 2 juv. (AMNH [LP 5649]).

*Brachistosternus multidentatus* Maury, 1984: **ARGENTINA:** Provincia de Mendoza: Reserva Natural Bosque Telteca, 32°22.967'S 68°03.309'W, 580 m, 19.xi.2003; L. Prendini, C.I. Mattoni & J.A. Ochoa, 2 juv. (AMNH [LP 2457]).

*Brandbergia haringtoni* Prendini, 2003: **NAMIBIA:** Erongo Region: Omaruru District: Brandberg, Goaseb, 21°14'S 14°35'E, 1650 m, 1.iii.1978, H. Pager, holotype ♀, paratype ♀ (AMNH [AH 1029]).

*Centromachetes* sp.: **CHILE:** Región VIII (Bío Bío): 9 km E of Antiguala, 6 km E of Caramavida bridge, 37°43'5.268"S 73°18'46.548"W, 12.i.2006, 105 m, C.I. Mattoni, 2 ♂ (AMNH).

*Cercophonius sulcatus* Kraepelin, 1908: **AUSTRALIA:** Western Australia: Perth scarp, 31°35'14.2"S 116°12'14.2"E, 17.v.1999, E.S. Volschenk & J. Warden, 1 ♀ (AMNH [LP 1613]).

*Lisposoma elegans* Lawrence, 1928: **NAMIBIA:** Erongo Region: Swakopmund District: Namib-Naukluft Park: Gobabeb, pitfall site on gravel plains ca. 700 m N, 23°32'54.4"S 15°02'49.5"E, 395 m, 27.iii.2006, L. Prendini, T.L. Bird & S.K. Uunona, 2 ♂ (AMNH); Gobabeb, pitfall site on gravel plains ca. 1 km N, 23°32'39.6"S 15°02'57.4", 422 m, 27.iii.2006, L. Prendini, T.L. Bird & S.K. Uunona, 2 ♂ (AMNH); Swakop River Campsite, 5 km S, between turnoff to Welwitschiavlakte and Swakop riverbed, 22°44'53.6"S 14°57'11.9"E, 355 m, 30.iii.2006, L. Prendini, T.L. Bird & S.K. Uunona, 1 ♀ (AMNH).

*Lisposoma josehermana* Lamoral, 1979: **NAMIBIA:** Otjozondjupa Region: Otjiwarongo District: Waterberg Plateau Park, Main Camp, 20°30.803'S 17°14.753'E, 1440 m, 1.i.2004, L. Prendini, E. Scott, Q. & N. Martins, 1 ♂ (AMNH).

*Orobothriurus paessleri* (Kraepelin, 1911): **PERU:** Departamento de Arequipa: Provincia de Islay: Mejma, Lomas de Challascapa, 3.iii.2004, R. Gutierrez & J.A. Ochoa, 1 subad. ♂ (AMNH [LP 3057]).

*Pachakutej inca* (Maury, 1975): **PERU:** Departamento de Cajamarca: Provincia de Hualgayoc: Capulí, Bambamarca, 18.xi.2004, J.C. Chaparro & J.A. Ochoa, 1 juv. ♀ (AMNH [LP 6259B]).

*Phoniocercus sanmartini* Cekalovic, 1968: **CHILE:** Provincia de Osorno: Pucatrihue, 10–24.ii.1985, L.E. Peña, 5 ♂ (AMNH).

*Rumikiru atacama* Ojanguren-Affilastro et al., 2012: **CHILE:** Región III (Atacama): Provincia de Huasco: Llanos de Challe National Park, 7.5 km from administration building, 28°07.639'S 71°05.090'W, 88 m, 10.xi.2003, L. Prendini, C.I. Mattoni & J.A. Ochoa, 3 ♂, 3 ♀ paratypes (AMNH).

*Thestylus glasioui* Bertkau, 1880: **BRAZIL:** Rio de Janeiro: Município do Teresópolis: Serra dos Órgãos National Park, trilha do Sino, campos de altitude, 22°27'18.930"S 43°01'15.540"W, 1908 m, 10.i.2007, C.I. Mattoni, R. Pinto-da-Rocha & S. Outeda-Jorge, 1 ♀ (AMNH).

*Timogenes mapuche* Maury, 1975: **ARGENTINA:** Provincia de Neuquén: Picún Leufú, 39°32'13.5''S 69°13'21.8''W, 399 m, 25.i.2005, M. Magnanelli & E.G. López, 1 ♂ (AMNH [LP 4312]). Provincia de Río Negro: Paso Córdova, “Valle de la Luna” trail, 39°07'39.2''S 67°40'36.7''W, 289 m, 18.i.2006, C.I. Mattoni & M. Vivanco, 1 ♂ (AMNH [LP 5653]).

*Urophonius granulatus* Pocock, 1898: **ARGENTINA:** Provincia de Santa Cruz: Los Antiguos, 46°34'18.3"S 71°38'44.8"W, 324 m, 19.i.2005, M. Magnanelli & E.G. López, 6 ♀ (AMNH).

*Vachonia martinezi* Abalos, 1954: **ARGENTINA:** Balneario El Condor, 5.v.2003, M. Magnanelli, 1 juv. ♀ (AMNH [LP 2441]).

**Family Buthidae C.L. Koch, 1837**

*Afroisometrus minshullae* (FitzPatrick, 1994): **BOTSWANA:** North East District: Selkirk Mine, near Francistown, 21°19.332'S 27°42.372'E, 30.x–6.xi.2007, D.H. Jacobs, 1 ♀ (AMNH [LP 7875]).

*Akentrobuthus atakora* Vignoli & Prendini, 2008: **BENIN:** Cascade de Tanogou, Tanogou, 10°48.12'N 01°26.26'E, 261 m, 9.vi.2005, V. Vignoli & S. Tchibozo, holotype ♀ (AMNH [LP 8334]).

*Alayotityus nanus* Armas, 1973: **CUBA:** Santiago de Cuba Province: Quintero, Santiago de Cuba, 15.iv.1999, R. Teruel, 1 ♂ (AMNH [LP 1768]).

*Ananteris inoae* González-Sponga, 2006: **VENEZUELA:** Aragua: Município de Girardot: Parque Nacional Henri Pittier, Altos de Choroni, road Maracay–Puerto Colombia, 10°22.300'N 67°35.511'W, 1200 m, to 10°21.683'N 67°35.032'W, 1582 m, 1.viii.2009, F. Rojas-Runjaic, A. Ferrer, L. Prendini & J.A. Ochoa, 1 ♀ (AMNH).

*Androctonus finitimus* (Pocock, 1897): **PAKISTAN:** Punjab: Jhang District: Shorkot Tehsil: Kashmir Sugar Mills, 5 km from Shorkot Bhudh, 30°45.677'N 72°41.372'E, 438 ft, 23.v.2012, M. Irfan & T. Abbas, 3 ♂, 1 subad. ♂, 1 juv. ♂ (AMNH).

*Anomalobuthus rickmersi* Kraepelin, 1900: **UZBEKISTAN:** Navoiy Area: Kanimekh District: 18 km W of Chengeldy village, ca. 1–1.5 km W of Darbazatepa hill, 40°57.178'N 64°08.169'E, 220 m, 4.vi.2003, L. Prendini & A.V. Gromov, 1 ♀, 1 subad. ♂, 1 juv. ♂ (AMNH).

*Apistobuthus* *pterygocercus* Finnegan, 1932: **UNITED ARAB EMIRATES:** ix.2000, 1 ♀ (AMNH [LP 1795]).

*Australobuthus xerolimniorum* Locket, 1990: **AUSTRALIA:** South Australia: Lake Hart, 34°12'40.5"S 140°37'42.4"E, 9.xii.1998, E.S. Volschenk & J. Tregear, 1 ♂ (AMNH [LP 1666]).

*Babycurus gigas* Kraepelin, 1896: **TANZANIA**: Amani, East Usambara Mt., ix.2006, J. Beraducci, 2 ♂ (AMNH).

*Birulatus haasi* Vachon, 1974: **JORDAN:** Ma'an Governate: Al Shaubak (Showbak/Shawbak) castle, slopes below, 30°31'56.9"N 35°33'36.1"E, 1333 m, 8.ix.2013, L. Prendini, Z. Amr, O. Abed, T. Al Share & L. Al Azam, 1 ♂ (AMNH [LP 12162]).

*Buthacus macrocentrus* (Ehrenberg, 1828): **TURKEY:** Sanliurfa Province: Birecik, ca. 20 km E, 37°00'38.4"N 38°11'41.1"E, 686 m, 23.v.2007, A.V. Gromov & E.A. Yağmur, 2 subad. ♀, 1 juv. ♂ (AMNH).

*Butheoloides milloti* Vachon, 1948: **SENEGAL:** Kedougou, 3 km W along road to Salemata, 12°33'10.6"N 12°13'39.4"W, 3.vii.2005, 126 m, J. Huff & V. Vignoli, 1 ♀ (AMNH [LP 4505]).

*Butheoloides monodi* Vachon, 1950: **GUINEA-BISSAU:** Bambadinca, Riverzoo Farm, 12°00'09.0"N 14°53'25.9"W, 29.vi–2.vii.2005, 28 m, J. Huff & V. Vignoli, 1 ♀ (AMNH [LP 4510]. **SENEGAL:** Koungheul, 20 km W, near Mbaye Mbaye, 13°58'21.4"N 15°00'28.7"W, 55 m, 27.vi.2005, J. Huff & V. Vignoli, 1 ♀ (AMNH [4507]).

*Butheolus gallagheri* Vachon, 1980: **OMAN:** iv.2003, 1 ♀ (AMNH [LP 2279]), 1 ♂ (AMNH [LP 2281]).

*Buthiscus bicalcaratus* Birula, 1905: **ALGERIA:** NE of Timimoun, 1 ♀ (MNHN [RS 1715]).

*Buthoscorpio rayalensis* Javed et al., 2010: **INDIA:** Andhra Pradesh: Nandyal, 15 km E, 19.ix.2001, S. Basi, 1 ♀ (AMNH [LP 1915]).

*Buthoscorpio sarasinorum* (Karsch, 1891): **SRI LANKA:** Laggala: Matale–Rattuta road N of Knuckles range, 07°32'74"N 80°47'82"E, 423 m, 17.iii.1999, M. Vences, 1 ♀ (AMNH [LP 1507]).

*Buthus malhommei* Vachon, 1949: **MOROCCO:** Marrakech, 22 km N, 31°48'03.3"N 07°58'48.5"W, 525 m, 24.ix.2004, V. Vignoli, 3 ♂ (AMNH).

*Centruroides vittatus* (Say, 1821): **U.S.A.:** Texas: Brewster County: Big Bend National Park, Juniper Flat (Basin Area), 5900 ft, 27.v.1965, K.W. Haller, 1 ♂, 1 ♀ (AMNH).

*Charmus minor* Lourenço, 2002: **SRI LANKA:** North Western Province: Puttalam District: Wilpattu National Park, Kokmotte Campsite on Modaragam Aru River, 08°31'41.9"N 80°01'25.1"E, 30 m, 24–25.i.2014, L. Prendini & P. Horsley, 1 ♂ (AMNH [LP 12320]).

*Cicileus exilis* (Pallary, 1928): **ALGERIA:** Initinen (Tassili n'Ajjer), 24.i.1963, H. Lhote; Sefar (Tassili), 4.i.1967, J. Garzoni, 2 ♀, 2 subad. ♀, 1 juv. ♀ (MNHN [RS 3948]).

*Compsobuthus levyi* Kovařík, 2012: **ISRAEL:** Southern District: HaRo'a campsite, near Midreshet Ben-Gurion (Ben-Gurion University Sede Boqer campus), 30°52'29.6"N 34°47'09.7"E, 466 m, 3–4.ix.2011, L. Prendini, T.L. Bird, E. Gefen, S. Huber et al., 3 ♂, 1 ♀ (AMNH).

*Grosphus ankarana* Lourenço & Goodman, 2003: **MADAGASCAR:** Antsiranana Province: Réserve Spéciale d’Ankarana, 2.6 km E of Andrafiabe, 12°57.523'S 49°07.189'E, 4–28.i.2007, A.H. Kirk-Spriggs, 5 ♀ (AMNH).

*Hemilychas alexandrinus* (Hirst, 1911): **AUSTRALIA:** Queensland: Culgoa Flood Plain National Park, Byra section, 28°54'10"S 147°08'42"E, 11–18.xi.1998, C. Eddie & S. Rankin, 1 ♀ (AMNH [LP 1671]).

*Hottentotta arenaceus* (Purcell, 1901): **SOUTH AFRICA:** Northern Cape Province: Calvinia District: Farm Platklip 328, Kromrivier intersection with R355 (Kliprand–Loeriesfontein), 11.3 km NW of turnoff to Lospersplaas, 30°50.694'S 19°07.797'E, 454 m, 26.ii.2009, L. Prendini & H. Bichard, 1 ♂, 1 ♀ (AMNH).

*Isometroides* sp.: **AUSTRALIA:** South Australia: Coolton National Park, 34°12'40.5"S 140°37'42.4"E, 9.xii.1998, E.S. Volschenk & J. Tregear, 1 ♀ (AMNH [LP 1656]).

*Isometrus maculatus* (DeGeer, 1778): **SÃO TOME AND PRÍNCIPE:** São Tomé: Praia du Mutamba, 00°23'45.5''S 06°36'19.1''E, 2.iv.2001, J.M. Ledford, 1 ♀ (AMNH [LP 1788]). **SRI LANKA:** Wellawaya, 24.ii.2000, D. Huber, 1 ♂, 1 ♀ (AMNH [LP 1798]).

*Isometrus petrzelkai* Kovařík, 2003: **VIETNAM:** Bin Thuan Province: Ham Thuan Nam District: Ta Kou Mountain Nature Reserve: Ta Kou Mountain (Nui Ta Kou), trail below guesthouse and upper cable station, 10°48'45.1"N 107°53'30.7"E, 383 m, 22–23.vi.2012, L. Prendini & S.F. Loria, 1 ♂, 1 subad. ♂ (AMNH).

*Karasbergia methueni* Hewitt, 1913: **NAMIBIA:** Karas Region: Bethanie District: Farm Dabis 15, Konkiep River, 11.8 km N of D414 turnoff to Gibeon on C14 (Maltahöhe–Helmeringhausen), 12.6 km N of Helmeringhausen, 25°47'05.4"S 16°50'58.8"E, 1363 m, 6.ii.2008, L. Prendini & T.L. Bird, 1 ♂ (AMNH [LP 8226]). Karasburg District: Ai-Ais and Fish River Canyon National Park, Farm Rosyntjiebos 375, 13.5 km N of intersection with C10 (Ai-Ais–Grünau) on C37 to Hobas/Fish River Canyon, 27°48'59.5"S 17°40'24.6"E, 687 m, 5.ii.2008, L. Prendini & T.L. Bird, 1 ♂ (AMNH [LP 8225]). **SOUTH AFRICA:** Northern Cape Province: Richtersveld National Park, Potjiespram, ii.1997, L. Prendini & E. Scott, 1 ♂ (AMNH [LP 1725]).

*Kraepelinia palpator* (Birula, 1903): **IRAN:** Kerman Province: 30°07'25.6''N 57°11'26.7''E, v.2009, Koohpaye, Jamalizadeh & Ebrahimi, 1 ♂ (AMNH [LP 11064]).

*Leiurus hebraeus* (Birula, 1908): **ISRAEL:** Southern District: Midreshet Ben-Gurion (Ben Gurion University Sede Boqer campus), 1 km W at intersection road 40 and turnoff to Ben Gurion College, 30°51'20.4"N 34°46'11.5"E, 490 m, 5.ix.2011, L. Prendini, E. Gefen et al., 1 ♂, 1 ♀, 1 subad. ♀ (AMNH).

*Liobuthus kessleri* Birula, 1898: **UZBEKISTAN:** Khorezm Area: Hazorasp District: Kyzylkum Desert, Uchizhak Hills, ca. 19 km N of Turpakkala, ca. 10 km E of Lebap [Turkmenistan], 41°01.673'N 62°00.361"E, 227 m, 31.v.2003, L. Prendini & A.V. Gromov, 1 ♂, 1 ♀, 1 juv. ♂ (AMNH).

*Lissothus bernardi* Vachon, 1948: **ALGERIA:** 1960, F. Bernard, 1 ♀ (MNHN [RS 3420]).

*Lychas burdoi* (Simon, 1882): **SOUTH AFRICA:** Limpopo Province: Phalaborwa District: Kruger National Park, Shingwedzi Research Camp, 23°06'59.9"S 31°25'48.8"E, 273 m, 5.i.2007, Spider Club of Southern Africa, 2 ♂, 4 ♀ (AMNH).

*Lychas scutilus* C.L. Koch, 1845: **SINGAPORE:** Bukit Timah Nature Reserve, Bukit Timah, 01°21'16.85"N 103°46'34.95"E, 163 m, 3.vi.2014, L. Prendini & S.F. Loria, 1 ♂, 1 subad. ♀ (AMNH).

*Mesobuthus caucasicus* (Nordmann, 1840): **KAZAKHSTAN:** Almaty Area: Balkhash District: Saryesik-Atyrau Desert, 25 km SE of Bakanas, E bank of Ili River, 44°39.247'N 76°31.348'E, 403 m, 8.v.2003, L. Prendini & A.V. Gromov, 2 ♀ (AMNH).

*Mesotityus vondangeli* González-Sponga, 1981: **VENEZUELA:** Aragua: Costa de Oro: Parque Nacional Henri Pittier: Caserio La Trilla, 10°23.804'N 67°45.220'W, 90 m, 10.vii.2009, F. Rojas-Runjaic, A. Ferrer & J.A. Ochoa, 1 ♀ (AMNH [LP 10062]); Cumboto, Chopo Almao, 10°23.866'N 67°47.686'W, 89 m, 11.vii.2009, F. Rojas-Runjaic, A. Ferrer & J.A. Ochoa, 1 ♂ (AMNH [LP 10061]).

*Microananteris* sp.: **BRAZIL:** Amazonas: Atalaia do Norte: Palmari Natural Reserve, 31 km W of Benjamin Constant, ix.2009, 1 ♂ (AMNH [LP 10666]).

*Microbuthus maroccanus* Lourenço, 2002: **MOROCCO:** Tan Tan Plage, 500 m N, 28°30.13.3'N 11°19.03.6'W, 42 m, 7.ix.2004, V. Vignoli, 1 ♀ (AMNH [LP 3575]).

*Microcharmus pauliani namoroka* Lourenço et al., 2006: **MADAGASCAR:** Mahajanga Province: Parc National de Namoroka, 9.8 km 300° WNW of Vilanandro, 16°28'00''S 45°21'00''E, 140 m, 4–8.xi.2002, B.L. Fisher, C.E. Griswold et al., 1 ♂, 1 ♀ (AMNH [LP 2821]).

*Microtityus consuelo* Armas & Marcano Fondeur, 1987: **DOMINICAN REPUBLIC:** La Altagracia Province: Parque Nacional del Este, track between ranger station at Bayahibe and La Tortuga, 18.3281°N 68.8028°W, 8.54 m, 13.vii.2004, E.S. Volschenk & J. Huff, 2 ♀ (AMNH [LP 3281]).

*Neobuthus awashensis* Kovařík & Lowe, 2012: **ETHIOPIA:** Afar Region: Metahara, 09°00.987'N 39°51.273'E, 1052 m, 28.v.2011, V. Hula & J. Niedobová, 1 subad. 1 ♀ (AMNH [LP 11225]).

*Neogrosphus griveaudi* (Vachon, 1969): **MADAGASCAR:** Toliara Province: Atsimo-Andrefana Region: Betioky District: Commune Rurale Beheloka: Beheloka, 11.8 km SE, 24°01'35.8"S 43°44'13.5"E, 19 m, 12.x.2011, 2 ♂, 1 ♀ (FMNH [VS 1986]).

*Odontobuthus odonturus* (Pocock, 1897): **PAKISTAN:** Punjab: Sargodha District: Noshara Tehsil: Nory Wala, 6 km NW of Khaliq Abad towards Noshra, near Police Station, 32°26.252'N 73°32.139'E, 819 ft, 19.vi.2012, M. Tahir, S.Y. Khan, M. Ashan & M. Jafer, 2 ♂, 1 ♀ (AMNH).

*Odonturus dentatus* Karsch, 1879: **TANZANIA:** Kilwa District: Mbarawala Plateaux, 09°02.374'S 39°07.206'E, 29.ii–4.iii.2008, P.G. Hawkes, 3 ♂, 3 ♀ (AMNH).

*Orthochirus scrobiculosus* (Grube, 1873): **UZBEKISTAN:** Surkhandarya Area: Angor District: Kattakum Desert, 4.5 km NE of Uchyzyl, 37°22.549'N 67°16.618'E, 331 m, 19.v.2003 L. Prendini & A.V. Gromov, 1 ♂, 1 ♀ (AMNH).

*Parabuthus transvaalicus* Purcell, 1899: **SOUTH AFRICA:** Limpopo Province: Soutpansberg District: Farm Gansvley 335, 15 km S of Alldays intersection of Waterhout and Withaak streets, 22°48'37.2"S 29°05'21.5"E, 802 m, 22.ii.2011, L. Prendini & P. Gildenhuys, 1 ♂, 1 ♀ (AMNH).

*Physoctonus debilis* (C.L. Koch, 1840): **BRAZIL:** Pernambuco: Exu, 5 km N, 4.x.1977, L.J. Vitt, 1 ♀ (AMNH).

*Pseudolychas ochraceus* (Hirst, 1911): **SOUTH AFRICA:** Gauteng Province: Irene, i.2005, J. du G. Harrison, 3 ♀ (AMNH).

*Pseudouroplectes betschi* Lourenço, 1995: **MADAGASCAR:** Toliara Province: Parc National de Tsimanampetsotsa, 6.7 km 130° SE of Efoetse, 23.0 km 175° S of Beheloka, 24°06'02''S 43°45'36''E, 25 m, 18–22.iii.2002, B.L. Fisher, C.E. Griswold et al., 1 ♂ (AMNH [LP 2818]); Réserve Spéciale de Cap Sainte Marie, 14.9 km 261° W of Marovato, 25°35'40''S 45°08'49''E, 160 m, 13–19.ii.2002, B.L. Fisher, C.E. Griswold et al., 1 ♂ (AMNH [LP 2816]).

*Razianus zarudnyi* (Birula, 1903): **IRAQ:** Bazair, 16.iv.1934, E.W. Kaiser, 1 ♀ (AMNH).

*Rhopalurus virkkii* Santiago-Blay, 2009: **U.S.A.:** Puerto Rico: Isla Mona, road to El Faro, 18.064°N 67.869°W to 18.085°N 67.848°W to 18.096°N 67.936°W, 17.x.2009, L.A. Esposito & H. Yamaguti, 2 ♂, 3 ♀, 1 subad. ♂, 1 juv. ♂ (AMNH).

*Sassanidotus gracilis* (Birula, 1900): **IRAN:** Kerman Province: 27°47'18.6''N 58°35'7.7''E, iv.2009, Koohpaye, Jamalizadeh & Ebrahimi, 2 ♂ (AMNH [LP 11073]).

*Thaicharmus mahunkai* Kovařík, 1995: **THAILAND:** Phetchaburi Province: Kaeng Krachan District: Kaeng Krachan National Park, 12°52'58.5''N 99°37'46.6''E, 100 m, 24.iv.2014, S.F. Loria & P. Horsley, 1 ♀ (AMNH).

*Tityobuthus petrae* Lourenço, 1996: **MADAGASCAR:** Fianarantsoa Province: Forêt d’Analalava, 29.6 km 280° W of Ranohira, 22°35'30''S 45°07'42''E, 700 m, 1–5.ii.2003, B.L. Fisher, C.E. Griswold et al., 1 ♂, 1♀, 1 juv. (AMNH [LP 2834]).

*Tityopsis inexpectata* (Moreno, 1940): **CUBA:** Rio Margen, San Antonio, San Antonio de los Baños, Habana, vii.1979, L.F. Armas, 1 ♀ (AMNH).

*Tityus bahiensis eickstedtae* Lourenço, 1982: **BRAZIL:** viii.1999, ex R. Pinto-da-Rocha, 1 ♂, 1 ♀, 1 juv. (AMNH [LP 1596]).

*Uroplectes carinatus* (Pocock, 1890): **SOUTH AFRICA:** Limpopo Province: Potgietersrus District, Farm Julietta 112, ca. 63 km NE of Ellisras, 23.264°S 28.159°E, 4.xii.2010, 935 m, P.G. Hawkes & J.N. Fisher, 6 ♂, 1 ♀ (AMNH).

*Vachoniolus globimanus* Levy et al., 1973: **UNITED ARAB EMIRATES:** Rub' Al Khali Desert, ca. 17 km SE of Dubai, 25°10'04''N 55°28'07''E, 60 m, 28.iii.2003, A.V. Gromov, 1 ♂, 1 ♀ (AMNH [LP 3767]).

*Zabius fuscus* (Thorell, 1876): **ARGENTINA:** Provincia de Córdoba: Capilla de Olaen, ca. 11 km W of Molinari, 31°09'44.46"S 64°36'24.336"W, 1096 m, 29.xii.2005, C.I. Mattoni, A. Peretti, P. Carreras, M. Zerda & D. Vrech, 1 ♀, 1 subad. ♀ (AMNH).

**Family Chactidae Pocock, 1893**

*Broteochactas nitidus* Pocock, 1893: **TRINIDAD & TOBAGO:** Trinidad: Mt. St. Benedict, 10°39'49"N 61°23'56"W, 28.vi.1999, L. Prendini, 5 ♀, 1 subad. ♂ (AMNH [LP 1511]).

*Brotheas granulatus* Simon, 1877: **FRENCH GUIANA:** Approuague-Kaw Canton: Kaw Mountains, end of Kaw road to boat ramp, trail on left, 04°30'22.6"N 52°03'29.9"W, 20–120 m, 24.xii.2004, J. Huff, 2 ♂, 1 ♀ (AMNH [LP 3656]). Roura District: Tresor Mountains, 23.ix.2004, R.C. West, 1 juv. ♀ (AMNH [LP 3445B]).

*Brotheas wareipai* González-Sponga, 2004: **VENEZUELA:** Bolivar: Município de Gran Sabana: Parque Nacional Canaima, Campamento Uruyen, near lodge, 05°40.981'N 62°27.205'W, 511 m, 30.vii.2009, L. Prendini & J.A. Ochoa, 1 ♂, 4 ♀, 2 subad. ♀ (AMNH).

*Chactas raymondhansi* Francke & Boos, 1986: **TRINIDAD & TOBAGO:** Trinidad: Mt. El Tucuche (summit), 8.vii.1999, L. Prendini, 1 ♂, 1 ♀, 1 subad. ♀ (AMNH [LP 1586]).

*Chactopsis insignis* Kraepelin, 1912: **PERU:** Región Loreto: Provincia de Maynas: Distrito de Fernando Lores: Comunidad Diamante, Quebrada Blanco, 04º22.268'S 73º09.708'W, 113 m, 26.ii.2008 C. Gil, E. Guerra & J.A. Ochoa, 1 ♀ (AMNH [LP 8420]).

*Chactopsoides anduzei* (González-Sponga, 1982): **VENEZUELA:** Amazonas: Município de Atures: between Gavilan and Las Pavas, E of Puerto Ayacucho, 05°32.308'N 67°24.676'W, 89 m, 11.x.2008, J.A. Ochoa & S. Bazo, 1 ♀ (AMNH [LP 9239]); Puerto Ayacucho, 05°38.760'N 67°34.778'W, 85–100 m, 10.x.2008, J.A. Ochoa & S. Bazo, 1 ♀ (AMNH [LP 9215]); Puerto Ayacucho, road to Tobogan de la Selva, 05°36.712'N 67°35.717'W, 76 m, 15.viii.2009, F. Rojas-Runjaic & J.A. Ochoa, 1 juv. (AMNH [LP 10089]). Município de Autana: Isla Ratón (S part), Río Orinoco, 05°03.882'N 67°49.000'W, 81 m, 7.viii.2009, F. Rojas-Runjaic, A. Ferrer & J.A. Ochoa, 1 ♂, 1 ♀ (AMNH), 1 juv. (AMNH [LP 10087]).

*Hadrurochactas machadoi* González-Sponga, 1993: **VENEZUELA:** Bolivar: Município de Gran Sabana: Parque Nacional Canaima, SW of Uruyen, 05°40.803'N 62°28.705'W, 504 m, 28.vii.2009, L. Prendini & J.A. Ochoa, 1 ♀, 2 subad. ♀ (AMNH).

*Hadrurochactas* sp.: **BRAZIL:** Roraima: Vila Tepequém, Amajari, 03°46'07"N 61°44'33"W, 11.xi.2008, H. Yamaguti & R. Pinto-da-Rocha, 1 ♀ (AMNH [LP 9651]).

*Megachactops kuemoi* Ochoa et al., 2013: **VENEZUELA:** Amazonas: Município de Autana: base of Autana, 04°48.173'N 67°29.171'W, 84–90 m, 9.x.2008, J.A. Ochoa & S. Bazo, 1 subad. ♀ paratype (AMNH [LP 9230]), 1 juv. paratype (AMNH [LP 9244]).

*Neochactas delicatus* (Karsch, 1879): **FRENCH GUIANA:** Approuague-Kaw Canton: Kaw mountains, area around Amazone Nature Lodge, 04°33'35.0"N 52°12'25.3"W, 290 m, 21–25.xii.2004, J. Huff, 1 ♀ (AMNH [LP 3658]). Roura District: Tresor Mountains, 23.ix.2004, R.C. West, 1 ♀ (AMNH [LP 3441]).

*Nullibrotheas allenii* (Wood, 1863): **MEXICO:** Baja California Sur: El Crucero, 51 mi. N, 400 ft, 14.v.1969, S.C. Williams, 3 ♀, 1 subad. ♂, 1 juv. ♂, 4 juv. ♀ (CAS).

*Taurepania porosus* (Pocock, 1900) **VENEZUELA:** Bolivar: Mt. Roraima, summit, 9100 ft, 21.x.2005, R.C. West, 1 ♀ (AMNH [LP 5513]).

*Taurepania vestigialis* González-Sponga, 1978: **VENEZUELA:** Bolivar: Mt. Roraima, base, 6500 ft, 19.x.2005, R.C. West, 1 ♀ (AMNH [LP 5512]).

*Teuthraustes glaber* Kraepelin, 1912: **PERU:** Departamento de San Martín: San Martín, 890 M.A.S. Ekin, E of Tarapoto, 9–21.iii.1947, F. Woytkowski, 2 ♀ (AMNH).

*Vachoniochactas humboldti* Florez et al., 2008: **VENEZUELA:** Amazonas: Município de Autana: Autana River mouth, 04°44.795'N 67°41.682'W, 82 m, 8.x.2008, J.A. Ochoa & S. Bazo, 1 subad. ♀ (AMNH [LP 9266]).

*Vachoniochactas lasallei* (González-Sponga, 1978): **VENEZUELA:** Bolivar: Município de Sifontes: La Escalera, between Piedra de la Virgen and Monumento Soldado Pionero Pionero (km 127 road El Dorado–Santa Elena de Uairén), 05°55.067'N 61°26.227'W, 1398 m, 16.vii.2009, A. Yepez, M. Blanco & J.A. Ochoa, 1 subad. ♂ (AMNH [LP 10000]).

**Family Chaerilidae Pocock, 1893**

*Chaerilus chapmani* Vachon & Lourenço, 1985: **MALAYSIA:** Sarawak: Gunung Mulu National Park, Clearwater III Cave, Gunung Api, v.1984, 1 ♀ (AMNH).

*Chaerilus julietteae* Lourenço, 2011: **VIETNAM:** Bin Thuan Province: Ham Thuan Nam District: Ta Kou Mountain Nature Reserve, Ta Kou Mountain (Nui Ta Kou), trail above pagoda, rocky ravine on opposite side of summit, 10°49'01.9"N 107°53'49.3"E, 23.vi.2012, 592 m, L. Prendini & S.F. Loria, 2 ♂, 3 ♀ (AMNH).

*Chaerilus telnovi* Lourenço, 2009: **INDONESIA:** Maluka Utara Province: Halmahera Tengah: Weda Seltan District: Loleo village S env., Tilope village, 10–15 km SW between Gunung Talaga and Cham, 50 m, 12.ix.2007, D. Telnov & K. Greke, holotype ♀ (MNHN).

*Chaerilus variegatus* Simon, 1877: **INDONESIA:** Banten Province: Pandeglang: Ujung Kulon National Park, Kertamukti, Honja Mountain, 74 m, 06°42.55.8''S 105°33.47.5''E, Loria, Sarino & Oman 1 ♂, 6 ♀, 1 subad. ♂ (AMNH), 1 juv. ♂ (AMNH [LP 12105]). North Sumatra Province: Lumban Rang National Park, near road Prapat to Porsea, 15 km from Prapat, 02°36'14''N 99°02'42''E, 1350 m, 1.vii.2006, P. Schwendinger, 1 ♂ (AMNH [LP 6389]); Kampus Kehutanan Aeknauli, 1.5 km SE, near Pematangsiantar, 13 km from Prapat, 02°42'38''N 98°56'16''E, 1150 m, 30.vi–2.vii.2006, P. Schwendinger, 1 ♂ (AMNH [LP 6390]).

**Family Diplocentridae Karsch, 1880**

*Bioculus caboensis* (Stahnke, 1968): **MEXICO:** Baja California Sur: Município de Los Cabos: Sierra de la Laguna, 23°14'17.1"N 109°57'7.9"W, 782 m, 10.vii.2004, E. González, O.F. Francke, W.E. Savary & A. Valdez, 1 juv. ♀ (AMNH).

*Bioculus cruzensis* Stahnke, 1968: **MEXICO**: Baja California Sur: Município de La Paz: Isla Santa Cruz, southern area, 25°15.697'N 110°43.669'W, 11.vii.2008, H. Montaño & E. González, 0–50 m, 1 ♂, 1 subad. ♀ (AMNH).

*Cazierius neibae* Kovařík & Teruel, 2014: **DOMINICAN REPUBLIC:** Provincia de la Independencia: Sierra de Neyba: Parque Nacional Sierra de Neyba: Los Bolos, ca. 2 km S, N of Guayabal, 18°36.910'N 71°38.592'W, 769 m, 17.vii.2010, J. Huff & A. Sanchez, 1 ♂, 1 ♀ (AMNH).

*Didymocentrus hasethi* (Kraepelin, 1896): **DUTCH ANTILLES:** Curaçao: Rei Rinken Park, 12.183'N 69.965'W, 21.vii.2008, L. Esposito & D. Schiff, 1 ♀ (AMNH [LP 9065]); Christoffel Park, 12°21'15"N 69°06'13"W, 12.ix.2011, R. Jocqué & E. Tybaert, 2 ♀ (AMNH [LP 11208]).

*Diplocentrus rectimanus* Karsch, 1880 **MEXICO:** Oaxaca: Município de Etla: San Gabriel, 7 km N, 2152 m, 22.vii.2002, O.F. Francke, E. González & J. Ponce, 1 ♂, 3 ♀ (AMNH [LP 2032]).

*Heteronebo monticola* (Armas, 1999): **DOMINICAN REPUBLIC:** Provincia de Pedernales: Parque Nacional Sierra de Bahoruco, Cabo Rojo, 18°05'23.5"N 71°30'39.4"W, 1271 m, 10.vii.2004, E.S. Volschenk & J. Huff, 1 ♀, 1 subad. ♀ (AMNH [LP 3327]).

*Heteronebo oviedo* (Armas, 1999): **DOMINICAN REPUBLIC:** Provincia de Pedernales: Parque Nacional Jaragua: Los Tres Charcos, on road to Fondo Paradi, 1.6 km S of DR 44, 17°48.745'N 71°26.541'W, 74 m, 7–8.vii.2010, J. Huff & S. Schoenbrun, 1 ♂ (AMNH [LP 10525]); road to Cabo Rojo, 0.6 km S of DR 44, 17°58.201'N 71°39.036'W, 14 m, 7.vii.2010, J. Huff & S. Schoenbrun, 1 ♂ (AMNH [LP 10526).

*Kolotl poncei* (Francke & Quijana-Ravell, 2009): **MEXICO:** Michoacan: Município de La Huacana: El Vado, 18°48.908'N 101°54.976'W, 198 m, 20.v.2007, O.F. Francke, J. Ponce, et al., 1 juv. ♂ (AMNH [LP 7030]).

*Nebo hierichonticus* (Simon, 1872): **ISRAEL:** Haifa District: Mount Carmel National Park, Oren junction, campsite, lower Nahal Oren, N-facing slope, 32°42'50.3"N 34°58'37.6"E, 52 m, 22.viii.2011, L. Prendini, T.L. Bird & E. Gefen, 3 ♂ (AMNH).

*Oiclus purvesii* (Becker, 1880): **FEDERATION OF SAINT KITTS AND NEVIS:** Nevis: Trail behind Golden Rock Inn, 17.143°N 62.567°W, 1.vii.2008, L.A. Esposito & D. Schiff, 1 ♀ (AMNH [LP 9037]).

*Tarsoporosus kugleri* (Schenkel, 1932): **VENEZUELA:** xi.2005, ex E. Ythier, 1 ♂ (AMNH [LP 5204]).

*Tarsoporosus macuira* Teruel & Roncallo, 2010: **VENEZUELA:** Valera region, x.2005, S.E. Bazo Abreu, 2 ♂ (AMNH [LP 5510, 5511]).

**Family Euscorpiidae Laurie, 1896**

*Euscorpius italicus* (Herbst, 1800): **ITALY:** Famano, W of Rimini, 11.viii.2009, S. Huber, 1 ♂ (AMNH [LP 10297]).

*Euscorpius tergestinus* (C.L. Koch, 1837): **CROATIA:** Zadar, viii.2005, ex A. Tietz, 1 ♂ (AMNH [LP 4411]).

*Megacormus gertschi* Díaz Najera, 1966: **MEXICO:** Hidalgo: Zacualtipan, 4 km N, 2200 m, 18.viii.1973, T.C. Kasper, 1 ♂ (AMNH); Município de Acaxochitlan: Acaxochitlan, 2 km E, 20°08.5'N 98°10.74'W, 2214 m, 23.ix.2006, O.F. Francke, A. Valdez & H. Montaño, 1 juv. ♂ (AMNH [LP 6474]).

*Plesiochactas dilutus* (Karsch, 1881): **MEXICO:** Veracruz: Atoyac, 28.ii.1900, A. Dugés, ♀ (BMNH [1900.2.28.1]) [holotype of *Plesiochactas dugesi* Pocock, 1900].

*Plesiochactas mitchelli* Soleglad, 1976: **GUATEMALA:** Sarg, juv. ♀ holotype (BMNH).

*Troglocormus ciego* Francke, 1981: **MEXICO:** San Luis Potosí: Cueva de Elias, 13 km N of Agua Buena, 3.viii.1975, D. McKenzie, holotype ♂ (AMNH).

*Troglocormus willis* Francke, 1981: **MEXICO**: Tamaulipas: Conrado Castillo, P-2 Cave, 3.iv.2004, B. Shade, 1 ♂ (AMNH [LP 2743]); Cueva del Brinco, entrance passage, 3.v.1978, P. Sprouse, holotype ♂ (AMNH); Yerbabuena, Cueva de Esperanza, 20.xi.1979, P. Sprouse, 1 ♀ (AMNH).

**Family Hemiscorpiidae Pocock, 1893**

*Hemiscorpius lepturus* Peters, 1861: **IRAN:** Bushehr Province: 29°52'49''N 51°22'31''E, xi.2007, Masihipour, Bahrani & Hayader, 1 ♂ (AMNH [LP 11080]). Ilam Province: 32°46'32''N 47°39'78''E, x.2007, Masihipour, Navidpour & Bahrani, 1 ♀ (AMNH [LP 11081]).

**Family Heteroscorpionidae Kraepelin, 1905**

*Heteroscorpion kraepelini* Lourenço & Goodman, 2006: **MADAGASCAR:** Antsiranana Province: Montagne de Français, 7.2 km 142° SE of Antsiranana (Diego Suarez), 12°19'22"S 49°20'17"E, 180 m, 22–28.ii.2001, B.L. Fisher, C.E. Griswold et al., 1 ♂, 1 subad. ♂ (AMNH [LP 2706]).

*Heteroscorpion magnus* Lourenço & Goodman, 2002: **MADAGASCAR:** Antsiranana Province: Forêt de Binara, near Analamazava River, 7.5 km SW of Daraina, 13°15.3'S 40°37.0'E, 325–600 m, S.M. Goodman, paratype ♂ (FMNH); Forêt d’Antsahabe, 11.4 km 275° W of Daraina, 13°12'42''S 49°33'24''E, 550 m, 17.xi.2004, B.L. Fisher, 1 ♂, 1 subad. ♂ (AMNH [LP 8287]).

*Heteroscorpion* sp.: **MADAGASCAR:** Fianarantsoa Province: Forêt Classée Vatovavy, 7.6 km 122° ESE of Kianjavato, 21°24'00''S 47°56'24''E, B.L. Fisher, et al., 175 m, 6–8.vi.2005, B.L. Fisher et al., 1 subad. ♂, 1 juv. ♂ (AMNH [LP 8291]); Forêt de Vevembe, 66.6 km 293° WNW of Farafangana, 22°47'28''S 47°10'55''E, 600 m, 23.iv.2006, B.L. Fisher, et al., 1 juv. ♂ (AMNH [LP 8292]).

**Family Hormuridae Laurie, 1896**

*Cheloctonus jonesii* Pocock, 1892: **SOUTH AFRICA:** Limpopo Province: Soutpansberg District: Kruger National Park, Vlakteplaas Section, Xirhombe, ca. 20 km along dust road past roan enclosures from Punda Maria/Shingwedzi tar road, 22°43'38"S 31°22'36.9"E, 458 m, 4.i.2007, Spider Club of Southern Africa, 1 ♂, 3 ♀, 1 subad. ♂ (AMNH).

*Chiromachetes fergusoni* Pocock, 1899: **INDIA:** Kerala: Trivandrum, Travancore, Ponmudi, H. Ferguson, holotype ♀ (BMNH 1897.7.11.1).

*Chiromachus ochropus* (C.L. Koch, 1837): **SEYCHELLES:** Praslin: Vallée de Mai, v.2006, ex W.R. Lourenço, 1 subad. ♀ (AMNH [LP 6894]).

*Hadogenes troglodytes* (Peters, 1861): **MOZAMBIQUE:** Tete Region: Tete District: Tete, upper slope of hill with communications tower overlooking town, 16°12'22"S 33°34'06"E, 373 m, 10.xii.2007, L. Prendini & W.R. Schmidt, 1 subad. ♂, 1 subad. ♀ (AMNH).

*Hormiops davidovi* Fage, 1933: **VIETNAM:** Ba Ria-Vung Tau Province: Con Dao District: Con Dao National Park, Con Dao Archipelago, Con Son Island, Plot 58, Hunynh Thuc Khang Road, 08°41.232'N 106°35.711'E, 14.ii.2012, L. Prendini, H.T. Luu & H.S. Le, 2 ♂, 4 ♀ (AMNH).

*Hormurus extensus* (Locket, 1997): **AUSTRALIA:** Northern Territory: Kakadu National Park, Jim Jim Falls walking track, 13°16'24.3"S 132°49'56.3"E to 13°16'22.2''S 132°49'55.3''E, 64–129 m, 21–22.viii.2009, L. Monod & G. Brown, 1 juv. ♀ (AMNH [LP 10319]).

*Iomachus laeviceps* (Pocock, 1890): **INDIA:** Madras [Chennai], E. Thurston, 4 ♂, 2 ♀ (BMNH).

*Iomachus politus* Pocock, 1896: **TANZANIA:** Zanzibar: Jozani Forest Reserve, 6.268°S, 39.411°E, 19 m, 3.xi.2007, P.G. Hawkes, 1 ♂, 2 ♀ (AMNH).

*Liocheles australasiae* (Fabricius, 1775): **VIETNAM:** Binh Thuan Province: Ham Thuan Nam District: Ta Cu/Ta Kou Mountain Nature Reserve, Nui Ta Cu/Ta Kou, below summit, above reclining Buddha, 10°48'58.0"N 107°53'46.1"E, ca. 500 m, 17.ii.2012, L. Prendini, 3 ♀ (AMNH).

*Opisthacanthus elatus* (Gervais, 1844): **PANAMA:** Soberalie National Park, 15.x.2000, M. Makovec, 1 ♀ (AMNH [LP 1837]).

*Opisthacanthus lecomtei* (Lucas, 1858): **CAMEROON:** Mengalé, 03°06'N 10°52'E, 616 m, 12.x.2002, R.C. West, 1 ♀ (AMNH [LP 2194A]).

*Opisthacanthus madagascariensis* Kraepelin, 1894: **MADAGASCAR:** Marolinta Spiny Forest, 25°06.470'S 44°40.416'E, 50 m, 7.ii.2006, C. Raxworthy, 1 juv. (AMNH [LP 6263A]).

*Opisthacanthus validus* Thorell, 1876: **SOUTH AFRICA:** KwaZulu-Natal Province: Port Edward, Umtamvuna River Lodge, 31°03.968'S 30°11.265'E, 64 m, 12.i.2011, C. Haddad, 1 ♂, 1 subad. ♂ (AMNH).

*Palaeocheloctonus pauliani* Lourenço, 1996: **MADAGASCAR:** Toliara Province: Parc National de Tsimanampetsotsa, Mitoho Cave, 6.4 km 77° ENE of Efoetse, 17.4 km 170° S of Beheloka, 24°02'50''S 43°45'11''E, 40 m, 18–22.iii.2002, B.L. Fisher, C.E. Griswold, et al., 3 ♂, 2 juv. (AMNH [LP 6257A]).

**Family Iuridae Thorell, 1876**

*Anuroctonus phaiodactylus* (Wood 1863): **U.S.A.:** Utah: Toole County: viii.1983, 1 ♂ (AMNH).

*Calchas anlasi* Yağmur et al., 2013: **TURKEY:** Hakkari Province: Çukurca, 37°14'56.5''N 43°36'11.8''E, 1163 m, 24.vi.2007, A.V. Gromov & H. Koc, 1 ♀ (AMNH [LP 7331]).

*Calchas birulai* Fet et al., 2009: **TURKEY:** Gaziantep Province: Antep-Sehitkamil, Incesu Köyü, 7.v.2005, E.A. Yağmur, 1 ♂ (AMNH [LP 4333]); Kurtulus, 37°01'13.7''N 37°27'17.4''E, 842 m, 14.v.2007, A.V. Gromov, H. Koç, E.A. Yağmur, M. Yalçin, et al., 1 ♂ (AMNH [LP 7334]); Yamacova, 1 km E, 37°10'30.6"N 37°06'07.9"E, 1138 m, 1.vii.2007, A.V. Gromov & H. Koç, 1 ♀ (AMNH). Sanliurfa Province: Karacadag, S slope, 37°32'26.7''N 39°49'55.1''E, 1303 m, 11–12.vi.2007, A.V. Gromov & H. Koç, 1 ♀ (AMNH [LP 7330]).

*Caraboctonus keyserlingii* Pocock, 1893: **CHILE:** Región IV (Coquimbo): Monumento Natural Pichasca, 30°23'48.8"S 70°52'54.0"W, 767 m, 26.i.2005, C.I. Mattoni & A.A. Ojanguren, 2 ♂, 1 subad. ♀, 1 juv. ♂ (AMNH).

*Hadruroides charcasus* (Karsch, 1879): **PERU:** Provincia de Lambayeque: Lambayeque, EPM Anchovira, between Motupe and Jayanca, near intersection to Salas, 06°16.045'S 79°44.178'W, 101 m, 17.i.2008, R. Gutiérrez, D. Apaza & J.A. Ochoa, 2 ♂, 1 ♀, 1 subad. ♂, 1 juv. ♂ (AMNH).

*Hadrurus arizonensis* Ewing, 1928: **MEXICO:** Sonora: Município de Huatabampo: Huatabampito coastal sand dunes, 26°41'52.53''N 109°36.20.78''W, sea level, 24.viii.2009, E. González & J.L. Castelo, 2 ♂, 1 juv. ♀ (IBUNAM).

*Hoffmannihadrurus aztecus* (Pocock, 1902): **MEXICO:** Oaxaca: Município de Cuicatlán: Tomellin, 17°45.180'N 96°57.237'W, 605 m, 23.vii.2002, L. Prendini, O.F. Francke, E. González & J. Ponce, 1 ♂ (AMNH [LP 2041]), 1 subad. ♂ (AMNH [LP 2042]).

*Hoffmannihadrurus gertschi* (Soleglad, 1976): **MEXICO:** Guerrero: Chilpancingo, 1.x.1975, M.E. Soleglad, paratype ♀ (AMNH).

*Iurus dufoureius* (Brulle, 1832): **Greece:** Peloponnese: Louisos gorge, “Krypho Scholio” near Philosophou monastery, 37°33'15"N 22°02'45"E, iv.2008, S. & P. Weygoldt, 1 juv. (AMNH [LP 9844]).

*Neocalchas gruberi* (Fet et al., 2009): **TURKEY:** Mersin Province: Anamur, 15 km W, 36°02'30.4''N 32°44'34.4''E, ca. 100 m, 3.iv.2005, S. Huber, 1 ♀ (AMNH [LP 6222]).

*Protoiurus kraepelini* (von Ubisch, 1922): **TURKEY:** Antalya Province: Antalya District, Yağca village, near Döşemealtí, Karain Mağarasí Cave, Yanartaş, Dağí Mts., 37°04'663''N 30°34'239''E, 385 m, 14.vii.2006, P. Stoev, S. Lazarov, A. Topcu & T. Turkes, 1 ♀ (AMNH [LP 6895]); Kas, 5–7.v.1981, B. Malkin, 1 ♀ (AMNH). Mersin Province: Anemouryon (Anemourion), near Anamur, ca. 250 km E of Antalya, 36°01'27.4"N 32°48'09.5"E, ca. 35 m, iv.2005, S. Huber, 1 juv. ♀ (AMNH [LP 4218]), 1 ♀ (AMNH [LP 9845]); Cennet sinkhole, near Silifke, ca. 350 km E of Antalya, 36°27'08.2"N 34°06'22.3"E, ca. 100 m, 6.iv.2005, S. Huber, 1 subad. ♀ (AMNH [LP 4217]).

**Family Pseudochactidae Gromov, 1998**

*Pseudochactas ovchinnikovi* Gromov, 1998: **UZBEKISTAN:** Surkhandarya Area: Uzun District: Dikhana Canyon, foothills of E slopes of Babatag Mountain Range, ca. 5 km WSW of Akmechet village, 38°01.638'N 68°15.198'E, 20–24.v.2003, 722 m, L. Prendini & A.V. Gromov, 8 subad. ♂, 4 subad. ♀ (AMNH).

*Troglokhammouanus steineri* Lourenço, 2007: **LAOS:** Khammouane Province: Boualapha District: Hin Namno National Biodiversity Conservation Area, Tham Xe Bang Fai (Xe Bang Fai River Cave), left bank of Xe Bang Fai River (coming from downstream entrance), 159 m, 19.ii.2012, L. Prendini & P. Kanyavong, 2 ♀, 1 subad. ♂, 4 juv. ♂, 1 juv. ♀ (AMNH).

*Vietbocap lao* Lourenço, 2012**: LAOS:** Khammouane Province: Gnommalath District: Tham Nam Lot (Lod) near Ban Naden village, 260 m, 13–14.vi.2012, L. Prendini, S.F. Loria & P. Kanyavong, 2 juv. ♀ (AMNH [LP 11349, 11350]).

**Family Scorpionidae Latreille, 1802**

*Heterometrus cyaneus* (C.L. Koch, 1836): **INDONESIA:** West Java: Cianjur, Ciseureuh, 06°41'39.0"S 107°01'54.4"E, 1115 m, 4.vii.2013, S.F. Loria, 1 subad. ♂, 1 subad. ♀, 1 juv. ♂ (AMNH).

*Opistophthalmus jenseni* (Lamoral, 1972): **NAMIBIA:** Kunene Region: Khorixas District: Farm Bethanis 415, Damaraland, 17.xii.1988, A. Harington, 7 ♂, 1 ♀ AMNH ([AH 4039–4046]). **NAMIBIA:** Kunene Region: Khorixas District: Twyfelfontein, 20°35.729'S 14°22.346'E, 582 m, 21.i.2004, L. Prendini, E. Scott, T.L. & C. Bird, Q. & N. Martins, 4 ♂ (AMNH).

*Opistophthalmus* sp.: **NAMIBIA:** Karas Region: Lüderitz District: Namib-Naukluft Park, Koichab River bed, 2.1 km W of intersection between Haalenberg–pumpstation road and Garub–pumpstation road, 573 m, 25.i.2009, L. Prendini, T.L. Bird & J. Huff, 12 ♂ (AMNH).

*Pandinus gregoryi* (Pocock, 1896): **KENYA**: Rift Valley Province: Lake Bogoria, Loboi Location, 1 km S of Lake Bogoria Hotel, 00°20.863'N 36°03.564'E, 3480 ft, 19–25.xi.2005, S. Mwangi, 1 ♀, 1 subad. ♂, 1 subad. ♀, 3 juv. ♂ (AMNH).

*Scorpio maurus palmatus* (Ehrenberg, 1828): **ISRAEL:** Southern District: Mamshit, road to Negev Camel Ranch, 31°01'59.9"N 35°04'25"E, 477 m, 26.viii.2011, L. Prendini & T.L. Bird, 4 ♂ (AMNH).

**Family Scorpiopidae Kraepelin, 1905**

*Alloscorpiops* sp.: **THAILAND:** Kanchanaburi Province: Thong Pha Phum, reforestation natural centre, 14°40'05.3"N 98°35'40.7"E, 174 m, 22.xi.2011, O. Košulič & W. Chotwong, 1 ♀ (AMNH [LP 11279]).

*Euscorpiops kaftani* (Kovařík, 1993): **VIETNAM:** Ninh Binh Province: Nhoquan District: Cuc Phuong National Park, Thousand Year Old Tree Loop Trail, Khu Trung Tam Park Center (Bong camp), ca. 30 km NW of park headquarters, 20°20'54.3"N 105°35'52.2"E, 365 m, 11.vi.2012, L. Prendini & S.F. Loria, 1 juv. ♀ (AMNH [LP 11371]).

*Euscorpiops problematicus* Kovařík, 2000: **THAILAND:** Chiang Mai Province: above Puping Palace, 18°48.592'N 98°53.624'E, 1409 m, 16.xii.2003, S. Huber, 1 ♀ (AMNH [LP 2847]).

*Parascorpiops montana* Banks, 1928: **MALAYSIA:** Sarawak: Mt. Poi, 5400 ft, E. Mjöberg, lectotype ♂, paralectotype ♀ (MCZ); Kubah National Park, Matang, 4.2 km from front gate, 01°35'31.6"N 110°11'34.7"E, 657 m, 5–6.viii.2013, J. Huff & A. Ang, 3 ♂, 1 ♀ (AMNH).

*Scorpiops feti* Kovařík, 2000: **INDIA:** Teesta Valley, Bhutan border, under stone, 400 ft, 1 ♂, 1 juv. ♀ (MCZ).

**Family Superstitioniidae Stahnke, 1940**

*Superstitionia donensis* Stahnke, 1940: **U.S.A**.**:** Arizona: Cochise County: Portal, ix.1966, W J. Gertsch, 1 ♂ (AMNH); Portal, 1.5 mi. NE, 17.iii.1961, Cazier & Mortenson, 1 ♀ (AMNH); Southwestern Research Station, 5 mi. W of Portal, 15.vii.1955, W.J. Gertsch, 1 ♀ (AMNH); Whitetail draw, 13.8 mi. NE of Portal, 21.i.1961, M. Cazier, 1 ♀ (AMNH).

**Family Troglotayosicidae Lourenço, 1998**

*Belisarius xambeui* Simon, 1879: **FRANCE:** East Pyrenees, ca. 3 km above Prats-de-Mollo, 42°23.707'N 02°28.546'E, 2725 ft, 10.vi.2003, S. Huber, 1 ♀ (AMNH [LP 2904]). **SPAIN:** Girona: Fajeda d’en Jordà, 5.xi.1993, G. Giribet & C. Ribera, 1 ♀ (AMNH [LP 1629]).

*Troglotayosicus humiculum* Botero-Trujillo & Francke, 2009: **COLOMBIA:** Departamento de Nariño: Município de Ricaurte: Vereda Alto Cartagena, Finca Nueva Estrella, 01°13.262'N 77°58.143'W, 12.ix.2008, R. Botero, J.P. Botero & J.A. Ochoa, 2 ♀, 1 ♂ (AMNH).

**Family Typhlochactidae Mitchell, 1971**

*Alacran tartarus* Francke, 1982 **MEXICO:** Oaxaca: Município de San Miguel: Cueva de Escorpión, 18.107°N 96.798°W, 1561 m, 18.ix.2004, A. Gluesenkamp, P. Sprouse & C. Savvas, 1 ♀ (AMNH [LP 3499]).

*Alacran* sp.: **MEXICO:** Puebla: Município de Tlacotepec de Díaz: Cueva las Tres Quimeras, 1440 m, 1.iv.2009, B. Shade, 1 ♂ (AMNH [LP 9983]).

*Sotanochactas elliotti* (Mitchell, 1971): **MEXICO:** San Luis Potosí: Município de Ciudad Valles: El Sótano de Yerbaniz, 22.5 km N of Ciudad Valles, 31.vii.1969, W.R. Elliott, subad. ♂ holotype (AMNH); Sótano de Yerbaniz, 4.vii.1970, W. Mitchell, paratype ♂ (WDS).

*Stygochactas granulosus* (Sissom & Cokendolpher, 1998): **MEXICO:** Veracruz: Município de Tlaquilpa: Sótano de Poncho, 2.iii.1995, P. Sprouse, juv. ♂ holotype (AMNH).

*Typhlochactas mitchelli* Sissom, 1988: **MEXICO:** Oaxaca: Município de San Jose Tenango: Cerro Ocote, 5 mi. S of San Jose de Tenango, iv.1987, A. Grubbs, A. Cressler & P. Smith, holotype ♂ (AMNH).

*Typhlochactas rhodesi* Mitchell, 1968: **MEXICO:** Tamaulipas: Município de Gómez Farias: La Cueva de la Mina, Sierra de Guatemala, 1600 m, 24.iii.1967, R.W. & R. Mitchell, K. Pittard, D. Falls & V. Colvin, holotype ♀ (AMNH).

**Family Urodacidae Pocock, 1893**

*Aops oncodactylus* Volschenk & Prendini, 2008: **AUSTRALIA:** Western Australia: Barrow Island, Ledge Cave (Cave B-1), second chamber, 20°47'53"S 115°19'53"E, 21.viii.1999, W.F. Humphreys & S.M. Eberhard, juv. ♀ holotype (WAM 99/2109).

*Urodacus* *manicatus* (Thorell, 1876): **AUSTRALIA:** New South Wales: University of New England Campus, 21.i.1980, 1 ♂, 1 ♀ (AMNH).

*Urodacus* sp.: Northern Territory: Litchfield National Park, road near Walker Creek turnoff, outside ranger station, 13°04'40.3"S 130°41.32.1"E, 31.viii.2009, L. Monod, 2 juv. ♂ (AMNH).

**Family Vaejovidae Thorell, 1876**

*Chihuahuanus globosus* (Borelli, 1915): **MEXICO:** Município de Viesca: Dunas de Bilbao, 25°25.586'N 102°53.654'W, 1098 m, 20.vii.2006, O.F. Francke, K.J. McWest, M. Cordova, A. Jaimes & A. Ballesteros, 1 ♂ (AMNH [LP 6459]). **U.S.A.:** Texas: Brewster County: Big Bend National Park, Boquillas Canyon, 29.200°N 102.917°W, 2.viii.2008, W.D. Sissom, T. Anton & G. Casper, 1 ♂, 1 juv. (AMNH [LP 8926]).

*Franckeus nitidulus* (C.L. Koch, 1843): **MEXICO:** Queretaro: Vizarrón, 5 km N, 20°53.459'N 99°41.605'W, 1822 m, 30.iv.2005, O.F. Francke, et al., 2 ♂, 1 ♀ (AMNH [LP 4115]).

*Gertschius crassicorpus* Graham & Soleglad, 2007: **MEXICO:** Sonora: Município de Navojoa: Cerro Masiaca Microwave Station, ca. 35 km SW of Navojoa, 26°46'30.32"N 109°17'59.17"W, 193 m, 21.viii.2009, E. González & J.L. Castelo, 1 ♂, 1 ♀, 1 subad. ♂, 1 subad. ♀, 1 juv. ♂ (AMNH).

*Kochius bruneus* (Williams, 1970): **MEXICO:** Baja California Sur: Município de Comondú: San Miguel de Comondú, 2 km SW, 26°00'18.5"N 111°52'1.3W, 169 m, 12.vii.2004, W.E. Savary, A. Valdez, E. González & O.F. Francke, 4 ♂, 1 ♀ (CAS).

*Kovarikia angelenus* (Gertsch & Soleglad, 1972): **U.S.A.:** California: Ventura County: Little Sycamore Canyon, rock walls along Yerba Buena Road, 34°03'37.6"N 118°57'52.2"W, 48 m, 22.viii.2004, R. Mercurio, W.E. Savary, M. McCoy & K. Bamba, 1 ♂ [AMNH [LP 3201].

*Kuarapu purhepecha* Francke & Ponce-Saavedra, 2010: **MEXICO:** Michoacan: Município de Huarana: El Bado [El Vado], 18.814°N 101.916°W, 248 m, 30.vii.2008, O.F. Francke, H. Montaño, J. Ponce & A. Quijano, 2 juv. (AMNH [LP 9517]).

*Maaykuyak vittatus* (Williams, 1970): **MEXICO:** Baja California Sur: Município de Los Cabos: Santiago, 23°26'24.5"N 109°43'34.6"W, 225 m, 9.vii.2004, O.F. Francke, E. González & A. Valdez, 3 ♀ (AMNH [LP 3181]).

*Mesomexovis oaxaca* (Santibañez-Lopez & Sissom, 2010): **MEXICO:** Oaxaca: Nueve Puntas, 13.vii.1963, G. Sluder, 3 ♀ (AMNH).

*Paravaejovis pumilis* (Williams, 1970): **MEXICO:** Baja California Sur: Município de Comondú: Ciudad Constitución, 32 km SW, 24°55'17"N 111°58'5.5"W, 26 m, 11.vii.2004, O.F. Francke, W.E. Savary, E. González & A. Valdez, 2 ♂ (AMNH [LP 3137]); Ciudad Constitución, 43 km W, 24°51'46"N 112°03'50.8"W, 7 m, 11.vii.2004, O.F. Francke, W.E. Savary, E. González & A. Valdez, 1 ♀ (AMNH).

*Paruroctonus surensis* Williams & Haradon, 1980: **MEXICO**: Baja California Sur: Município de Mulegé: Guerrero Negro, 12 km S, 27°56'5.8"N 113°54'23.1"W, 25 m,15.vii.2004, O.F. Francke, W.E. Savary, E. González & A. Valdez, 3 ♂ (AMNH [LP 3139]).

*Pseudouroctonus apacheanus* (Gertsch & Soleglad, 1972): **U.S.A.:** Arizona: Cochise County: Southwestern Research Station, Chiricahua Mountains, 31°53'00"N 109°12'22"W, 1641 m, 9.v.2005, T. Dikow, 1 ♀ (AMNH [LP 4094]). Pima County: Santa Rita Mountains, Madera Canyon, Bog Springs campground, 9.iv.1997, D. Ubick & W.E. Savary, 1 ♀ (AMNH [LP 4372]).

*Pseudouroctonus reddelli* (Gertsch & Soleglad, 1972): **U.S.A.:** Texas: Bexar County: backside of Government Canyon State Park along rock cut on Hwy 211, just past San Antonio city limit, 29°32'15.5"N 98º48'37.8"W, 318 m, 20.vii.2007, J. Huff, 1 ♂ (AMNH [LP 7021]).

*Serradigitus wupatkiensis* (Stahnke, 1940): **U.S.A.:** California: Inyo County: “The Narrows” on Route 168, 37°15.270'N 118°09.376'W, 5.ix.2005 , 2100 m, L. Prendini & R. Mercurio, 2 ♂ (AMNH [LP 5031]).

*Smeringurus grandi*s (Williams, 1970): **MEXICO:** Baja California: Município de Ensenada: 4 km S of turnoff to Punta Bufeo from road to San Felipe, 29°51.433'N 114°26.500'W, 28 m, 14.vii.2005, E. González, W.E. Savary, L. Prendini & R. Mercurio, 1 juv. ♂, 2 juv. ♀ (AMNH [LP 4462]).

*Stahnkeus subtilimanus* (Soleglad, 1972): **U.S.A.:** California: Riverside County: Little San Bernardino Mountains, Berdoo Canyon, 2.98 mi. NE of Berdoo Canyon Road/Dillon Road junction on Berdoo Canyon Road, 33°49'39.27"N 116°09'4.26"W, 3.viii.2008, Z.J. Valois & W.E. Savary, 1 ♀ (AMNH).

*Syntropis macrura* Kraepelin, 1900: **MEXICO:** Baja California Sur: Município de Comondú: San Miguel de Comondú, 2 km SW, 26°00'18.5"N 111°52'1.3"W, 169 m, 12.vii.2004, W.E. Savary, A. Valdez, E. González & O.F. Francke, 2 juv. ♀ (AMNH).

*Thorellius intrepidus* (Thorell, 1876): **MEXICO:** Colima: Município de Comala: Comala, 19°19.00'N 103°45.00'W, 9.iv.2004, P. Berea, 1 subad. ♀ (AMNH [LP 6377]); Los Ortices, 19°06'46.8''N 103°44'22.6''W, 9.iv.2004, P. Berea, 1 ♂ (AMNH [LP 6379]). Michoacan: Município de Aquila: Faro de Bucerias, 10 m, 13–14.i.2002, E. González, 1 ♀ (AMNH [LP 2022]).

*Uroctonites giulianii* Williams & Savary, 1991: **U.S.A.:** California: Inyo County: “The Narrows” on Route 168, 37°15.270'N 118°09.376'W, 2100 m, 5.ix.2005, L. Prendini & R. Mercurio, 1 ♂ (AMNH).

*Uroctonus mordax* Thorell, 1876: **U.S.A.:** California: Napa County: Butts Canyon Road just N of Calistoga, 3 mi. SE of Lake County line, 38°41'21.1"N 122°26'47.4"W, 246 m, 20.viii.2006, J. Huff & W.E. Savary, 2 ♂ (AMNH [LP 6278]).

*Uroctonus mordax pluridens* Hjelle, 1972: **U.S.A.:** California: Santa Cruz County: Big Basin Ranger Station, 4.5 mi. S, 14.iii.2004, W.E. Savary & E.S. Volschenk, 1 ♂, 1 ♀ (AMNH [LP 2686]).

*Vaejovis carolinianus* (Beauvois, 1805): **U.S.A.:** Louisiana: West Feliciana Parish: near Retreat, Louisiana, and S of Pinckneyville, Mississippi, on LA 969 (Pinckneyville Road), 0.3 mi. N of junction LA 66 (Tunica Trace), 30°59.077'N 91°29.449'W, 3.v.2008, K.J. & M.N. McWest, 1 ♀ (AMNH [LP 8499]). Mississippi: Wilkinson County: near Pond, 11.4 mi. W of Woodville, on Pinckneyville Road, 31°05.511'N 91°28.335'W, 2.v.2008, K.J. & M.N. McWest, 1 ♀ (AMNH [LP 8500]).

*Vaejovis mexicanus* C.L. Koch, 1836: **MEXICO:** Distrito Federal: Delegacion Coyoacan, 19°20'58.54"N 99°09'42.35"W, 18.xii.2001, E. González, 1 ♀ (AMNH [LP 1825]).

*Vaejovis vorhiesi* Stahnke, 1940: **U.S.A.:** Arizona: Santa Cruz County: Santa Rita Mountains, Madera Canyon, above Mt. Wrightson Trailhead Parking, 31.712°N 110.874°W, 5470 ft/1667 m, 25.vii.2008, H.M. Burrell & K.J. McWest, 2 ♀ (AMNH [LP 8860]).

*Vejovoidus longiunguis* (Williams, 1969): **MEXICO:** Baja California: Santa Rosalillita, 28°39.961'N 114°14.578'W, 6 m, 27.v.2004, R. Mercurio, M. Nishiguchi, et al., 1 ♀ (AMNH [LP 2870]). Baja California Sur: Município de Guerrero Negro: San Ángel dunes, 27°14'05"N 113°13'46.5"W, 25 m,14.vii.2004, O.F. Francke, W.E. Savary, E. González & A. Valdez, 1 ♀ (AMNH [LP 3148]). Município de Mulegé: Guerrero Negro, 12 km S, 27°56'5.8"N 113°54'23.1"W, 25 m, 15.vii.2004, O.F. Francke, W.E. Savary, E. González & A. Valdez, 1 ♀ (AMNH [LP 3147]).

*Vizcaino viscainensis* (Williams, 1970): **MEXICO:** Baja California Sur: Município de Guerrero Negro: Desierto del Vizcaino, 27°29'54.7''N 114°17'35.6''W, 223 m, 16.vii.2004, O.F. Francke, W.E. Savary, E. González & A. Valdez, 2 ♀ (AMNH [LP 3171]).

*Wernerius mumai* (Sissom, 1993): **U.S.A.:** Arizona: Mohave County: Black Mountains, Sitgreaves Pass, E side on Oatman Hwy, 35°02.287'N, 114°21.070'W, 982 m, 12.viii.2007, W.D. Sissom, G. Casper & T. Anton, 1 ♂ (AMNH [LP 7002]); Sitgreaves Pass, W side, 1.8 mi. W Gold Road on Oatman Hwy, 35°02.678'N 114°23.200'W, 832 m, 12.viii.2007, W.D. Sissom, T. Anton & G. Casper, 1 ♂ (AMNH [LP 9000]).
