# Supplementary material for: Homology of the Lateral Eyes of Scorpiones: A Six-Ocellus Model
Source: PLoS One. 2014 Dec 3;9(12):e112913. doi: 10.1371/journal.pone.0112913 (PMC4254604; doi:10.1371/journal.pone.0112913)
Supplement: Appendix S2 — Lateral ocelli of Recent scorpion families: Counts, presence or absence, relative size (larger than, equal to or smaller than), and presence or absence of eyespot in material examined. Sample size (n) represents number of individuals per species. Counts provide the range observed in the sample for sinistral and dextral sides of carapace. Presence/absence data provide the count of individuals observed with particular ocelli or eyespot on sinistral and dextral sides of carapace. Abbreviations: AALMa (accessory anterolateral major ocellus); ADMi (anterodorsal minor ocellus); ALMa (anterolateral major ocellus); APLMi1, APLMi2, APLMi3 (accessory posterolateral minor ocelli); e (eyespot); MLMa (mediolateral major ocellus); PDMi (posterodorsal minor ocellus); PLMa (posterolateral major ocellus); PLMi (posterolateral minor ocellus). (DOC) [file pone.0112913.s002.doc]

**Appendix S2.** **Lateral ocelli of Recent scorpion families: Counts, presence or absence, relative size (larger than, equal to or smaller than), and presence or absence of eyespot in material examined.** Sample size (*n*) represents number of individuals per species. Counts provide the range observed in the sample for sinistral and dextral sides of carapace. Presence/absence data provide the count of individuals observed with particular ocelli or eyespot on sinistral and dextral sides of carapace. Abbreviations: AALMa (accessory anterolateral major ocellus); ADMi (anterodorsal minor ocellus); ALMa (anterolateral major ocellus); APLMi_1_, APLMi_2_, APLMi_3_ (accessory posterolateral minor ocelli); e (eyespot); MLMa (mediolateral major ocellus); PDMi (posterodorsal minor ocellus); PLMa (posterolateral major ocellus); PLMi (posterolateral minor ocellus)).

**Family Akravidae Levy, 2007**

*Akrav israchanani* Levy, 2007 (*n* = 7): Ocelli count: 0/0. Eyespot absent (7/7).

**Family Bothriuridae Simon, 1880**

*Bothriurus bonariensis* (C.L. Koch, 1842) (*n* = 5): Ocelli count: 3/3. Ocelli present, relative size: MLMa (5/5) > PLMa (5/5) ≥ PDMi (5/5). Eyespot absent (5/5).

*Bothriurus vittatus* (Guérin Méneville, 1838) (*n* = 3): Ocelli count: 3/3. Ocelli present, relative size: MLMa (3/3) > PLMa (3/3) ≥ PDMi (3/3). Eyespot absent (3/3).

*Brachistosternus multidentatus* Maury, 1984 (*n* = 2): Ocelli count: 3/3. Ocelli present, relative size: MLMa (2/2) > PLMa (2/2) ≤ PDMi (2/2). Eyespot absent (2/2).

*Brandbergia haringtoni* Prendini, 2003 (*n* = 2): Ocelli count: 3/3. Ocelli present, relative size: MLMa (2/2) ≥ PLMa (2/2) = PDMi (2/2). Eyespot absent (2/2).

*Centromachetes* sp. (*n* = 2): Ocelli count: 3/3–4. Ocelli present, relative size: MLMa (2/2) ≥ PLMa (2/2) ≥ PDMi (2/2) > ADMi (-/1). Eyespot absent (2/2).

*Cercophonius sulcatus* Kraepelin, 1908 (*n* = 1): Ocelli count: 3/3. Ocelli present, relative size: MLMa (1/1) > PLMa (1/1) > PDMi (1/1). Eyespot absent (1/1).

*Lisposoma elegans* Lawrence, 1928 (*n* = 5): Ocelli count: 3/3–4. Ocelli present, relative size: ALMa (-/1) < MLMa (5/4) ≥ PLMa (5/4) ≤ PDMi (5/4). Eyespot absent (5/5).

*Lisposoma josehermana* Lamoral, 1979 (*n* = 1): Ocelli count: 3/3. Ocelli present, relative size: MLMa (1/1) = PLMa (1/1) = PDMi (1/1). Eyespot absent (1/1).

*Orobothriurus paessleri* (Kraepelin, 1911) (*n* = 1): Ocelli count: 3/3. Ocelli present, relative size: MLMa (1/1) > PLMa (1/1) = PDMi (1/1). Eyespot absent (1/1).

*Pachakutej inca* (Maury, 1975) (*n* = 1): Ocelli count: 3/3. Ocelli present, relative size: MLMa (1/1) > PLMa (1/1) > PDMi (1/1). Eyespot absent (1/1).

*Phoniocercus sanmartini* Cekalovic, 1968 (*n* = 5): Ocelli count: 3–4/3. Ocelli present, relative size: MLMa (5/5) ≥ PLMa (5/5) > PDMi (5/5) > ADMi (1/-). Eyespot absent (5/5).

*Rumikiru atacama* Ojanguren-Affilastro et al., 2012 (*n* = 6): Ocelli count: 3/3. Ocelli present, relative size: MLMa (6/6) ≥ PLMa (6/6) ≥ PDMi (6/6). Eyespot absent (6/6).

*Thestylus glasioui* (Bertkau, 1880) (*n* = 1): Ocelli count: 3/3. Ocelli present, relative size: MLMa (1/1) ≥ PLMa (1/1) > PDMi (1/1). Eyespot absent (1/1).

*Timogenes mapuche* Maury, 1975 (*n* = 2): Ocelli count: 3/3. Ocelli present, relative size: MLMa (2/2) ≥ PLMa (2/2) ≥ PDMi (2/2). Eyespot absent (2/2).

*Urophonius granulatus* Pocock, 1898 (*n* = 6): Ocelli count: 3/3. Ocelli present, relative size: MLMa (6/6) ≥ PLMa (6/6) ≥ PDMi (6/6). Eyespot absent (6/6).

*Vachonia martinezi* Abalos, 1954 (*n* = 1): Ocelli count: 2/2. Ocelli present, relative size: PLMa (1/1) > PDMi (1/1). Eyespot absent (1/1).

**Family Buthidae C.L. Koch, 1837**

*Afroisometrus minshullae* (FitzPatrick, 1994) (*n* = 1): Ocelli count: 2/3. Ocelli present, relative size: MLMa (1/1) < PLMa (1/1) > PDMi (-/1). Eyespot present (?/1).

*Akentrobuthus atakora* Vignoli & Prendini, 2008 (*n* = 1): Ocelli count: 3/2. Ocelli present, relative size: MLMa (1/1) ≥ PLMa (1/1) > ADMi (1/-). Eyespot absent (1/1).

*Alayotityus nanus* Armas, 1973 (*n* = 1): Ocelli count: 3/4. Ocelli present, relative size: ALMa (-/1) < MLMa (1/1) < PLMa (1/1) > ADMi (1/1). Eyespot present (1/1).

*Ananteris inoae* González-Sponga, 2006 (*n* = 1): Ocelli count: 4/4. Ocelli present, relative size: ALMa (1/1) > < MLMa (1/1) > PLMa (1/1) > ADMi (1/1). Eyespot absent (1/1).

*Androctonus finitimus* (Pocock, 1897) (*n* = 5): Ocelli count: 4–5/5. Ocelli present, relative size: ALMa (5/5) ≥ ≤ MLMa (5/5) ≥ PLMa (5/5) > PDMi (4/5) ≥ ≤ ADMi (5/5). Eyespot present?

*Anomalobuthus rickmersi* Kraepelin, 1900 (*n* = 3): Ocelli count: 5/5. Ocelli present, relative size: ALMa (3/3) ≤ MLMa (3/3) ≥ PLMa (3/3) > PDMi (3/3) ≥ ≤ ADMi (3/3). Eyespot present?

*Apistobuthus pterygocercus* Finnegan, 1932 (*n* = 1): Ocelli count: 5/5. Ocelli present, relative size: ALMa (1/1) = MLMa (1/1) > PLMa (1/1) > PDMi (1/1) > < ADMi (1/1). Eyespot present?

*Australobuthus xerolimniorum* Locket, 1990 (*n* = 1): Ocelli count: 5/5. Ocelli present, relative size: ALMa (1/1) < MLMa (1/1) ≤ PLMa (1/1) > PDMi (1/1) > ADMi (1/1). Eyespot present?

*Babycurus gigas* Kraepelin, 1896 (*n* = 2): Ocelli count: 4–5/5. Ocelli present, relative size: ALMa (2/2) < MLMa (2/2) > PLMa (2/2) > ADMi (2/2) > PDMi (1/2). Eyespot present (2/2).

*Birulatus haasi* Vachon, 1974 (*n* = 1): Ocelli count: 4/3. Ocelli present, relative size: ALMa (1/1) < MLMa (1/1) = PLMa (1/1) > ADMi (1/-). Eyespot present?

*Buthacus macrocentrus* (Ehrenberg, 1828) (*n* = 3): Ocelli count: 5/5. Ocelli present, relative size: ALMa (3/3) ≤ MLMa (3/3) ≥ PLMa (3/3) > PDMi (3/3) ≥ ≤ ADMi (3/3). Eyespot present?

*Butheoloides milloti* Vachon, 1948 (*n* = 1): Ocelli count: 4/5. Ocelli present, relative size: ALMa (1/1) < MLMa (1/1) > PLMa (1/1) > PDMi (1/1) > ADMi (-/1). Eyespot absent (1/1).

*Butheoloides monodi* Vachon, 1950 (*n* = 2): Ocelli count: 5/5. Ocelli present, relative size: ALMa (2/2) > < MLMa (2/2) ≥ PLMa (2/2) > PDMi (2/2) ≤ ADMi (2/2). Eyespot absent (2/2).

*Butheolus gallagheri* Vachon, 1980 (*n* = 2): Ocelli count: 5/5. Ocelli present, relative size: ALMa (2/2) > < MLMa (2/2) > PLMa (2/2) > ADMi (2/2) > PDMi (2/2). Eyespot present (2/2).

*Buthiscus bicalcaratus* Birula, 1905 (*n* = 1): Ocelli count: 5/5. Ocelli present, relative size: ALMa (1/1) < MLMa (1/1) > PLMa (1/1) > PDMi (1/1) > ADMi (1/1). Eyespot present?

*Buthoscorpio rayalensis* Javed et al., 2010 (*n* = 1): Ocelli count: 5/5. Ocelli present, relative size: ALMa (1/1) < MLMa (1/1) > PLMa (1/1) > ADMi (1/1) > PDMi (1/1). Eyespot present (1/1).

*Buthoscorpio sarasinorum* (Karsch, 1891) (*n* = 1): Ocelli count: 5/5. Ocelli present, relative size: ALMa (1/1) < MLMa (1/1) > PLMa (1/1) > ADMi (1/1) > PDMi (1/1). Eyespot present (1/1).

*Buthus malhommei* (Vachon, 1949) (*n* = 3): Ocelli count: 5/5. Ocelli present, relative size: ALMa (3/3) ≥ MLMa (3/3) ≥ PLMa (3/3) > PDMi (3/3) ≥ ≤ ADMi (3/3). Eyespot present (3/3).

*Centruroides vittatus* (Say, 1821) (*n* = 2): Ocelli count: 5/5. Ocelli present, relative size: ALMa (2/2) < MLMa (2/2) > PLMa (2/2) > ADMi (2/2) > PDMi (2/2). Eyespot present (2/2).

*Charmus minor* Lourenço, 2002 (*n* = 1): Ocelli count: 4/4. Ocelli present, relative size: ALMa (1/1) < MLMa (1/1) > PLMa (1/1) > ADMi (1/1). Eyespot present?

*Cicileus exilis* (Pallary, 1928) (*n* = 5): Ocelli count: 5/5. Ocelli present, relative size: ALMa (5/5) ≤ MLMa (5/5) ≥ ≤ PLMa (5/5) > ADMi (5/5) > PDMi (5/5). Eyespot present (5/5).

*Compsobuthus levyi* Kovařík, 2012 (*n* = 4): Ocelli count: 5/5. Ocelli present, relative size: ALMa (4/4) ≤ MLMa (4/4) > PLMa (4/4) > ADMi (4/4) > < PDMi (4/4). Eyespot present?

*Grosphus ankarana* Lourenço & Goodman, 2003 (*n* = 5): Ocelli count: 5/5. Ocelli present, relative size: ALMa (5/5) ≤ MLMa (5/5) ≥ PLMa (5/5) > ADMi (5/5) ≥ ≤ PDMi (5/5). Eyespot present (5/5).

*Hemilychas alexandrinus* (Hirst, 1911) (*n* = 1): Ocelli count: 5/5. Ocelli present, relative size: ALMa (1/1) < MLMa (1/1) ≥ PLMa (1/1) > PDMi (1/1) > ADMi (1/1). Eyespot present?

*Hottentotta arenaceus* (Purcell, 1901) (*n* = 2): Ocelli count: 5/5. Ocelli present, relative size: ALMa (2/2) ≤ MLMa (2/2) ≥ PLMa (2/2) > ADMi (2/2) ≥ PDMi (2/2). Eyespot present?

*Isometroides* sp. (*n* = 1): Ocelli count: 5/5. Ocelli present, relative size: ALMa (1/1) ≤ MLMa (1/1) > < PLMa (1/1) > PDMi (1/1) ≥ ADMi (1/1). Eyespot present (1/1).

*Isometrus maculatus* (DeGeer, 1778) (*n* = 3): Ocelli count: 5/5. Ocelli present, relative size: ALMa (3/2) < MLMa (3/2) ≥ ≤ PLMa (3/2) > PDMi (3/2) ≥ ADMi (3/2). Eyespot present (3/2).

*Isometrus petrzelkai* Kovařík, 2003 (*n* = 2): Ocelli count: 5/5. Ocelli present, relative size: ALMa (2/2) < MLMa (2/2) ≥ PLMa (2/2) > PDMi (2/2) > < ADMi (2/2). Eyespot present (2/2).

*Karasbergia methueni* Hewitt, 1913 (*n* = 3): Ocelli count: 3/3. Ocelli present, relative size: MLMa (3/3) > < PLMa (3/3) ≥ PDMi (3/3). Eyespot present (3/3).

*Kraepelinia palpator* (Birula, 1903) (*n* = 1): Ocelli count: 5/5. Ocelli present, relative size: ALMa (1/1) ≤ MLMa (1/1) > PLMa (1/1) > ADMi (1/1) > PDMi (1/1). Eyespot present?

*Leiurus hebraeus* (Birula, 1908) (*n* = 3): Ocelli count: 5/5. Ocelli present, relative size: ALMa (3/3) ≥ MLMa (3/3) ≥ PLMa (3/3) > ADMi (3/3) ≥ ≤ PDMi (3/3). Eyespot present?

*Liobuthus kessleri* Birula, 1898 (*n* = 3): Ocelli count: 5/5. Ocelli present, relative size: ALMa (3/3) ≤ MLMa (3/3) > PLMa (3/3) > PDMi (3/3) ≤ ADMi (3/3). Eyespot present?

*Lissothus bernardi* Vachon, 1948 (*n* = 1): Ocelli count: 4/4. Ocelli present, relative size: MLMa (1/1) > PLMa (1/1) > PDMi (1/1) > < ADMi (1/1). Eyespot present?

*Lychas burdoi* (Simon, 1882) (*n* = 6): Ocelli count: 4–5/4–5. Ocelli present, relative size: ALMa (6/6) < MLMa (6/6) > PLMa (6/6) > ADMi (6/6) > PDMi (2/2). Eyespot present (6/6).

*Lychas scutilus* C.L. Koch, 1845 (*n* = 2): Ocelli count: 5/5. Ocelli present, relative size: ALMa (2/2) < MLMa (2/2) > PLMa (2/2) > PDMi (2/2) > ADMi (2/2). Eyespot present (2/2).

*Mesobuthus caucasicus* (Nordmann, 1840) (*n* = 2): Ocelli count: 5/5. Ocelli present, relative size: ALMa (2/2) > MLMa (2/2) > PLMa (2/2) > PDMi (2/2) ≥ ≤ ADMi (2/2). Eyespot present?

*Mesotityus vondangeli* González-Sponga, 1981 (*n* = 2): Ocelli count: 4/4. Ocelli present, relative size: ALMa (2/2) < MLMa (2/2) > PLMa (2/2) > ADMi (1/2) and PDMi (1/-). Eyespot absent (2/2).

*Microananteris* sp. (*n* = 1): Ocelli count: 4/4. Ocelli present, relative size: ALMa (1/1) > MLMa (1/1) > PLMa (1/1) > ADMi (1/1). Eyespot absent (1/1).

*Microbuthus maroccanus* Lourenço, 2002 (*n* = 1): Ocelli count: 4/4. Ocelli present, relative size: MLMa (1/1) = PLMa (1/1) > PDMi (1/1) > < ADMi (1/1). Eyespot present (1/1).

*Microcharmus pauliani namoroka* Lourenço et al., 2006 (*n* = 2): Ocelli count: 5/4. Ocelli present, relative size: ALMa (2/2) ≤ MLMa (2/2) ≥ PLMa (2/2) > ADMi (2/2) > PDMi (2/-). Eyespot absent (2/2).

*Microtityus consuelo* Armas & Marcano Fondeur, 1987 (*n* = 2): Ocelli count: 4/4. Ocelli present, relative size: ALMa (2/2) < MLMa (2/2) > PLMa (2/2) > ADMi (2/2). Eyespot absent (2/2).

*Neobuthus awashensis* Kovařík & Lowe, 2012 (*n* = 1): Ocelli count: 5/5. Ocelli present, relative size: ALMa (1/1) < MLMa (1/1) = PLMa (1/1) > PDMi (1/1) > ADMi (1/1). Eyespot present (1/1).

*Neogrosphus griveaudi* (Vachon, 1969) (*n* = 3): Ocelli count: 5/5. Ocelli present, relative size: ALMa (3/3) < MLMa (3/3) ≥ ≤ PLMa (3/3) > ADMi (3/3) > < PDMi (3/3). Eyespot present (3/3).

*Odontobuthus odonturus* (Pocock, 1897) (*n* = 3): Ocelli count: 5/5. Ocelli present, relative size: ALMa (3/3) > < MLMa (3/3) > PLMa (3/3) > PDMi (3/3) ≥ ADMi (3/3). Eyespot present?

*Odonturus dentatus* Karsch, 1879 (*n* = 6): Ocelli count: 5/5. Ocelli present, relative size: ALMa (6/6) < MLMa (6/6) ≥ PLMa (6/6) > ADMi (6/6) > PDMi (6/6). Eyespot present (6/6).

*Orthochirus scrobiculosus* (Grube, 1873) (*n* = 2): Ocelli count: 5/5. Ocelli present, relative size: ALMa (2/2) ≤ MLMa (2/2) > PLMa (2/2) > ADMi (2/2) ≥ PDMi (2/2). Eyespot present (2/2).

*Parabuthus transvaalicus* Purcell, 1899 (*n* = 2): Ocelli count: 5/5. Ocelli present, relative size: ALMa (2/2) ≤ MLMa (2/2) ≥ PLMa (2/2) > PDMi (2/2) > ADMi (2/2). Eyespot present (2/2).

*Physoctonus debilis* (C.L. Koch, 1840) (*n* = 1): Ocelli count: 5/4. Ocelli present, relative size: ALMa (1/1) ≥ MLMa (1/1) = PLMa (1/1) > PDMi (1/-) = ADMi (1/1). Eyespot present (1/1).

*Pseudolychas ochraceus* (Hirst, 1911) (*n* = 3): Ocelli count: 4–5/4. Ocelli present, relative size: ALMa (2/-) < MLMa (3/3) > PLMa (3/3) > PDMi (3/3) ≥ ADMi (3/3). Eyespot present (3/3).

*Pseudouroplectes betschi* Lourenço, 1995 (*n* = 2): Ocelli count: 5/5. Ocelli present, relative size: ALMa (2/2) < MLMa (2/2) ≥ PLMa (2/2) > PDMi (2/2) = ADMi (2/2). Eyespot absent (2/2).

*Razianus zarudnyi* (Birula, 1903) (*n* = 1): Ocelli count: 4/4. Ocelli present, relative size: MLMa (1/1) < PLMa (1/1) > PDMi (1/1) > ADMi (1/1). Eyespot present?

*Rhopalurus virkkii* Santiago-Blay, 2009 (*n* = 7): Ocelli count: 5/5. Ocelli present, relative size: ALMa (7/7) ≤ MLMa (7/7) ≥ PLMa (7/7) > ADMi (7/7) ≥ PDMi (7/7). Eyespot present (7/7).

*Sassanidotus gracilis* (Birula, 1900) (*n* = 2): Ocelli count: 5/5. Ocelli present, relative size: ALMa (2/2) ≥ MLMa (2/2) > PLMa (2/2) > ADMi (2/2) > PDMi (2/2). Eyespot present (2/2).

*Thaicharmus mahunkai* Kovařík, 1995 (*n* = 1): Ocelli count: 5/5. Ocelli present, relative size: ALMa (1/1) < MLMa (1/1) > PLMa (1/1) > ADMi (1/1) > PDMi (1/1). Eyespot present (1/1).

*Tityobuthus petrae* Lourenço, 1996 (*n* = 3): Ocelli count: 5/5. Ocelli present, relative size: ALMa (3/3) ≤ MLMa (3/3) ≥ ≤ PLMa (3/3) > ADMi (3/3) ≥ PDMi (3/3). Eyespot absent (3/3).

*Tityopsis inexpectata* (Moreno, 1940) (*n* = 1): Ocelli count: 5/5. Ocelli present, relative size: AALMa (1/-) < ALMa (1/1) < MLMa (1/1) ≥ PLMa (1/1) > ADMi (1/1) > PDMi (-/1). Eyespot present (1/1).

*Tityus bahiensis* *eickstedtae* Lourenço, 1982 (*n* = 3): Ocelli count: 4–5/5. Ocelli present, relative size: ALMa (3/3) ≤ MLMa (3/3) ≥ ≤ PLMa (3/3) > ADMi (3/3) ≥ PDMi (2/3). Eyespot present?

*Uroplectes carinatus* (Pocock, 1890) (*n* = 7): Ocelli count: 5/5. Ocelli present, relative size: ALMa (7/7) < MLMa (7/7) ≥ ≤ PLMa (7/7) > PDMi (7/7) ≥ ≤ ADMi (7/7). Eyespot present (7/7).

*Vachoniolus globimanus* Levy et al., 1973 (*n* = 2): Ocelli count: 5/5. Ocelli present, relative size: ALMa (2/2) < MLMa (2/2) > < PLMa (2/2) > PDMi (2/2) > ADMi (2/2). Eyespot present?

*Zabius fuscus* (Thorell, 1876) (*n* = 2): Ocelli count: 4/4. Ocelli present, relative size: MLMa (2/2) > < PLMa (2/2) > ADMi (2/2) ≥ PDMi (2/2). Eyespot present (2/2).

**Family Chactidae Pocock, 1893**

*Broteochactas nitidus* Pocock, 1893 (*n* = 6): Ocelli count: 3–4/3–4. Ocelli present, relative size: MLMa (6/5) = PLMa (6/5) > PDMi (6/5) > ADMi (5/4). Eyespot absent (6/6).

*Brotheas granulatus* Simon, 1877 (*n* = 4): Ocelli count: 4/4. Ocelli present, relative size: ALMa (-/1) < MLMa (4/4) = PLMa (4/4) > PDMi (4/4) > ADMi (4/3). Eyespot absent (4/4).

*Brotheas wareipai* González-Sponga, 2004 (*n* = 7): Ocelli count: 3–4/4. Ocelli present, relative size: MLMa (7/7) ≤ PLMa (7/7) > PDMi (7/7) > ADMi (6/7). Eyespot absent (7/7).

*Chactas raymondhansi* Francke & Boos, 1986 (*n* = 3): Ocelli count: 2/2. Ocelli present, relative size: MLMa (3/3) > PLMa (3/3). Eyespot absent (3/3).

*Chactopsis insignis* Kraepelin, 1912 (*n* = 1): Ocelli count: 4/4. Ocelli present, relative size: MLMa (1/1) = PLMa (1/1) > PDMi (1/1) > ADMi (1/1). Eyespot absent (1/1).

*Chactopsoides anduzei* (González-Sponga, 1982) (*n* = 6): Ocelli count: 4/4. Ocelli present, relative size: MLMa (6/6) ≤ PLMa (6/6) > PDMi (6/6) > ADMi (6/6). Eyespot absent (6/6).

*Hadrurochactas machadoi* González-Sponga, 1993 (*n* = 2): Ocelli count: 4/4. Ocelli present, relative size: MLMa (2/2) = PLMa (2/2) > PDMi (2/2) > ADMi (2/2). Eyespot absent (2/2).

*Hadrurochactas* sp. (*n* = 1): Ocelli count: 4/4. Ocelli present, relative size: MLMa (1/1) = PLMa (1/1) > PDMi (1/1) > ADMi (1/1). Eyespot absent (1/1).

*Megachactops kuemoi* Ochoa et al., 2013 (*n* = 2): Ocelli count: 4/4. Ocelli present, relative size: MLMa (2/2) ≤ PLMa (2/2) > PDMi (2/2) > ADMi (2/2). Eyespot absent (2/2).

*Neochactas delicatus* (Karsch, 1879) (*n* = 2): Ocelli count: 4/3–4. Ocelli present, relative size: MLMa (2/2) = PLMa (2/2) > PDMi (2/2) > ADMi (2/1). Eyespot absent (2/2).

*Nullibrotheas allenii* (Wood, 1863) (*n* = 9): Ocelli count: 3/3. Ocelli present, relative size: MLMa (9/9) > PLMa (9/9) > PDMi (9/9). Eyespot present (4–?/5–?).

*Taurepania porosus* (Pocock, 1900) (*n* = 1): Ocelli count: 4/4. Ocelli present, relative size: MLMa (1/1) = PLMa (1/1) > PDMi (1/1) > ADMi (1/1). Eyespot absent (1/1).

*Taurepania vestigialis* González-Sponga, 1978 (*n* = 1): Ocelli count: 4/4. Ocelli present, relative size: MLMa (1/1) = PLMa (1/1) > PDMi (1/1) > ADMi (1/1). Eyespot absent (1/1).

*Teuthraustes glaber* Kraepelin, 1912 (*n* = 2): Ocelli count: 3/3. Ocelli present, relative size: MLMa (2/2) = PLMa (2/2) > PDMi (2/2). Eyespot absent (2/2).

*Vachoniochactas humboldti* (*n* = 1): Ocelli count: 4/4. Ocelli present, relative size: MLMa (1/1) = PLMa (1/1) > PDMi (1/1) > ADMi (1/1). Eyespot absent (1/1).

*Vachoniochactas lasallei* (González-Sponga, 1978) (*n* = 1): Ocelli count: 4/4. Ocelli present, relative size: MLMa (1/1) > PLMa (1/1) > PDMi (1/1) ≤ ADMi (1/1). Eyespot absent (1/1).

**Family Chaerilidae Pocock, 1893**

*Chaerilus chapmani* Vachon & Lourenço, 1985 (*n* = 1): Ocelli count: 2/1. Ocelli present, relative size: MLMa (1/-) < PLMa (1/1). Eyespot present (1/1).

*Chaerilus julietteae* Lourenço, 2011 (*n* = 5): Ocelli count: 2/2. Ocelli present, relative size: MLMa (5/5) ≥ ≤ PLMa (5/5). Eyespot present (5/5).

*Chaerilus telnovi* Lourenço, 2009 (*n* = 1): Ocelli count: 2/2. Ocelli present, relative size: MLMa (1/1) = PLMa (1/1). Eyespot present (1/1).

*Chaerilus variegatus* Simon, 1877 (*n* = 11): Ocelli count: 2–3/2–3. Ocelli present, relative size: ALMa (1/1) < MLMa (11/11) ≥ ≤ PLMa (11/11) > PLMi (4/2). Eyespot present (11/11).

**Family Diplocentridae Karsch, 1880**

*Bioculus caboensis* (Stahnke, 1968) (*n* = 1): Ocelli count: 3/3. Ocelli present, relative size: MLMa (1/1) < PLMa (1/1) = PDMi (1/1). Eyespot absent (1/1).

*Bioculus cruzensis* Stahnke, 1968 (*n* = 2): Ocelli count: 2/2. Ocelli present, relative size: PLMa (2/2) > PDMi (2/2). Eyespot absent (2/2).

*Cazierius* *neibae* Kovařík & Teruel, 2014 (*n* = 2): Ocelli count: 3/3. Ocelli present, relative size: MLMa (2/2) = PLMa (2/2) > PDMi (2/2). Eyespot absent (2/2).

*Didymocentrus hasethi* (Kraepelin, 1896) (*n* = 3): Ocelli count: 3/3. Ocelli present, relative size: MLMa (3/3) ≤ PLMa (3/3) ≥ PDMi (3/3). Eyespot absent (3/3).

*Diplocentrus rectimanus* Pocock, 1898 (*n* = 4): Ocelli count: 3/3–4. Ocelli present, relative size: MLMa (4/4) ≤ PLMa (4/4) ≥ PDMi (4/4) > APLMi_2_ (-/1). Eyespot absent (4/4).

*Heteronebo monticola* (Armas, 1999) (*n* = 2): Ocelli count: 3/3. Ocelli present, relative size: MLMa (2/2) = PLMa (2/2) ≥ PDMi (2/2). Eyespot absent (2/2).

*Heteronebo oviedo* (Armas, 1999) (*n* = 2): Ocelli count: 3/3. Ocelli present, relative size: MLMa (2/2) ≥ PLMa (2/2) ≥ PDMi (2/2). Eyespot absent (2/2).

*Kolotl poncei* (Francke & Quijana-Ravell, 2009) (*n* = 1): Ocelli count: 3/3. Ocelli present, relative size: MLMa (1/1) < PLMa (1/1) > PDMi (1/1). Eyespot absent (1/1).

*Nebo hierichonticus* (Simon, 1872) (*n* = 3): Ocelli count: 3/3–4. Ocelli present, relative size: MLMa (3/3) ≤ PLMa (3/3) > PDMi (3/3) > ADMi (-/1). Eyespot absent (3/3).

*Oiclus purvesii* (Becker, 1880) (*n* = 1): Ocelli count: 2/2. Ocelli present, relative size: PLMa (1/1) > PDMi (1/1). Eyespot absent (1/1).

*Tarsoporosus kugleri* (Schenkel, 1932) (*n* = 1): Ocelli count: 3/3. Ocelli present, relative size: MLMa (1/1) ≤ PLMa (1/1) > PDMi (1/1). Eyespot absent (1/1).

*Tarsoporosus macuira* Teruel & Roncallo, 2010 (*n* = 2): Ocelli count: 3–4/3. Ocelli present, relative size: MLMa (2/2) ≥ PLMa (2/2) > PDMi (2/2) > PLMi (1/-). Eyespot absent (2/2).

**Family Euscorpiidae Laurie, 1896**

*Euscorpius italicus* (Herbst, 1800) (*n* = 1): Ocelli count: 3/3. Ocelli present, relative size: MLMa (1/1) > PLMa (1/1) > ADMi (1/1). Eyespot absent (1/1).

*Euscorpius tergestinus* (C.L. Koch, 1837) (*n* = 1): Ocelli count: 3/3. Ocelli present, relative size: MLMa (1/1) > PLMa (1/1) > ADMi (1/1). Eyespot absent (1/1).

*Megacormus gertschi* Díaz Najera, 1966 (*n* = 2): Ocelli count: 4/4. Ocelli present, relative size: MLMa (2/2) ≥ PLMa (2/2) > ADMi (2/2) > PDMi (2/2). Eyespot absent (2/2).

*Plesiochactas dilutus* (Karsch, 1881) (*n* = 1): Ocelli count: 2/2. Ocelli present, relative size: MLMa (1/1) > PLMa (1/1). Eyespot absent (1/1).

*Plesiochactas mitchelli* Soleglad, 1976 (*n* = 1): Ocelli count: 2/2. Ocelli present, relative size: MLMa (1/1) > PLMa (1/1). Eyespot absent (1/1).

*Troglocormus ciego* Francke, 1981 (*n* = 1): Ocelli count: 4/4. Ocelli present, relative size: MLMa (1/1) = PLMa (1/1) > PDMi (1/1) ≥ ADMi (1/1). Eyespot absent (1/1).

*Troglocormus willis* Francke, 1981 (*n* = 3): Ocelli count: 2–3/2–3. Ocelli present, relative size: MLMa (3/3) ≥ PLMa (3/3) > ADMi (1/2). Eyespot absent (3/3).

**Family Hemiscorpiidae Pocock, 1893**

*Hemiscorpius lepturus* Peters, 1861 (*n* = 2): Ocelli count: 3/3. Ocelli present, relative size: MLMa (2/2) ≥ PLMa (2/2) > PDMi (2/2). Eyespot absent (2/2).

**Family Heteroscorpionidae Kraepelin, 1905**

*Heteroscorpion kraepelini* Lourenço & Goodman, 2006 (*n* = 2): Ocelli count: 2–3/2: Ocelli present, relative size: PLMa (2/2) > PDMi (2/2) > ADMi (1/-). Eyespot absent (2/2).

*Heteroscorpion magnus* Lourenço & Goodman, 2002 (*n* = 3): Ocelli count: 4–5/2–3. Ocelli present, relative size: PLMa (3/3) > PDMi (3/3) > PLMi (3/1) ≥ APLMi_1_ (3/-) = ADMi (1/1). Eyespot absent (3/3).

*Heteroscorpion* sp. (*n* = 3): Ocelli count: 2/2. Ocelli present, relative size: PLMa (3/3) > PDMi (3/3). Eyespot absent (3/3).

**Family Hormuridae Laurie, 1896**

*Cheloctonus jonesii* Pocock, 1892 (*n* = 5): Ocelli count: 3/3–4. Ocelli present, relative size: MLMa (5/5) ≥ PLMa (5/5) ≥ PDMi (5/5) > PLMi (-/2). Eyespot absent (5/5).

*Chiromachetes fergusoni* Pocock, 1899 (*n* = 1): Ocelli count: 3/3. Ocelli present, relative size: MLMa (1/1) = PLMa (1/1) > PDMi (1/1). Eyespot absent (1/1).

*Chiromachus ochropus* (C.L. Koch, 1837) (*n* = 1): Ocelli count: 3/3. Ocelli present, relative size: MLMa (1/1) = PLMa (1/1) > PDMi (1/1). Eyespot absent (1/1).

*Hadogenes troglodytes* (Peters, 1861) (*n* = 2): Ocelli count: 3/3. Ocelli present, relative size: MLMa (2/2) ≤ PLMa (2/2) ≥ PDMi (2/2). Eyespot absent (2/2).

*Hormiops davidovi* Fage, 1933 (*n* = 6): Ocelli count: 2/2. Ocelli present, relative size: PLMa (6/6) > PDMi (6/6). Eyespot absent (6/6).

*Hormurus extensus* (Locket, 1997) (*n* = 1): Ocelli count: 3/3. Ocelli present, relative size: MLMa (1/1) > PLMa (1/1) ≥ PDMi (1/1). Eyespot absent (1/1).

*Iomachus laeviceps* (Pocock, 1890) (*n* = 6): Ocelli count: 3–4/3. Ocelli present, relative size: MLMa (6/6) ≥ ≤ PLMa (6/6) > PDMi (6/6) > PLMi (1/-). Eyespot absent (6/6).

*Iomachus politus* Pocock, 1896 (*n* = 3): Ocelli count: 3/3. Ocelli present, relative size: MLMa (3/3) ≥ PLMa (3/3) > PDMi (3/3). Eyespot absent (3/3).

*Liocheles australasiae* (Fabricius, 1775) (*n* = 3): Ocelli count: 3/3. Ocelli present, relative size: MLMa (3/3) ≤ PLMa (3/3) > PDMi (3/3). Eyespot absent (3/3).

*Opisthacanthus elatus* (Gervais, 1844) (*n* = 1): Ocelli count: 3/3. Ocelli present, relative size: MLMa (1/1) > PLMa (1/1) > PDMi (1/1). Eyespot absent (1/1).

*Opisthacanthus lecomtei* (Lucas, 1858) (*n* = 1): Ocelli count: 3/3. Ocelli present, relative size: MLMa (1/1) = PLMa (1/1) > PDMi (1/1). Eyespot absent (1/1).

*Opisthacanthus madagascariensis* Kraepelin, 1894 (*n* = 1): Ocelli count: 3/3. Ocelli present, relative size: MLMa (1/1) > PLMa (1/1) > PDMi (1/1). Eyespot absent (1/1).

*Opisthacanthus validus* Thorell, 1876 (*n* = 2): Ocelli count: 3/3. Ocelli present, relative size: MLMa (2/2) = PLMa (2/2) = PDMi (2/2). Eyespot absent (2/2).

*Palaeocheloctonus pauliani* Lourenço, 1996 (*n* = 5): Ocelli count: 3/3. Ocelli present, relative size: MLMa (5/5) ≥ PLMa (5/5) ≥ PDMi (5/5). Eyespot absent (5/5).

**Family Iuridae Thorell, 1876**

*Anuroctonus phaiodactylus* (Wood, 1863) (*n* = 1): Ocelli count: 4/4. Ocelli present, relative size: MLMa (1/1) > PLMa (1/1) > PDMi (1/1) > ADMi (1/1). Eyespot absent (1/1).

*Calchas anlasi* Yağmur et al., 2013 (*n* = 1): Ocelli count: 4/3. Ocelli present, relative size: MLMa (1/1) > PLMa (1/1) > ADMi (1/1) > PDMi (1/-). Eyespot absent (1/1).

*Calchas birulai* Fet et al., 2009 (*n* = 4): Ocelli count: 4/4. Ocelli present, relative size: MLMa (4/4) > < PLMa (4/4) > PDMi (4/4) ≥ ADMi (4/4). Eyespot absent (4/4).

*Caraboctonus keyserlingi* Pocock, 1893 (*n* = 4): Ocelli count: 4/4. Ocelli present, relative size: MLMa (4/4) > PLMa (4/4) > PDMi (4/4) > ADMi (4/4). Eyespot present (4/4).

*Hadruroides charcasus* (Karsch, 1879) (*n* = 5): Ocelli count: 4/4. Ocelli present, relative size: MLMa (5/5) > PLMa (5/5) > PDMi (5/5) > ADMi (5/5). Eyespot present (5/5).

*Hadrurus arizonensis* Ewing, 1928 (*n* = 3): Ocelli count: 3/3. Ocelli present, relative size: MLMa (3/3) > PLMa (3/3) > PDMi (3/3). Eyespot present (3/3).

*Hoffmannihadrurus aztecus* (Pocock, 1902) (*n* = 2): Ocelli count: 3/3. Ocelli present, relative size: MLMa (2/2) > PLMa (2/2) > PDMi (2/2). Eyespot present (2/2).

*Hoffmannihadrurus gertschi* (Soleglad, 1976) (*n* = 1): Ocelli count: 4/4. Ocelli present, relative size: MLMa (1/1) > PLMa (1/1) > PDMi (1/1) > ADMi (1/1). Eyespot present (1/1).

*Iurus dufoureius* (Brullé, 1832) (*n* = 1): Ocelli count: 4/4. Ocelli present, relative size: MLMa (1/1) > PLMa (1/1) > PDMi (1/1) ≥ ADMi (1/1). Eyespot absent (1/1).

*Neocalchas gruberi* (Fet et al., 2009) (*n* = 1): Ocelli count: 4/3. Ocelli present, relative size: MLMa (1/1) > PLMa (1/1) > PDMi (1/1) = ADMi (1/-). Eyespot absent (1/1).

*Protoiurus kraepelini* (von Ubisch, 1922) (*n* = 5): Ocelli count: 4/4. Ocelli present, relative size: MLMa (5/5) ≥ ≤ PLMa (5/5) > PDMi (5/5) ≥ ≤ ADMi (5/5). Eyespot absent (5/5).

**Family Pseudochactidae Gromov, 1998**

*Pseudochactas ovchinnikovi* Gromov, 1998 (*n* = 12): Ocelli count: 1/1. Ocelli present, relative size: PLMa (12/12). Eyespot absent (12/12).

*Troglokhammouanus steineri* Lourenço, 2007 (*n* = 8): Ocelli count: 1/1. Ocelli present, relative size: PLMa (8/8). Eyespot absent (8/8).

*Vietbocap lao* Lourenço, 2012 (*n* = 2): Ocelli count: 0/0. Eyespot absent (2/2).

**Family Scorpionidae Latreille, 1802**

*Heterometrus cyaneus* (C.L. Koch, 1836) (*n* = 3): Ocelli count: 4/4–5. Ocelli present, relative size: MLMa (3/3) < PLMa (3/3) > PDMi (3/3) > ADMi (-/1) < PLMi (3/3) > APLMi_2_ (-/1). Eyespot absent (3/3).

*Opistophthalmus jenseni* (Lamoral, 1972) (*n* = 12): Ocelli count: 3–7/3–7. Ocelli present, relative size: MLMa (12/12) < PLMa (12/12) ≥ PDMi (12/12) > ADMi (1/1) = APLMi_2_ (7/4) ≥ ≤ PLMi (10/9) ≥ APLMi_3_ (3/3) ≥ APLMi_1_ (2/3). Eyespot absent (12/12).

*Opistophthalmus* sp. (*n* = 12): Ocelli count: 3–5/4–5. Ocelli present, relative size: MLMa (12/12) ≤ PLMa (12/12) > PDMi (12/12) > ADMi (2/1) < PLMi (11/12) = APLMi_2_ (1/2). Eyespot absent (12/12).

*Pandinus gregoryi* (Pocock, 1896) (*n* = 6): Ocelli count: 3–4/3–4. Ocelli present, relative size: MLMa (6/6) ≥ ≤ PLMa (6/6) > PDMi (6/6) > PLMi (2/2). Eyespot absent (6/6).

*Scorpio maurus palmatus* (Ehrenberg, 1828) (*n* = 4): Ocelli count: 3–4/3–4. Ocelli present, relative size: MLMa (4/4) = PLMa (4/4) > PDMi (4/4) > PLMi (2/2). Eyespot absent (4/4).

**Family Scorpiopidae Kraepelin, 1905**

*Alloscorpiops* sp. (*n* = 1): Ocelli count: 4/4. Ocelli present, relative size: MLMa (1/1) > PLMa (1/1) > PDMi (1/1) > PLMi (1/1). Eyespot absent (1/1).

*Euscorpiops kaftani* (Kovařík, 1993) (*n* = 1): Ocelli count: 4/4. Ocelli present, relative size: MLMa (1/1) = PLMa (1/1) > PDMi (1/1) > PLMi (1/1). Eyespot absent (1/1).

*Euscorpiops problematicus* Kovařík, 2000 (*n* = 1): Ocelli count: 4/4. Ocelli present, relative size: MLMa (1/1) = PLMa (1/1) > PDMi (1/1) > PLMi (1/1). Eyespot absent (1/1).

*Parascorpiops montana* Banks, 1928 (*n* = 6): Ocelli count: 2–3/2–3. Ocelli present, relative size: MLMa (6/6) ≥ PLMa (6/6) > PDMi (4/4). Eyespot absent (6/6).

*Scorpiops feti* Kovařík, 2000 (*n* = 2): Ocelli count: 4/4. Ocelli present, relative size: MLMa (2/2) > PLMa (2/2) > PDMi (2/2) > PLMi (2/2). Eyespot absent (2/2).

**Family Superstitioniidae Stahnke, 1940**

*Superstitionia donensis* Stahnke, 1940 (*n* = 4): Ocelli count: 4/4. Ocelli present, relative size: MLMa (4/4) > PLMa (4/4) > PDMi (4/4) ≥ ≤ ADMi (4/4). Eyespot absent (4/4).

**Family Troglotayosicidae Lourenço, 1998**

*Belisarius xambeui* Simon, 1879 (*n* = 2): Ocelli count: 0/0. Eyespot present?

*Troglotayosicus humiculum* Botero-Trujillo & Francke, 2009 (*n* = 3): Ocelli count: 2/2. Ocelli present, relative size: PLMa (3/3) > PDMi (3/3). Eyespot present (3/3).

**Family Typhlochactidae Mitchell, 1971**

*Alacran tartarus* Francke, 1982 (*n* = 1): Ocelli count: 0/0. Eyespot absent (1/1).

*Alacran* sp. (*n* = 1): Ocelli count: 0/0. Eyespot absent (1/1).

*Sotanochactas elliotti* (Mitchell, 1971) (*n* = 2): Ocelli count: 0/0. Eyespot absent (2/2).

*Stygochactas granulosus* (Sissom & Cokendolpher, 1998) (*n* = 1): Ocelli count: 0/0. Eyespot absent (1/1).

*Typhlochactas mitchelli* Sissom, 1988 (*n* = 1): Ocelli count: 0/0. Eyespot absent (1/1).

*Typhlochactas rhodesi* Mitchell, 1968 (*n* = 1): Ocelli count: 0/0. Eyespot absent (1/1).

**Family Urodacidae Pocock, 1893**

*Aops oncodactylus* Volschenk & Prendini, 2008 (*n* = 1): Ocelli count: 0/0. Eyespot absent (1/1).

*Urodacus* *manicatus* (Thorell, 1876) (*n* = 2): Ocelli count: 2/2. Ocelli present, relative size: PLMa (2/2) > PDMi (2/2). Eyespot absent (2/2).

*Urodacus* sp. (*n* = 2): Ocelli count: 2/2. Ocelli present, relative size: PLMa (2/2) > PDMi (2/2). Eyespot absent (2/2).

**Family Vaejovidae Thorell, 1876**

*Chihuahuanus globosus* (Borelli, 1915) (*n* = 3): Ocelli count: 3/3. Ocelli present, relative size: MLMa (3/3) > PLMa (3/3) > PDMi (3/3). Eyespot present?

*Franckeus nitidulus* (C.L. Koch, 1843) (*n* = 3): Ocelli count: 3/3. Ocelli present, relative size: MLMa (3/3) ≥ PLMa (3/3) > PDMi (3/3). Eyespot present (3/3).

*Gertschius crassicorpus* Graham & Soleglad, 2007 (*n* = 5): Ocelli count: 3/3. Ocelli present, relative size: MLMa (5/5) ≥ PLMa (5/5) > PDMi (5/5). Eyespot present (4–?/2–?).

*Kochius bruneus* (Williams, 1970) (*n* = 5): Ocelli count: 3/3. Ocelli present, relative size: MLMa (5/5) ≥ PLMa (5/5) > PDMi (5/5). Eyespot present (5/4).

*Kovarikia angelenus* (Gertsch & Soleglad, 1972) (*n* = 1): Ocelli count: 3/3. Ocelli present, relative size: MLMa (1/1) > PLMa (1/1) > PDMi (1/1). Eyespot absent (1/1).

*Kuarapu purhepecha* Francke & Ponce-Saavedra, 2010 (*n* = 2): Ocelli count: 3/3. Ocelli present, relative size: MLMa (2/2) > PLMa (2/2) > PDMi (2/2). Eyespot present?

*Maaykuyak vittatus* (Williams, 1970) (*n* = 3): Ocelli count: 3/3. Ocelli present, relative size: MLMa (3/3) > PLMa (3/3) > PDMi (3/3). Eyespot present (3/3).

*Mesomexovis oaxaca* (Santibañez-Lopez & Sissom, 2010) (*n* = 3): Ocelli count: 3/3. Ocelli present, relative size: MLMa (3/3) > PLMa (3/3) > PDMi (3/3). Eyespot present (3/3).

*Paravaejovis pumilis* (Williams, 1970) (*n* = 3): Ocelli count: 3/3. Ocelli present, relative size: MLMa (3/3) > PLMa (3/3) > PDMi (3/3). Eyespot present?

*Paruroctonus surensis* Williams & Haradon, 1980 (*n* = 3): Ocelli count: 3/3. Ocelli present, relative size: MLMa (3/3) > PLMa (3/3) > PDMi (3/3). Eyespot present (3/3).

*Pseudouroctonus apacheanus* (Gertsch & Soleglad, 1972) (*n* = 2): Ocelli count: 3/3–4. Ocelli present, relative size: MLMa (2/2) > PLMa (2/2) > PDMi (2/2) > ADMi (-/1). Eyespot absent (2/2).

*Pseudouroctonus reddelli* (Gertsch & Soleglad, 1972) (*n* = 1): Ocelli count: 3/3. Ocelli present, relative size: MLMa (1/1) > PLMa (1/1) > PDMi (1/1). Eyespot present (1/1).

*Serradigitus wupatkiensis* (Stahnke, 1940) (*n* = 2): Ocelli count: 3/3. Ocelli present, relative size: MLMa (2/2) > PLMa (2/2) > PDMi (2/2). Eyespot present (2/2).

*Smeringurus grandis* (Williams, 1970) (*n* = 3): Ocelli count: 3/3. Ocelli present, relative size: MLMa (3/3) > PLMa (3/3) > PDMi (3/3). Eyespot present?

*Stahnkeus subtilimanus* (Soleglad, 1972) (*n* = 1): Ocelli count: 3/3. Ocelli present, relative size: MLMa (1/1) > PLMa (1/1) > PDMi (1/1). Eyespot present (1/1).

*Syntropis macrura* Kraepelin, 1900 (*n* = 2): Ocelli count: 3/3. Ocelli present, relative size: MLMa (2/2) > PLMa (2/2) > PDMi (2/2). Eyespot present (1–?/1–?).

*Thorellius intrepidus* (Thorell, 1876) (*n* = 3): Ocelli count: 3/3. Ocelli present, relative size: MLMa (3/3) > PLMa (3/3) > PDMi (3/3). Eyespot present (3/3).

*Uroctonites giulianii* Williams & Savary, 1991 (*n* = 1): Ocelli count: 3/3. Ocelli present, relative size: MLMa (1/1) > PLMa (1/1) > PDMi (1/1). Eyespot absent (1/1).

*Uroctonus mordax* Thorell, 1876 (*n* = 2): Ocelli count: 3/3. Ocelli present, relative size: MLMa (2/2) > PLMa (2/2) > PDMi (2/2). Eyespot absent (2/2).

*Uroctonus mordax pluridens* Hjelle, 1972 (*n* = 2): Ocelli count: 3–4/3–4. Ocelli present, relative size: MLMa (2/2) ≥ PLMa (2/2) > PDMi (2/2) ≥ ADMi (1/1). Eyespot absent (2/2).

*Vaejovis carolinianus* (Beauvois, 1805) (*n* = 2): Ocelli count: 3/3. Ocelli present, relative size: MLMa (2/2) ≥ PLMa (2/2) > PDMi (2/2). Eyespot present (2/2).

*Vaejovis mexicanus* C.L. Koch, 1836 (*n* = 1): Ocelli count: 3/3. Ocelli present, relative size: MLMa (1/1) > PLMa (1/1) > PDMi (1/1). Eyespot absent (1/1).

*Vaejovis vorhiesi* Stahnke, 1940 (*n* = 2): Ocelli count: 3/3. Ocelli present, relative size: MLMa (2/2) > PLMa (2/2) > PDMi (2/2). Eyespot present (2/2).

*Vejovoidus longiunguis* (Williams, 1969) (*n* = 3): Ocelli count: 3/3. Ocelli present, relative size: MLMa (3/3) > PLMa (3/3) > PDMi (3/3). Eyespot present?

*Vizcaino viscainensis* (Williams, 1970) (*n* = 2): Ocelli count: 3/3. Ocelli present, relative size: MLMa (2/2) > PLMa (2/2) > PDMi (2/2). Eyespot present?

*Wernerius mumai* (Sissom, 1993) (*n* = 2): Ocelli count: 2–3/3. Ocelli present, relative size: MLMa (2/2) ≥ PLMa (2/2) > PDMi (1/2). Eyespot absent (2/2).
